# Understanding Factors that Shape Gender Attitudes in Early Adolescence Globally: A Mixed-Methods Systematic Review

**DOI:** 10.1371/journal.pone.0157805

**Published:** 2016-06-24

**Authors:** Anna Kågesten, Susannah Gibbs, Robert Wm Blum, Caroline Moreau, Venkatraman Chandra-Mouli, Ann Herbert, Avni Amin

**Affiliations:** 1 Department of Population, Family and Reproductive Health, Johns Hopkins School of Public Health, Baltimore, Maryland, United States of America; 2 WHO Department of Reproductive Health and Research, including UNDP/UNFPA/UNICEF/WHO/World Bank Special Programme of Research, Development and Research Training in Human Reproduction, World Health Organization, Geneva, Switzerland; University of Westminster, UNITED KINGDOM

## Abstract

**Background:**

Early adolescence (ages 10–14) is a period of increased expectations for boys and girls to adhere to socially constructed and often stereotypical norms that perpetuate gender inequalities. The endorsement of such gender norms is closely linked to poor adolescent sexual and reproductive and other health-related outcomes yet little is known about the factors that influence young adolescents’ personal gender attitudes.

**Objectives:**

To explore factors that shape gender attitudes in early adolescence across different cultural settings globally.

**Methods:**

A mixed-methods systematic review was conducted of the peer-reviewed literature in 12 databases from 1984–2014. Four reviewers screened the titles and abstracts of articles and reviewed full text articles in duplicate. Data extraction and quality assessments were conducted using standardized templates by study design. Thematic analysis was used to synthesize quantitative and qualitative data organized by the social-ecological framework (individual, interpersonal and community/societal-level factors influencing gender attitudes).

**Results:**

Eighty-two studies (46 quantitative, 31 qualitative, 5 mixed-methods) spanning 29 countries were included. Ninety percent of studies were from North America or Western Europe. The review findings indicate that young adolescents, across cultural settings, commonly express stereotypical or inequitable gender attitudes, and such attitudes appear to vary by individual sociodemographic characteristics (sex, race/ethnicity and immigration, social class, and age). Findings highlight that interpersonal influences (family and peers) are central influences on young adolescents’ construction of gender attitudes, and these gender socialization processes differ for boys and girls. The role of community factors (e.g. media) is less clear though there is some evidence that schools may reinforce stereotypical gender attitudes among young adolescents.

**Conclusions:**

The findings from this review suggest that young adolescents in different cultural settings commonly endorse norms that perpetuate gender inequalities, and that parents and peers are especially central in shaping such attitudes. Programs to promote equitable gender attitudes thus need to move beyond a focus on individuals to target their interpersonal relationships and wider social environments. Such programs need to start early and be tailored to the unique needs of sub-populations of boys and girls. Longitudinal studies, particularly from low-and middle-income countries, are needed to better understand how gender attitudes unfold in adolescence and to identify the key points for intervention.

## Introduction

Adolescence (10–19 years) is a critical period of rapid physical and psychosocial changes, exposing adolescents to sexual and reproductive health risks and opportunities [1–3]. It is also during adolescence that sex-differential mortality and morbidity patterns begin to emerge [1,4]. Specifically, for girls pregnancy complications associated with early pregnancy, childbearing and unsafe abortion, HIV/AIDS and infectious diseases all account for significant mortality [1]. Girls are also more likely than boys to be married as children [5] and to experience forced sexual initiation [6]. It has been estimated that 29% of adolescent girls aged 15–19 years report lifetime physical and/or sexual violence by an intimate partner [7]. On the other hand, for adolescent boys, the top causes of mortality include unintentional injuries from road injuries and interpersonal violence, HIV/AIDS, suicide and drowning [1]. In many societies, boys also engage in more health harming behaviors than girls such as early and heavy smoking, alcohol and illicit drug use [5] and are more likely than girls to engage in early and unprotected sexual behaviors [8].

While there are many factors that explain sex differentials in mortality and morbidity, a key determinant is gender inequality. Gender inequalities manifest in different ways, such as unequal access to resources, power, education and discriminatory socio-cultural practices [9]. While gender inequalities affect the lives of both boys and girls, generally they disproportionately disadvantage girls. At the root of many gender inequalities are gender norms that prescribe different status, power and opportunities to girls and boys according to culturally appropriate versions of masculinities and femininities [10]. We refer to these as *inequitable*, *unequal or harmful stereotypical gender norms* and use the terms interchangeably (as defined further below). In every cultural setting across time and place individuals are socialized overtly and covertly from birth to conform to rules for how to “be” girls and boys [10–12]. These gender norms shape the way adolescents interact, form relationships, and engage in sexual and reproductive practices as well as most all social behaviors.

Global data indicate that gender norms are commonly reflected in adolescents’ personal *gender attitudes*. For example, population-based surveys in low- and middle-income countries (LMICs) indicate that over half of boys and girls aged 15–19 years justify wife beating under certain conditions [5]. Studies conducted with young men from LMICs further reflect the complexity of gender attitudes where some might eschew harmful gender discriminatory practices but at the same time endorse unequal gender division of labor in the household or other inequitable gender norms [13,14]. Gender attitudes that endorse norms that perpetuate gender inequality are thought to be harmful to both boys and girls. Among young men, endorsement of stereotypical masculinity norms prescribing male dominance and toughness have been associated with substance use, violence and delinquency [15–17], lower male engagement in caregiving and household chores, unsafe sexual behaviors, multiple sexual partners [18,19,20], higher fertility aspirations, lower rates of male sexual satisfaction, and perpetration of intimate partner violence [21–25]. Conversely, young women and girls are often under pressure to conform to stereotypical norms of female subordination, thus restricting their voice, opportunities and social and sexual decision-making [22].

While gender socialization starts at birth, *early adolescence* (ages 10–14) is a critical point of intensification in personal gender attitudes as puberty reshapes male and female self-perceptions, as well as social expectations from others (e.g. family members, peers) [26]. With puberty freedom of movement may become more constrained for girls, especially in LMICs, as they are expected to take on more household chores, marry and/or stay away from boys due to adult concerns about their developing bodies and emerging sexuality, whereas boys often experience greater freedom to move outside of the household and engage in leisure activities while also facing increased exposures to environmental risks as well as expectations to work and help support the family financially [27]. Even where it is not socially sanctioned, romantic and sexual feelings begin to emerge and gender roles play out as young people begin to negotiate intimate relationships [28]. Early adolescence is thus seen as a unique opportunity to address gender attitudes before they become more solidified [4,27,29–31].

However, there has yet to be a synthesis of factors that influence gender attitudes during this stage of life. Such knowledge can enable the design and implementation of programs and policies that address harmful stereotypical norms or promote equitable gender norms and in turn improve adolescent sexual and reproductive and other health outcomes.

In response to this gap, the current systematic review seeks to explore the factors that shape young adolescents’ gender attitudes across different cultural and geographical settings. We applied a mixed-methods approach, guided by two key research questions:

- What factors appear to be associated with gender attitudes in early adolescence? (Quantitative studies)
- How do young adolescents learn about and construct gender attitudes in relation to their social environments? (Qualitative studies)

We used the Blum et al [4] conceptual framework for early adolescence to organize, analyze and present the findings. Building on Bronfenbrenner’s social-ecological model [32], this framework recognizes that personal attitudes and behaviors are influenced by multiple factors across different interacting domains, including *individual* (e.g. sociodemographics), *interpersonal relationship* (e.g. family, peers) and *community/societal* (e.g. school, media) levels.

### Defining gender attitudes

In the current review gender is viewed as the social and cultural construction of masculine and feminine identities, roles, norms and relationships, rather than an immutable personality trait grounded in biological sex [9,16,17,33,34]. We thus view children and adolescents as actively involved with defining or challenging the social constructions of masculinity and femininity through interactions with their social and cultural environments. We refer to *gender norms* as the widely accepted social rules about roles, traits, behaviors status and power associated with masculinity and femininity in a given culture [10]. In this review, we focus on personal *gender attitudes*, which we define as the individual perceptions, beliefs or endorsement of gender norms [10] (e.g. “It’s alright for a man to beat his wife”) [35]. It is important to note that not all gender norms and attitudes are harmful. While what is considered as typical or dominant norms about masculinities and femininities vary both within and across time and settings [15–17], in this paper we are particularly interested in the factors related to attitudes that perpetuate unequal power relation between men and women or that stigmatize those who do not ascribe to culturally defined norms (e.g. boys that act “feminine”) [17,36]. As noted above, we refer to such attitudes as harmful *stereotypical*, *inequitable or unequal* gender attitudes throughout the paper and use the terms interchangeably.

## Methods

The present systematic review is structured in accordance with a modified version of the *Enhancing Transparency in Reporting the Synthesis of Qualitative Research* (ENTREQ) guidelines [37]. The methods for screening, study selection and synthesis of data were outlined in a protocol, available in S1 Text.

### Search strategy

We searched the peer-reviewed literature in 12 databases: PubMed, Psychinfo, EMBASE/MEDLINE, Scopus, ERIC, Global Health, LILACS, Sociological Abstracts, IMSEAR, AIM, IMEMR and WPRIM. Searches were conducted on August 1^st^ and 2^nd^ 2014, with no date or language restrictions. We built our search strategy in three blocks using controlled vocabulary and free-text terms: 1) young adolescents (example: adolescent, child, middle school) AND 2) gender attitudes (e.g. gender attitudes, gender stereotypes) AND 3) factors that influence gender attitudes (example: socialization, interpersonal relationship, parent influence, peer influence). We applied an inclusive approach to search terms related to gender attitudes so as to capture studies that explored this concept but used other terminologies such as “gender identity”, “femininities”, “sex role”, “gender bias” or “gender ideologies”. S1 Table shows the full search strategy for each database.

### Study selection

Three independent reviewers (AK, SG, AH) divided all records and screened the title and abstracts. Abstracts that passed the initial screen were promoted to full-text review. Each full-text was assessed by two of the reviewers using the following inclusion and exclusion criteria:

1. Primary data analysis. For purposes of this review, we limited inclusion to studies with a primary analysis of data.
2. Published between 1984 and 2014. Given that postmodern feminist theories on the social construction of gender that are relevant for studying gender norms were elaborated during the last two decades of the 20^th^ century (see for example the work by West and Zimmerman [16] and Butler [33]), the review was restricted to studies published in the last 30 years. During the initial search done without any date restrictions, we also found that articles published earlier were largely outdated in terms of their conceptualization of gender, focusing solely on biological sex differences.
3. Study population aged 10 to 14 years (or broader with age sub-groups disaggregated).
4. Focused on personal gender attitudes as the key outcome or phenomenon of interest (using the definition of gender attitudes outlined earlier)
5. Explored at least one factor that might shape gender attitudes (e.g., family, peer or school-related factors).
6. Published in a peer-reviewed journal. While we initially planned to search the grey literature (given that programmatic evaluations and studies from LMICs in particular may not reach the peer-reviewed stage) our time and financial resources did not permit us to explore this literature.

Three reviewers (GNR, MY, NS) assisted with the review of full-texts in languages other than English. All qualitative and quantitative study designs were eligible for inclusion. Assessment discrepancies were resolved by team discussion until consensus was reached.

### Data extraction

Four reviewers (AK, SG, AD, GNR) extracted data using a standardized template across the following domains: research question, study design, sampling and sample characteristics, data collection, analysis, key findings, limitations and conclusions (S2 Text). We also extracted detailed information on outcome and exposure variables from quantitative studies, and the phenomenon under investigation from qualitative studies. All extracted data were verified by two of the reviewers (AK, SG); discrepancies were resolved through discussion within the team.

### Quality assessment

Quality was assessed separately for quantitative and qualitative studies. For quantitative studies, we used a modified version of the *Effective Public Health Practice Project* (EPHPP) checklist [38], in which each study was rated as strong, moderate, weak or unclear in relation to eight criteria: 1) study design, 2) selection bias, 3) drop-outs, 4) blinding, 5) intervention integrity (if applicable), 6) data collection, and 7) analysis and confounding. For qualitative studies we assessed quality by using an adapted version of the *Critical Appraisal Skill Programme* (CASP) guide [39], rating studies as strong, moderate, weak or unclear based on nine criteria: 1) aims, 2) methodology, 3) link to theory, 4) study design, 5) fieldwork procedures, 6) data analysis, 7) credibility of findings, 8) reflexivity and 9) ethical considerations. For both quantitative and qualitative studies, four reviewers (AK, SG, AD, GNR) independently assessed the overall quality of each study by summarizing the section ratings for each criteria into a global rating for the study as strong (no weak ratings), moderate (one or two weak ratings), or low (three or more weak or unclear ratings) quality. Two reviewers (AK, SG) validated these global ratings and any discrepancies were resolved through discussions amongst the reviewers.

### Synthesis

We used a mixed-methods synthesis [40] where we first synthesized all studies separately according to their design (qualitative *vs*. quantitative), followed by an overarching synthesis across methodologies [40,41].

#### Quantitative synthesis

For the quantitative studies we conducted a thematic summary where we first clustered the factors associated with gender attitudes into categories (e.g. ethnicity, parental education). For each identified factor, we assessed the number of studies that found significant, mixed and no associations. Next, we looked for common associations across studies and summarized these as themes organized by the levels of the socio-ecological framework described by Blum et al [42]. For example, themes related to variations in gender attitudes across factors such as biological sex, age and pubertal development were organized at the individual level, while the interpersonal relationship level included findings that addressed themes related to the association between gender attitudes and parental, sibling and peer factors. Finally, we assessed the robustness of the quantitative synthesis by evaluating the number and relative quality of the studies for each theme.

#### Qualitative synthesis

For the qualitative studies we used thematic synthesis [42] to analyze the data reported in the studies. First, using Atlas.ti 7® software (Atlas Corporation, Berlin), we conducted open-ended coding on each text-unit (e.g. sentence or paragraphs) of the included studies. We focused on coding the “raw” participant data such as quotes, but also coded analyses and conclusions made by study authors as qualitative findings reported often reflects the authors’ own interpretations [41]. The first round of coding generated 11 initial broad themes (i.e. concepts identified in more than one study), for example “attitudes about femininity norms” or “parental influences”. Through an iterative process, these themes were subsequently broken into more refined codes and themes (see S3 Text). In this process, similar codes were grouped together into descriptive themes, which in turn were grouped into analytical themes at a higher level [41,42]. For example, codes labeled “boys stigmatize non-stereotypical masculinities” and “boys use humor to enforce masculine norms” were organized under the descriptive theme of “peer harassment”, which in turn was structured by the overarching analytical theme of “peers are central in establishing and upholding gender norms”.

We assessed the robustness of the qualitative themes using the Confidence in the Evidence from Reviews of Qualitative Research (CERQual) approach [43,44], a tool for evaluating the level of confidence to place in findings from qualitative syntheses. In CERQual, confidence in the evidence is evaluated in four domains which we applied as follows: 1) *methodological limitations of each primary study* (evaluated using the CASP guideline which rated studies as high, moderate or low quality as explained above); 2) *relevance of each study to the review question* (how closely the research questions of primary studies matched those of the current review); 3) *coherence of the individual study findings* (whether or not findings displayed consistent patterns across studies); and 4) *adequacy of the data contributing to a review finding* (the number of studies contributing to a finding as well as the overall richness of the data provided) [43]. Together these four components formed the basis for our overall judgment of confidence in the evidence of each review finding as: high confidence/strong evidence (grounded in several studies of high quality and relevance, “thick” coherent data across different geographical or income settings); moderate confidence/some evidence (some studies of moderate or high quality and relevance, consistent findings across more than one geographical or income setting) or low confidence/weak evidence (few studies of low or moderate quality and relevance, less coherent findings limited to similar geographical or income setting).

## Results

### General overview

**Fig 1:**
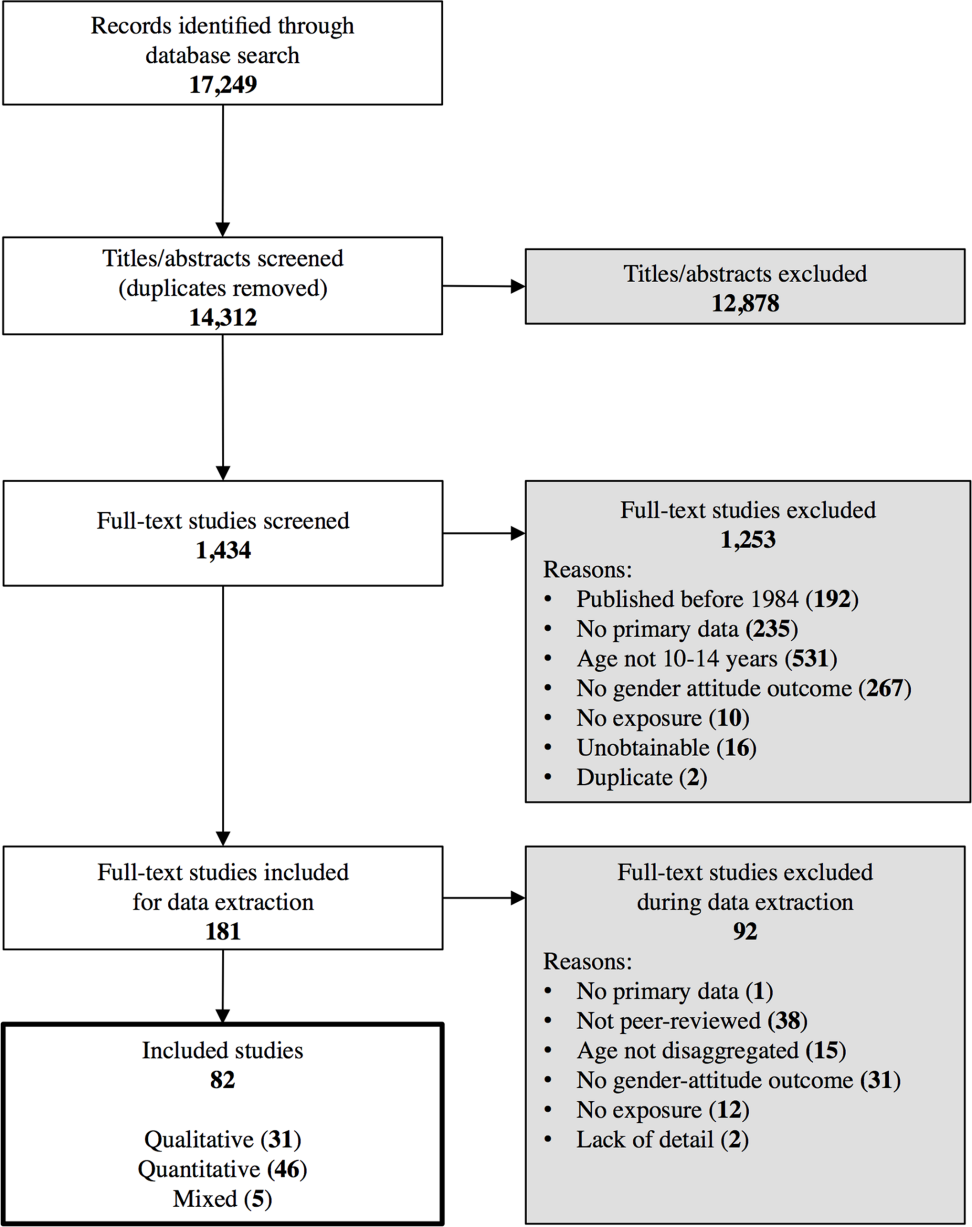
Flowchart of the screening and study inclusion process.

We screened the title and abstract of 14,312 records generated through the database searches, and reviewed the full-text of 1,434 studies. One hundred and eighty-one studies were initially retained for data extraction and 92 were further excluded during this process, mostly because they were not peer-reviewed or did not focus on personal gender attitudes as the key outcome. Ultimately, 82 studies met all inclusion criteria (Fig 1). Forty-six of the included studies were of quantitative nature and of these 30 were cross-sectional, nine were longitudinal cohorts, two used a combination of longitudinal and cross-sectional designs, four were quasi-experimental, and one was a randomized controlled trial. Thirty-one studies were qualitative: 13 applied ethnographic study, three used phenomenology or narrative research respectively, two used grounded theory and one was a case study; nine studies did not specify their design beyond “qualitative”. Five studies utilized mixed-methods, of which three were program evaluations (one quasi-experimental and two pre- posttests with control groups) and two described their designs as cross-sectional.

Following the quality assessment criteria described earlier, the quality of most studies was rated as moderate (22 quantitative, 18 qualitative, 1 mixed-methods) or low (17 quantitative, 6 qualitative, 4 mixed-methods). Fourteen studies (7 qualitative and 7 quantitative) were classified as strong quality.

The 82 included studies represented primary data from 29 countries presented in Table 1; North America (n = 51, mainly United States [US]), Europe (n = 23, mainly Great Britain), Latin America and the Caribbean (n = 6), sub-Saharan Africa (n = 5), Asia (n = 5), Middle East African Region (n = 3), and Oceania (n = 2). Five studies involved multi-country comparisons [45–49]. Most studies included both male and female participants (n = 64) and used school-based samples (n = 57). The urbanicity (e.g. urban, sub-urban, rural) and relative socioeconomic status (e.g. low, middle, high) of participants varied both within and between studies. However, many did not specify these characteristics. Studies came from a broad range of academic disciplines, including: sociology, psychology, feminist/gender studies, anthropology, public health and education.

**Table 1:** Geographical distribution of the 82 included studies.

Region and Country	Nr of studies	References
**North America**	**50**	
Canada	2	[50,51]
United States	49*	[13,45–47,52–93]
**Europe**	**23**	
Belgium	1	[48]
Bulgaria	1*	[90]
Finland	1	[94]
France	2	[95,96]
Germany	2*	[48,97]
Great Britain	10*	[47,48,98–105]
Ireland	1	[106]
Italy	1*	[90]
Netherlands	1	[107]
Spain	1	[108]
Sweden	2*	[48,109]
**Latin America and the Caribbean**	**6**	
Barbados	1	[110]
Brazil	1	[111]
Honduras	1*	[49,112]
Mexico	3*	[46,113,114]
**Sub-Saharan Africa**	**5**	
Ghana	1	[115]
Nigeria	1	[116]
Malawi	1*	[49,112]
South Africa	1	[117]
Tanzania	1*	[49,112]
**Middle East and North Africa**	**3**	
Egypt	1*	[49,112]
Israel	1	[118]
Yemen	1*	[49,112]
**Asia**	**5**	
India	2*	[119,120]
Nepal	1	[121]
Singapore	1	[122]
South Korea	1	[123]
**Oceania**		
Australia	2	[124,125]
*Multi-country comparisons:		
Italy, Bulgaria, US	1	[45]
Mexico, United States	1	[46]
Great Britain, United States	1	[47]
Belgium, Germany, Great Britain, Sweden	1	[48]
Egypt, Honduras, India, Malawi, Tanzania, Yemen	1	[49]

*These countries were part of multi-country comparisons in the studies listed at the end of the table.

A detailed summary of all included studies (author/year, study setting, objective, design, theory, sampling and sample, data collection and analysis, key findings and quality) is available in S4 Text.

### Describing gender attitudes in early adolescence

#### Measures of gender attitudes in quantitative studies

The measurement of gender attitudes varied substantially in the quantitative studies. Most studies operationalized gender attitudes through scales, indices or single-item statements asking adolescents about gender roles (e.g. attitudes about gender-stereotypic occupations, school and family roles) [52,54,55,59,62,65,66,75,77,85,86,88,89,91,97,109,121,122,126,127], stereotypical masculine and feminine behaviors and traits (e.g. stereotypes related to sports and intelligence) [45,51,63,64,68,72,73,78,87,95,96,110,118,125] or endorsement of gender inequitable norms [47,49,56,96,116,119–121]. Other measurement categories included attitudes about gender-based violence [55,58,67,108,119], evaluation of gender non-conforming behaviors [53,70,74,107,123], and perceptions of the “ideal” man or woman [46,69,81]. Due to the heterogeneity in the quantitative measures, it was not possible to compare the prevalence and range of gender attitudes across studies.

#### Themes related to expressed gender attitudes in qualitative studies

Gender attitudes expressed by young adolescents in qualitative studies conducted in different cultural settings globally were largely stereotypical or inequitable. In a number of studies boys and girls endorsed toughness and physical strength [48,56,60,61,79,80,90,92,101,103–105,111,113–115,117,128] coupled with performance and competiveness [61,79,84,90,102,105,111,113] as essential characteristics for boys [60,61,80,83,93,99,105,111,113,115,124], and emphasized that boys should not “act like girls” or in anyway display traits typically associated with femininity (e.g. by showing emotions or physical weakness). In the US, Great Britain and Brazil, attitudes about masculinity were closely intertwined with heterosexual prowess and had strong homophobic overtones, and in many studies boys perceived male sexual drive to be biologically determined and highlighted the need to demonstrate manhood by having sex with (many) girls [48,50,71,79,83,101,103,111,114,115,117] and to exercise control over girls in relationships [56,103,115,117,128]. In studies from Brazil and Mexico, young adolescents underscored that boys and men should be able to protect and provide for their families (e.g. economically) [111,113,114].

Conversely, in many studies boys and girls endorsed femininity in terms of girls’ physical characteristics of beauty and attractiveness [50,57,71,84,98,100,103] and behavioral compliance and propriety [50,57,60,84,95,98,100,105,111,115]. Girls were described to be physically weak and vulnerable [48,60,92,100,102,105,111,115,124], to have limited freedom and mobility, and to be subordinate to male authority [48,60,82–84,92,100–102,111,112,115,117,121]. Young adolescents in several studies made explicit connections between female sexuality and promiscuity, using terms such as "sluts" or "whores” to describe girls who physically reveal “too much” or act in a sexual way [60,84,94,98–103,111,113,115,117]. In studies from the US and Great Britain, female sexuality was tied to heterosexual romance in that girls often were expected to get (and keep) a boyfriend [50,83,84,98,100,101,103,128].

While in most studies participants endorsed norms that perpetuate gender inequality, there were exceptions. In several studies boys and girls concurrently expressed both stereotypical as well as more equitable attitudes about gender norms [49,50,57,60,101,111,112,114,121]; for example, in Nepal, girls voiced strong opinions in favor of gender equality but at the same time "accepted the status quo" [121], and in Malawi boys expressed support for gender equality but simultaneously voiced their superiority to girls [49,112]. In a number of studies girls described modifying their expression of femininity depending on the social context [48,57,60,61,83,98,100–102,104]; one example being how Mexican-American girls in the US adjusted their expressions of femininity to please parents and male partners [60]. Furthermore, studies across different geographical settings described how participants (girls in particular) explicitly challenged stereotypical norms and gender inequality. In over half of all qualitative studies, a number of girls critiqued their own and older women’s restricted mobility/freedom, spoke up against men’s violence against women, rejected adult attempts to control their sexuality as well as pressures to conform to hyperfemininity norms, or noted the impossibility of living up to ideals of the "perfect girl" [46,48,50,57,60,61,82–84,92,100–102,104,115,117,121,124]. While explicit critiques of stereotypical gender norms by boys were less frequent, a few studies indicated that boys challenged masculine stereotypes about toughness and strength and expressed unease with prevailing gender inequalities between men and women [50,90,93,100,101,114,115,117,121,129].

### Factors that shape young adolescents’ gender attitudes

In the next section we summarize the key individual, interpersonal and community/ societal factors that emerged as potential influences on young adolescents’ gender attitudes based on the quantitative and qualitative syntheses. By comparing the quantitative and qualitative themes, we were able to explore how these findings confirmed, explained or contradicted each other.

We found that the quantitative studies largely explored variations in gender attitudes across individual sociodemographic characteristics (e.g. biological sex, race or ethnicity) and a limited number of interpersonal relationship and community-level factors. Table 2 presents an overview of the factors explored by quantitative studies, and Table 3 presents a detailed summary of the quantitative findings, including the number and quality of the studies underlying each theme.

**Table 2:** Thematic summary of quantitative studies.

Variable	Nr of Studies	% of Quant. Studies	% Strong/ Moderate Quality ^a^	Sign. Find.^b^	Mix Find.^b^	No Sign. Find.^b^	References
**Individual level**							
Biological sex	37	73%	65%	20	13	4	[45–47,51–53,55,58,59,63–70,73,75,78,81,85–89,91,95,96,107,109,110,116,118,121–123,125]
Ethnicity/race/immigration	5	12%	83%	3	1	1	[55,58,69,73,107]
Country comparisons	4	8%	50%	3	1	0	[45–47,110]
Age (within subject)	3	6%	100%	1	2	0	[59,65,77]
Pubertal development	2	4%	100%	1	0	1	[65,81]
Gender identity and traits	4	10%	50%	2	1	0	[65,73,74,89]
Romantic/sexual behavior	2	4%	100%	0	2	0	[63,108]
**Interpersonal level**							
Parental education/work (proxy for SES)	8	16%	75%	4	4	0	[51,63,81,86,89,91,97,109]
Parental attitudes	12	24%	58%	9	3	0	[52,54,59,62,72,74,75,81,86,97,107,109]
Parental gender roles	2	4%	100%	0	0	2	[52,109]
Sibling characteristics	5	10%	100%	0	5	0	[59,75–77,109]
Domestic violence	1	2%	100%	1	0	0	[119]
Peer pressure	1	2%	100%	0	0	1	[107]
**Community/societal level**							
School performance	4	9%	67%	1	2	0	[76,89,91,122]
Sexuality Education	3	6%	0%	3	0	0	[66,116,127]
Media	3	6%	100%	1	2	0	[56,58,89]

^a^ Quality of studies refers to the proportion of studies exploring a particular variable that was rated as strong or moderate quality. For example, 65% of studies exploring differences in gender attitudes by biological sex were rated as strong/moderate quality.

^b^ Number of primary studies that found a significant, mixed or no association with regards to the relationship between gender attitudes and the variable of interest.

**Table 3:** Summary of quantitative themes around factors associated with young adolescents’ gender attitudes.

Variable	Main Theme/Finding	Nr Studies Supporting Theme ^a^	Quality of Studies	Examples of Study Findings	Contributing Studies
**Individual level**					
Sex	Gender attitudes vary by biological sex: girls more commonly than boys report equitable gender attitudes (or boys more commonly report inequitable attitudes)	20 of 37 that explored variable	4 strong, 10 moderate, 6 low	- US, strong quality: Girls had higher approval of equitable gender roles than boys [65].	[47,52,53,55,59,65,68,70,75,81,85–89,91,107,109,118,123]
				- Netherlands, moderate quality: Boys were more likely than girls to express more negative attitudes towards gender non-conforming behavior and feel greater pressure from parents to conform to gender norms [107].	
Ethnicity, Race and Immigration History	Gender attitudes appear to vary by race, ethnic and immigration history, but there is no clear trend in associations	4 of 6 that explored variable	1 strong, 3 moderate	- Netherlands, moderate quality: Children with “non-Western” backgrounds had more negative attitudes towards gay/lesbians, and gender non-conforming behaviors; and perceived higher parental pressure to conform to gender roles (especially boys) [107]. US, strong quality: African American participants chose larger ideal male and female figures than white participants in the United States [69].	[58,69,73,107]
Country comparisons	Gender attitudes appear to vary between countries, but there is no clear trend in associations	3 of 4 that explored variable	1 strong, 2 weak	- US and Scotland, strong quality: US participants held more traditional views towards male role norms compared to Scottish participants [47].	[45,47,110]
Age (within subjects)	Looking at longitudinal studies, gender attitudes appear to change as young adolescents age, but the patterns vary by subgroup.	3 of 3 that explored variable	2 strong, 1 moderate	- US, strong quality: From the 6th to 8th grade, girls increasingly approved of gender equality while boys became less approving [65].	[59,65,77]
				- US, strong quality: Gender attitudes became less traditional between ages 7–13 and remained stable from ages 13–15. Patterns for some subgroups varied, for example by parental gender attitudes and sibling characteristics [59].	
Pubertal status	Explored by two studies; no main theme identified.	N/A	1 strong, 1 moderate	- US, strong quality: Pubertal status was not associated with gender role attitudes [65].	[65,81]
				- US, moderate quality: Paternal assessment of sons’ pubertal age was associated with sex role attitudes such that more physically mature boys had less traditional attitudes [81].	
Gendered traits and activities	Some indication of an association between gendered traits and attitudes. Three US-based studies showed that feminine traits and activities were associated with less stereotypical gender attitudes among boys, but associations among girls were not consistent.	3 of 5 that explored variable	1 strong, 2 moderate	- US, strong quality: Boys who described themselves as more feminine had more equitable gender role attitudes, while for girls no correlation was found [65].	[65,73,89]
				- US, moderate quality: Boys’ endorsement of feminine traits for self at baseline was associated with lower levels of sex typing of others at follow-up. For girls, interest in masculine-oriented activities was associated with less stereotypical attitudes towards others [73].	
Romantic/sexual behavior	Explored by two studies; no main theme identified.	N/A	1 strong, 1 moderate	- Spain, strong quality: Intimate relationship experience predicted stronger endorsement of sexism among boys; results were mixed among girls [108].	[63,108]
				- US, moderate quality: Sexual behavior was predictive of female stereotyping among girls but not boys. This relationship varied by race; sexual initiation was associated with an increase in stereotypical attitudes among African American but not White girls [63].	
**Interpersonal level**					
Parental attitudes	Gender attitudes appear to be linked to mothers and/or father's gender-related attitudes and pressures. The nature of this association varies by biological sex.	9 of 11 that explored variable	1 strong, 4 moderate, 4 low	- Sweden, strong quality: Parents’ gender equality attitudes were associated with girls, but not boys, own gender attitudes [109].	[52,54,59,62,72,74,75,77,81,86,97,107,109,128]
				- US, strong quality: Traditional gender attitudes were associated with traditional parental attitudes. Both boys and girls with traditional parents displayed little change in attitudes over time [59]. US, moderate quality: For girls, mother's (not fathers) attitude towards male role was strongest predictor of reduced stereotypical attitudes [52].	
Parental education and work status (indirect measure of SES)	Gender attitudes appear to be associated with parental education level (especially mothers') and parental work status.	8 of 8 that explored variable	2 strong, 4 moderate, 2 low	- US, strong quality: For boys, mother's education and employment predicted gender stereotyping; stereotypes decreased with education and increased with employment [63].	[51,63,81,86,89,91,97,109]
				- Sweden, strong quality: Higher maternal education was associated with boys and girls perceiving gender equality in the home as very important. For girls, this was also related to mother working full-time outside of the household [109].	
				- Germany, moderate quality: Higher SES families expressed more equitable gender role orientations [97].	
Parental gender roles in the home	No evidence that parental gender roles in the home are associated with child's gender attitudes.	2 of 2 that explored variable	1 strong, 1 moderate	- Sweden, strong quality: There was no relationship between mother's time doing housework and gender attitudes [109].	[52,109]
				- US, moderate quality: Sex role attitudes were not associated with fathers’ participation in household work [52].	
Sibling characteristics (sex, birth order and gender attitude of sibling)	Gender attitudes appear to be associated with sibling dyad composition (age, sex, attitudes of siblings); associations vary by sex.	5 of 5 that explored variable	2 strong, 3 moderate	- US, moderate quality: Having a younger brother was associated with stereotypical gender attitudes among firstborn girls. Sibling attitudes negatively predicted stereotypical attitudes over time [77].	[59,75–77,109]
				- Sweden, strong quality: First- and second-born siblings had different gender attitude patterns. Firstborn boys were more stereotypical if they grew up with a male sibling. For girls the same was true, but with older brothers. In contrast, stereotypical attitudes among second-borns with older brothers declined over time [109].	
Domestic violence	Explored by one study; no main theme identified.	N/A	Moderate	- India, moderate quality: Boys were more likely to condone violence against girls if they witnessed inter-parental violence, and were victims of violence in the home [119].	[119]
Perceived peer pressure	Explored by one study; no main theme identified.	N/A	Moderate	- US, moderate quality: No association between peer pressure to conform to gender norms and reported gender attitudes [107].	[107]
**Community/Societal level**					
Academic success/ performance	Limited evidence that higher academic achievement is associated with more liberal gender attitudes.	3 of 3 that explored variable	2 low, 1 moderate	- US, moderate quality: Participants with higher reading level gave less stereotyped responses [89].	[89,91,122]
				- US, low quality: School test scores were positively correlated with equitable gender attitudes [91].	
				- Singapore, moderate quality: students at highly selective ("elite") schools held more equitable gender attitudes compared to students not in such schools [122].	
Sex education	Evidence suggests that exposure to sex education curricula is associated with more equitable gender attitudes, though studies are all quasi-experimental and of low quality.	3 of 3 that explored variable	Low	- US, low quality: Participants in a school-based sex education intervention expressed less traditional attitudes toward women [66]. Nigeria, low quality: Gender equitable attitudes increased over the course of a school year during which a Family Life and HIV Education curriculum was implemented [116].	[66,116,127]
				- U.S, low quality: Participation in the "Fair Play” curriculum was associated with less stereotypical perceptions of occupational, school, and family roles [127].	
Pornography or sexually explicit media	Some indication that viewing sexually explicit media or pornography is linked to more stereotypical gender attitudes.	2 of 3 that explored variable	1 strong, 1 moderate	- US, strong quality: Adolescents who used sexually explicit media had less progressive gender attitude. Boys were more likely than girls to use such media. In adjusted analysis, media was only predictive of gender role attitudes among girls [56].	[56,58]
				- US, moderate quality: Viewing pornography was related to rating female promiscuity and male dominance as perceived causes for rape. The more girls (but not boys) believed to have learnt from pornography, the higher the score of reported stereotypical attitudes about rape causes [58].	

^a^ Number of studies supporting the identified theme, out of all studies that explored that particular variable. For example, 37 studies explored gender attitudes differences by sex, and 20 found and that attitudes differ between boys and girls (i.e. 20 studies supported this theme).

We further used the qualitative themes to explain the variability reported in the quantitative studies, and to understand how young adolescents learn about and construct gender attitudes in relation to their social environments. Table 4 presents a summary of the analytical and descriptive themes or findings that emerged from the qualitative synthesis, and Table 5 provides a detailed summary of each descriptive theme (italicized in the text below) including illustrative quotes. Table 5 also includes an explanation of the confidence in each qualitative review theme or finding based on the CERQual assessment as: high (strong evidence), moderate (some evidence), and low (weak evidence).

**Table 4:** Thematic synthesis of qualitative studies.

Ecological Level	Analytical Theme	Descriptive Theme	Contributing Studies
Individual level	Gender attitudes differ by biological sex.	Girls, more commonly than boys, challenge gender inequalities.	[46,48,50,57,60,61,82–84,92,100–102,104,115,117,121,124]
		Boys appear to face more social barriers than girls to challenge gender inequalities.	[50,60,84,93,94,99,102,115,116,121,124]
	Gender attitudes intersect with the construction of norms about other social and cultural categories.	Gender norms intersect with race/ethnicity norms and identities.	[82,93,99,103]
		Young adolescents of immigrant background experience clashing cultural messages about gender norms.	[46,60,79,84,93,99,100]
		Social class may influence the gendered opportunities available to young adolescents.	[61,98,100,102,103,106,114]
	Pubertal onset brings new gender pressures and expectations.	With the onset of puberty, boys are expected to prove masculine toughness and sexual prowess.	[79,114,117]
		With the onset of puberty, girls are expected to deemphasize physical body changes, and are increasingly restricted.	[79,82,100,117]
Interpersonal level	Parents and other family members communicate gender norms overtly and covertly.	Young adolescents learn about gender role expectations in the home.	[60,106,111,114,115]
		Mothers appear to be especially important in teaching and enforcing stereotypical gender norms.	[60,82,84,100,111,115]
	Parents strictly control and sanction their daughters.	Tough parental control and restrictions for girls, often tied to concerns about female sexuality.	[60,82,84,92,98,100,102,106,111,115,117,121]
	Peers are central in establishing and upholding gender norms.	Male peer groups enforce competition, toughness and heterosexual prowess.	[61,79,80,84,103,104,111,113,114,117,128]
		Boys who fail to achieve local masculinity standards are bullied and ridiculed by their peers.	[48,61,71,79,80,83,84,93,101,105,111,113,114,117,124]
		Female peer groups enforce norms of beauty, appearance and heterosexual romance.	[57,61,82,84,98,99,101,104,111]
		Peers police gender boundaries related to female sexuality.	[94,98,99,101–103,113,114]
		Girls experience control and exclusion by male peers.	[49,61,92,94,99,105,112,128]
Community/Societal level	Schools communicate and uphold gender norms through rules, activities and teacher-student relationships.	School cultures, traditions and rules contribute to the upholding of gender norms.	[57,61,83,84,98,100,105,106,124]
		Schools appear to disproportionally favor boys’ activities and performances.	[61,84,90,92,93,100,105]
		Teachers reinforce stereotypical gender norms.	[60,79,80,83,90,94,98,104,105]
	Media appears to shape gender attitudes in various ways.	Different media appear to influence young adolescent’s gender attitudes.	[48,50,79,114,124]
		Gender attitudes are constructed through social media and sexting.	[103,128]

**Table 5:** Summary of qualitative themes around young adolescents’ construction and negotiation of gender attitudes.

Review Finding/Theme	Illustrative Quote	Confidence in the Evidence	Explanation of Confidence Assessment	Contributing Studies
**Individual level**				
***Girls*, *more commonly than boys*, *challenge gender inequalities*.**				
Studies across the world showed that girls commonly challenge gender stereotypes and inequalities. In studies from Nepal, Mexico and the US, girls voiced the importance of gender equality in the household, and in the US and Great Britain, girls rejected pressures to conform to “hyperfeminine” norms. Latina and African American girls in US-based studies rejected adult attempts to control their sexuality, and in a South African study girls offered strong critique of men's violence against women. Similarly, in one study based in Ghana found that girls emphasized women's rights to relationship power.	*– If I were a guy I’d be treated differently and I told [my family] you know what I don’t care because I’m a girl*. *If you wanted a guy*, *why don’t you go have another kid or something*. (Mexican-American girl, US) [60]	High confidence	18 studies of mainly strong or moderate quality and relevance. Thick and coherent data from 11 high, middle and low-income countries across different geographical regions.	[46,48,50,57,60,61,82–84,92,100–102,104,115,117,121,124]
	*– There’s not really girls’ jobs or boy’s jobs*. *You can get whatever job you really want to get*. (Girl, Australia) [124]			
***Boys appear to face more social barriers than girls to challenge gender inequalities*.**				
Findings from across the world indicated that it might be more difficult for young adolescent boys (than girls) to express gender equitable attitudes and challenge inequalities. Studies from the US, Finland, Nepal and Great Britain showed that while it is generally ok for girls to challenge gender norms, boys who do not conform to local masculinity stereotypes are socially stigmatized. In studies from Ghana, Nigeria, Australia, Canada, Great Britain and the US boys reported more freedom and opportunities than girls, indicating a lack of motivation to challenge prevailing norms that are benefitting them (as boys).	*– [If I were a girl] I wouldn’t feel as many pressures from other people from the same gender—boys are more likely to single out and make fun of those who do not fit those perceptions of “coolness”*. (Chinese-American boy, US) [84]	High confidence	11 studies of moderate quality and high relevance. Thick, coherent data across high and low-income countries in different geographical regions.	[50,60,84,93,94,99,102,115,116,121,124]
	– *Interviewer*: *Would you change things*? *Boy*: *It’s good for us innit*?! *We’re lads*! *So (…) it’s all right for us but I think I quite I feel the women would wanna change it*. (Asian boy, England) [99]			
***Gender attitudes intersect with race/ethnicity norms and identities*.**				
Young adolescents in studies from the US and Great Britain constructed racial masculinities and femininities. For example, Asian boys of Muslim origin in a study from England constructed different masculinity norms for White and Black boys, and in a US-based study Latina and African-American girls expressed different femininity ideals.	– *But then again if you do summit and they get a beating then you get the blame for it*, *even if they started it nah-jus’ can’t beat the white guy; the White guy is alright*, *the black lad’s messed up*. (Asian boy, England) [113]	Low confidence	4 studies of moderate quality and high relevance. Less coherent data limited to 2 high-income counties in Europe and North America.	[82,93,99,103]
***Young adolescents of immigrant background experience clashing cultural messages about gender norms*.**				
Studies from the US and Great Britain described how young adolescents of immigrant background experience clashing gender expectations between their country of origin and their new society. For example, studies from the US found that Chinese-American girls were in a “double-bind” when trying to meet high academic expectations from parents while displaying academic ignorance with peers at school; and that girls of Mexican-American origin were strictly controlled by parents who feared that their daughters would become "promiscuous" because of the sexual freedom in the US. In England, one study found that young Muslim boys constructed gender identities in opposition to "Western" masculinities, and Muslim immigrant girls experienced greater parental restrictions on their mobility and freedom than native girls.	– *The concept of being popular is different in China and in America*. *In America*, *a very popular girls isn’t much different from a female gangster*. *Do you want us to become female gangster*? (Chinese-American girl, US) [84]	High confidence	7 studies of mainly strong or moderate quality. Thick data from 2 high-income and 1 middle-income country, mostly coherent findings across sites although some details are context specific.	[46,60,79,84,93,99,100]
	– *Interviewer*: *I’ve noticed that a few girls here don’t wear dibuka [hijab] (…) Boy*: *You know*, *they don’t really take Islam seriously*, *like they’ve got a taste of*, *um*, *it’s like they’ve got higher status over here and they don’t really care*. (Muslim boy, England) [99]			
***Social class may influence the gendered opportunities available to young adolescents*.**				
In studies from Great Britain and Ireland, girls distinguished between working-class and upper-class femininities, for example by emphasizing the need to be a (respectable) "lady” in contrast to (unrespectable) "townie" girls. In a US-based study branded clothing was an important part of constructing middle-class femininities, while toughness generated higher social status among working-class girls. One Mexican study highlighted difficulties for boys to meet prevailing machismo norms (e.g. working to provide for the family) given their low socioeconomic status.	*– The girls told me that they had recently attended a school disco where some girls from a “less selective” school had been able to attend (…) In hushed tones*, *the girls told me that to their horror they had found a condom in the girls’ toilets and that “it could not possibly have belonged to anyone from their school” for they “would never be so common”*. (Girls at upper-class school, England) [98]	Moderate confidence	7 studies of moderate quality and relevance. Fairly thick and coherent data from 3 high-income countries and 1 middle-income country in Europe, North America and LAC.	[61,98,100,102,103,106,114]
***With the onset of puberty*, *boys are expected to prove masculine toughness and sexual prowess*.**				
For some boys in US-based studies, puberty implied an increased need to display and prove toughness and physical strength; traits that they associated with adult manliness. In a South African study, boys were taught how to be men through circumcision rites, which emphasized their readiness and biological need to have sexual intercourse. In a Mexican study boys associated pubertal development both with sexual readiness but also with increased need to provide for their families.	*– The boys made public declarations of manliness whenever they successfully used their bodies to best others*. *On average*, *there were six brash moments*, *like “I’m a man” or “I’m the man”*, *per day of observation*. (Observation of boys, US) [79]	Low confidence	2 studies of strong quality, high relevance. Fairly thick data with low coherence limited to 2 high-income and 1 middle-income country in North America and Sub-Saharan Africa.	[79,114,117]
	– *If you are not circumcised it is a bit difficult because your “non” [penis] will still be having the cap (…) but when you are circumcised it’s easy*, *it fits*, *it is sharp and it is in*. (Boy, South Africa). [117]			
***With the onset of puberty*, *girls are expected to deemphasize physical body changes*, *and are increasingly restricted***				
With the onset of puberty, some girls in studies from the US and Great Britain experienced increased restrictions in their freedom/mobility as they were expected to assume adult (mature) femininity roles. Girls in these high-income countries were overall encouraged by others to deemphasize their physical and sexual development so as not to attract and lure boys and men. So too, girls worried about increasing pressures to act like girls and no longer be able to do "boys" activities as they entered puberty.	*– I might change because my mum won’t let me play football because you’re grown up and you’re playing baby stuff and all that stuff (…) You can’t really play football because it’s just a bit weird playing with the boys…* (Girl, England) [100]	Low confidence	3 studies of moderate quality, high relevance. Moderately thick data but findings are context specific and limited to 2 high-income countries in Europe and North America.	[79,82,100,117]
**Interpersonal level**				
***Young adolescents learn about gender role expectations in the home*.**				
Studies indicated that girls and boys learn about gender roles in the home through indirect and direct messages from parents and other family members. In studies from Ireland, Ghana and Brazil, girls mentioned that they (in contrast to boys) were expected to take care of the home and younger siblings. Young adolescents in Ghana were also taught to distinguish between the status and sanctions associated with female (cooking, caretaking) and male (physical/technical) household tasks. In one US-based study, girls learnt that fathers control mothers by observing domestic violence in the home.	*– A girl stated*: *“My mom is afraid of my dad” (…) She told us a story about when her mother got a job at a restaurant*, *she was told by her husband that she was not allowed to work*. (Mexican-American girl, US) [60]	Moderate confidence	5 studies ranging from mainly moderate and strong quality and high relevance. Somewhat thick data from 5 countries of varying income levels in different geographical regions.	[60,106,111,114,115]
	*– It is the woman’s responsibility to care for the children because the man provides the money*. *When the couple is about to marry the family members state these things to them*. (Boy, Ghana) [115]			
***Mothers appear to be especially important in teaching and enforcing stereotypical gender roles*.**				
Studies from across the world described how mothers play a central role in reinforcing gender norms messages, especially to their daughters. In studies from the US and Great Britain, girls learnt from their mothers about what it means to be a woman and to stay away from men once their bodies start to change. Mothers in a Brazilian study taught their daughters to fear male sexuality and aggression, while in a study in rural Ghana girls learnt from their mothers that women should be deferent and passive in relation to men. In studies from US and Ghana, young adolescents also mentioned that they typically turn to mothers because of their fathers’ limited availability.	*– It’s really hard I can’t talk to my dad about anything*, *he either yells too much or he takes it the wrong way*. *It’s like*, *there’s these old Mexican men*, *they’re really strict about everything*. (Mexican-American girl, US) [60]	Moderate confidence	6 studies of mainly moderate or strong quality and high relevance. Fairly thick data from 4 high, middle and low-income countries in different geographical regions.	[60,82,84,100,111,115]
	– *I have learnt from my mother that when a woman is talking to a man she talks in an apologetic manner*. (Girl, Ghana) [115]			
***Tough parental control and restrictions for girls*, *often tied to concerns about female sexuality*.**				
Studies across the world described how parents employed a wide range of strategies to control daughters, including the control of a girl’s engagement in "boys’ activities”, and school choices. Control and sanctions were often related to parental attitudes and concerns about sexuality and girls' changing bodies. Girls described parents’ efforts to control or punish their relationships with boys, as well as their movement and appearance outside of the household. One study in Ghana highlighted how girls received stricter sanctions for pre-marital pregnancy, and in another study in England, Muslim boys saw themselves as “protectors” of their sisters’ sexual “respectability”, involving the control of their sisters mobility and appearance.	*– Sometimes I wish I was like my brother because my dad lets him do everything*. *It’s not because he’s older*, *because when my brother was my age my dad let him go everywhere by himself*. *And with me*, *no*. *Guys can’t get pregnant*, *guys can’t do this*, *guys can’t do that*, *but girls…* (Mexican American girl, US) [60]	High confidence	10 studies of mainly high or moderate quality and relevance. Thick and coherent data from 4 high/middle-income and 2 low-income countries in different geographical regions.	[60,82,84,92,98–100,102,106,115,117,121]
	*– She wanted to race snowmobiles and her dad would only let her if she like covered up her whole face with a mask (…)*. *So nobody saw her cause her dad didn’t think it was a sport for girls*. (Boy, US) [92]			
***Male peer groups enforce competition*, *toughness and heterosexual prowess*.**				
Studies from the US and England showed how male peers’ pressured each other to engage in various forms of physical (athletics/sports activities and fighting) and verbal challenges (banters, teasing) as a way of proving masculinity. Similarly, low-income boys in studies from Mexico and Brazil demonstrated their toughness through risk-taking practices such as heavy drug use. Across the world, male peer groups encouraged (early) sexual wooing of (many) girls as a way to display masculinity.	*– Santiago and James arm wrestle*. *James loses*. *Santiago screams to James “You’re a woman*!*” Santiago and James laugh*. *Brandon says to James*, *“Until you don’t [sic] beat him you’re a woman”*. (Observations of Mexican-American boys, US) [79]	High confidence	12 studies of mainly high or moderate quality and relevance. Thick and coherent data from 10 high and middle-income countries in different geographical regions.	[61,79,80,84,103,104,111,113,114,117,128]
***Boys who fail to achieve local masculinity standards are bullied and ridiculed by their peers*.**				
Studies from different geographic regions found that boys harassed and expressed homophobic attitudes towards peers that did not meet prevailing hegemonic heterosexual masculinity norms. Common examples included public embarrassment of physical weaknesses, homophobic commentaries. Male peers commonly used ironic humor to enforce hegemonic masculinity, for example by "jokingly" calling each other "gay", "faggots", “pussies” or “bitches”.	*– Most of the time boys will tell you that if you don’t approach a girl and tell her that you love her*, *it means you have been bitten by a mouse*, *that you are a coward*. *They say you are burning*, *you are afraid of girls (…) and the fact that you are scared of girls means there is something that you are scared of–you are scared of sex*. (Boy, South Africa) [117]	High confidence	16 studies of mainly high or moderate quality and relevance. Thick and coherent data from 10 countries of varying socioeconomic status.	[48,61,71,79,80,83,84,93,101,105,111,113,114,117,124]
***Female peers enforce norms of beauty*, *appearance and heterosexual romance*.**				
Female peer groups in studies from the US and Great Britain constructed femininity norms through repeated remarks on the importance of being a “girly” girl, defined as slim, beautiful and sexually attractive (to men). Heterosexual romance was central to the construction of femininity across both these countries as well as in a study from Brazil where girls constantly evaluated each other’s popularity with boys.	– *Frankie had been accepted into the girly girl group because of her popularity with a number of boys*. *By having a relationship with a boy*, *Frankie was fulfilling a dream that many of the girls hoped for themselves*. (Observations of girls, England) [98]	High confidence	9 studies of mainly high and moderate quality and relevance. Thick and coherent data across 2 high and 1 middle-income country in Europe, North and South America.	[57,61,82,84,98,99,101,104,111]
**Peers police gender boundaries related to female sexuality.**				
Girls in studies from Great Britain, Finland and Mexico mentioned experiences of peer harassment and shaming/policing related to their appearance and sexuality. For example, girls regarded as “too” sexual (‘bad girls’) were shamed through “slut-calling” and sexist comments by both male and female peers. In the Mexican study, female peers also stigmatized and teased girls that they perceived to be (or act) homosexual.	*Girl*: *“I think a bit of tart is alright… I mean you want to look good*.*” Friend*: *“Yes*, *you want to look good… but no tart is never alright”* (Girls, England) [98]	Moderate confidence	8 studies of mainly moderate quality and relevance. Fairly thick data from 2 high and 1 middle-income countries in Europe and LAC.	[94,98,99,101–103,113,114]
	– *I felt comfortable just wearing T-shirt… and boys [would] tell me*, *“Why are you coming to school like that*?*”* (Girl, US) [82]			
**Girls experience control and exclusion by male peers.**				
Studies from studies across the world indicated that young adolescent boys exercised control over female peers in various ways. Girls in the US, Great Britain Honduras and Egypt described how boys hindered their participation in physical activities such as soccer games. In Finland, some girls mentioned how male peers prevented them from voicing their opinion at school.	*– They say we’re not for playing football because we’re girls and we make so many mistakes because we are weak*. (Girl, Honduras) [112]	High confidence	7 studies of ranging from low to high quality. Fairly thick and coherent data from 10 high and low-income countries in different geographical regions.	[49,61,92,94,105,112,128]
	– *Say a girl was really*, *really good at football*, *and she was showing them up (…) then [the boys] might not like her… Yeah*, *‘cause if they were better*, *if they were worse*, *they’d tease them*. (Group of Chinese-American boys, US) [92]			
**Community/Societal level**				
***School cultures*, *traditions and rules contribute to the upholding of gender norms*.**				
Studies from the US, Great Britain, Ireland and Australia indicated that schools are important institutions when it comes to both regulating and upholding gender norms through different traditions and cultures. Examples included the enforcement of school uniforms, policing of girls clothing and the attribution of higher status to boy’s activities and performance.	*– I got to the gates and I was called back by Mrs*. *Fairhead*. *She asked me why I thought I could go home in my netball skirt and said that I shouldn’t do this… I should not be seen to be dressed like this by people from outside of the school*, *I could run into builders and perverts on the way home and everything*! (Girl, England) [98]	Moderate confidence	9 studies of moderate quality and relevance. Fairly thick and coherent data across 4 high-income countries in Europe, North America and Oceania.	[57,61,83,84,98,100,105,106,124]
***Schools appear to disproportionally favor boys’ activities and performance*.**				
In studies from the US and Great Britain, school sports and physical activities promoted stereotypical masculinity norms and the pressure on boys to participate and perform well in sports such as football was strong. For boys in a study from Great Britain, football provided an opportunity to “practice “being (adult) men, and in the US boys who did not perform well in physical activities were marginalized. In a US-based study, “feminine” activities such as cheerleading emphasized attractiveness rather than physical performance.	*– The teacher gets mad at me for forgetting my (gym) clothes and calls me a liar when I say I don’t feel good*. *This happens in front of my class-mates*, *and (…) I don’t want them to know I’m trying to get out of it*, *because most boys like sports*. (Boy, US) [90]	Moderate confidence	7 studies of moderate quality and high relevance. Somewhat thick and coherent data across two high-income countries in Europe and North America.	[61,84,90,92,93,100,105]
***Teachers reinforce stereotypical gender norms*.**				
According to studies conducted in Western Europe and the US, teachers enforced femininity norms at schools in various ways. Examples included teachers socializing girls to act ‘lady like’ at an upper-class school in England, teachers giving less attention to girls compared to boys, undermining girls abilities and strength, or ridiculing boys that lack typically masculine characteristics.	*– During a lesson when some of the boys were continually commenting on the behavior of some of their female classmates*, *the teacher joked*, *“This is how the strong Finnish women develop*: *they survive by being teased*!*”* [94](Finland)	High confidence	9 studies of mainly high or moderate quality, and high relevance. Fairly thick data across 3 high-income countries in Europe and North America.	[60,79,80,83,90,94,98,104,105]
	*– The guy teachers are*, *I guess they’re not girls so they tend to think that stuff’s harder for girls than guys like at sports*, *but it isn’t true*. (Mexican-American girl, US) [60]			
***Different media appear to influence young adolescent’s gender attitudes*.**				
Young adolescents in studies from high-income countries described how various media (TV, popular culture, comic books, music, video games and advertisements) influenced their perceptions of masculinities and femininities. Examples included stereotypical TV portrayals of men as perpetrators of violence and women as victims, the admiration of rappers from violent neighborhoods, communication of sexual and gender norms through media campaigns and heteronormative portrayals in comic books.	*– I don’t know we always hear about it on the news when the man pressures the woman into doing something*. *But we never really hear it that the woman’s doing something to the man*. (Girl, England) [48]	Low confidence	5 studies of moderate quality and relevance. Thin and context specific data from 8 countries in high-income settings (Europe, North America, Oceania).	[48,50,79,114,124]
**Gender attitudes are constructed through social media and sexing**				
In Great Britain, one study found that exchanges of sexual pictures were important strategies for boys to display masculinity, and for girls to prove femininity (attractiveness) while at the same time not appearing too slutty. A study in France found that online social networks presented opportunities for boys and girls to explore alternate gender identities and norms by pretending to be someone else online.	– *Interviewer*: *How does like all this sending of pictures and stuff relate to like having sex and doing stuff*? *Boy*: *because if a girl sends a picture to you it means that she probably wants to meet up with you and stuff*. (Boy, Wales) [103].	Low confidence	2 studies of moderate or low quality, and moderate relevance. Relatively thin data from two high-income countries in Europe.	[103,128]

### Individual-level factors.

#### Sex

Over half of quantitative studies (N = 37) compared gender attitudes by biological sex and 20 found that adolescent boys are more likely than girls to endorse norms that perpetuate gender inequalities, or conversely that girls report more equitable gender attitudes [47,52,53,55,59,65,68,70,75,81,85–89,91,107,109,118,123] (Table 3).

This was consistent with the strong qualitative review finding that *girls*, *more commonly than boys*, *challenge gender inequalities* [46,48,50,57,60,61,82–84,92,100–102,104,115,117,121,124] (Table 5). Qualitative themes of strong confidence further showed that these differences might be partly explained by how *boys face more social barriers to challenge gender inequalities than girls*. In studies from the US, UK, Nepal and Finland boys described that “it is harder to be a guy” because of the social stigmatization and ridicule of boys that do not conform to the stereotypical masculine norms [60,84,94,99,121]. At the same time, boys from studies conducted in Ghana, Nigeria, Australia, Canada, Great Britain and the US consistently reported having greater relative freedom and power compared to girls, and at times less motivation to challenge gender stereotypes and inequalities [50,60,93,99,111,115,116,124].

#### Ethnicity, race and immigration history

Three quantitative studies conducted in the US found differences in gender attitudes by race, ethnicity and immigration history [58,69,73]. Due to the heterogeneity of study populations and measures there was however no clear overall trend in these associations. Similarly, three in four studies exploring cross-country variations found that gender attitudes vary between countries (e.g. one study found that attitudes towards male role norms were more stereotypical in the US compared to Scotland) [47], but without a clear trend in associations [45,47,110]. Two studies conducted outside of the US found that language spoken at home and parents’ immigration history was associated with varying gender attitudes [107,122]. For example, adolescents of immigrant background in the Netherlands reported more negative attitudes towards gender non-conforming behaviors and perceived higher parental pressures to conform to stereotypical gender roles compared to their native-born peers [107].

Qualitative review findings of strong confidence showed that such variations in part may reflect how *young adolescents of immigrant background experience clashing cultural messages about gender norms*, stemming from differences between their families and host communities [46,60,79,84,93,99,100]. As one example, studies from England [99] and US [60] described how immigrant Asian and Mexican American girls, respectively, experienced greater parental restriction compared to native peers. Such restrictions were often related to family attitudes about sexuality and concerns that girls’ changing bodies would attract male attention. While of low confidence, another qualitative review theme highlighted how *gender norms intersect with racial/ethnical norms and identities*: for example, studies from Great Britain and US described how boys constructed “black” versus “white” masculinities, while Latina and African-American girls in the US expressed different femininity ideals [82,93,99,103].

#### Social class

In several quantitative studies (N = 8) adolescents from higher income backgrounds and/or with more highly educated parents generally expressed more equitable gender attitudes [51,63,81,86,89,91,97,109]. For example, five studies conducted in North America and Western Europe found that higher maternal education and maternal employment were associated with less stereotypical gender attitudes [51,63,81,91,109].

As a potential explanation for these findings, qualitative themes of moderate confidence indicated that *social class might influence the opportunities available to young adolescents*, which in turn may shape their gender attitudes [61,98,100,102,103,106,114]. For example, in studies from the US, Great Britain and Ireland, girls conformed with feminine norms specific to their social class. This included acting like “ladies” [98] or wearing branded clothing [61] to signal higher social class, or alternatively dressing in ways that represented their toughness as working-class girls. A study from Ireland described how working-class girls, in contrast to middle-class peers had to babysit siblings rather than focus on school work [106]. In a study from Mexico, boys living on the streets described difficulties in meeting “machismo” norms (e.g. holding a job and providing for their families) because of their low socioeconomic status [114].

#### Age and pubertal development

Several (n = 7) longitudinal quantitative studies confirmed that gender attitudes change over time during the early adolescent period [59,65,81,130]. In three studies (two of which use the same dataset), gender attitudes were found to become less stereotypical with increasing age. However, these trajectories varied by sex and family context [59,65,130]. Only two quantitative studies published in 1990 examined the specific association between transition into puberty with gender attitudes, and the findings were inconclusive [65,81].

Qualitative studies from Europe, North America and sub-Saharan Africa suggested that the onset of puberty intensifies social expectations related to gender. While of low confidence, themes drawing on these study findings highlighted *that boys are expected to prove their masculine toughness and sexual prowess* [79,114,117] whereas girls are expected to *hide their developing body and are increasingly restricted*, *as parents and others in girls’ social environments* monitor and limit their mobility and freedom as ways to protect their bodies [79,82,100,117].

### Interpersonal relationship level

#### Family members

The findings from quantitative studies were mixed in terms of the role of parental influence. Two studies found that parental gender division of roles in the home (e.g., mother’s time doing house work in relation to father’s time) were not associated with their children’s gender attitudes [52,109]. In 9 out of 12 studies that examined parents’ own gender attitudes, parental attitudes were associated with children’s attitudes although the nature of associations varied by biological sex of both children and parents [52,54,59,62,72,74,75,77,81,86,97,107,109,128]. For example in a study from Sweden, parents’ endorsement of gender equality was associated with more equitable attitudes among girls, but not among boys [109]. In a US study, girls whose mothers (but not fathers) endorsed more equitable gender roles developed less stereotypical attitudes over time [52]. Most studies exploring the influence of parents focused on two-parent households, and there were not enough studies focusing on single mothers or fathers to assess whether family structure might influence gender attitudes.

Five quantitative studies (four which used the same dataset) conducted in the US and Western Europe explored the influence of sibling characteristics, including sibling attitudes, sex, and birth order [59,75–77,109]. While all of these studies found significant associations between sibling characteristics and gender attitudes, there was no clear pattern across studies and associations varied by sex.

Qualitative review findings of moderate confidence indicated that *young adolescents learn about gender role expectations in the home* through indirect and direct communication with parents and other family members, highlighting differential gender socialization processes for boys and girls. Girls from studies conducted in low, middle and high-income countries described parental expectations to assist with housework and looking after younger siblings, and contrasted their experiences to how boys were generally “let off the hook” [60,106,111,114,115]. For example, in one study from Ghana, girls had learned to value “feminine” tasks (e.g. caretaking and cooking) less than “masculine” tasks (e.g. physical and technical tasks), and noted the parental sanctions that would follow if they did not adhere to norms related to the gendered division of labor [115].

Findings from qualitative studies across the world provided strong evidence of *tough parental control and restrictions for girls* including strict supervision and regulation of their activities, appearance, education and mobility [60,82,84,92,98,100,102,106,115,117,121]. So too, there was some qualitative evidence that *mothers appear to be especially important in teaching and enforcing stereotypical gender roles* [60,82,84,100,111,115], especially for daughters. In studies from Ghana, Brazil and the US, girls described how their mothers would warn them to “stay away from men” and not engage in “immoral” behavior [60,82,98,115]. In some sites, such admonitions were linked to the father’s physical or emotional absence [60,82,115]. Similar to the quantitative studies, however, there were not enough studies focusing on family structure to compare the influence of single *vs*. two parent households or generate a more nuanced understanding of the influence of parental engagement and involvement. There were also examples of how girls learned about unequal gender power relations by observing their parents. In one US-based study, Latina girls described being aware of men’s control and power over women based on the experiences of witnessing gender-based violence in their homes [60].

#### Peers

Qualitative review findings provided strong evidence of the central roles of peers in shaping young adolescents’ gender attitudes. A theme that emerged for boys was that *male peer groups enforce competition*, *toughness and heterosexual prowess*. Specifically, male peers encouraged conformity with masculine norms by issuing physical and verbal challenges to each other [61,79,80,84,103,104,117,128], or encouraging risk-taking practices such as drug-use [113,114] and unsafe sexual practices [111]. In studies from Mexico and Brazil, gang cultures conferred higher status to long-term, older and physically stronger members of the gang, who conformed to violent and dominating masculine norms [111,113,114]. Studies from different geographical settings highlighted how male peers challenged each other to demonstrate their manhood by showing interest in heterosexual activities or through actual wooing or sexual conquest of (many) girls [61,79,80,84,103,104,111,113,114,117,128]. Qualitative themes of high confidence further showed that *boys who fail to achieve local masculinity standards are bullied or ridiculed by their peers*. Studies in this review described how boys who showed signs of physical and emotional weakness, acted in other stereotypically “feminine” ways, or failed to display heterosexual prowess were frequent targets of ridicule, including homophobic insults from their peers [48,61,71,79,80,83,84,93,101,105,111,113,114,117,124].

For girls, there was strong qualitative evidence that *female peers enforce norms of beauty*, *appearance and heterosexual romance*. Studies from the US and Great Britain showed that girls’ bodies and appearance were subjected to repeated remarks and evaluation against certain standards of beauty [57,61,82,84,98,99,104]. In these two settings as well as in studies from Brazil, girls evaluated each other’s femininity in terms of their ability to attract boys [84,98,101,111]. In all of these studies girls described the pressure to conform to norms that required them to present themselves as heterosexually attractive and to strike a balance between being “sexy” but not “too sexy” in order to maintain respectability. Based on studies from Finland, Mexico and Great Britain, there is also some evidence that *peers police gender boundaries related to female sexuality*, for example by calling girls who do not conform to norms of female respectability “sluts” or otherwise shaming or sexually harassing them [94,98,99,101–103,113,114].

Another theme highlighted how *girls experience control and exclusion by male peers*: across different settings, boys prevented girls from voicing their opinions or excluded them from “masculine” activities (e.g. soccer) in order to maintain the inequitable gender norms that gave higher status to boys over girls [49,61,92,94,99,105,112,128].

### Community/Societal level.

#### School

While three quantitative studies explored the relationship between school achievement and gender attitudes, their low quality precluded any conclusions across studies [89,91,122]. An additional three quasi-experimental quantitative and mixed-methods studies from the US and Nigeria explored the role of school-based sex education curriculums in shaping gender attitudes [66,116,127]. All found an association between exposure to sex education and more equitable gender attitudes.

Qualitative studies, exclusively from high-income countries in North America, Europe and Oceania, provided some evidence for how *schools regulate and uphold gender norms* through various rules and traditions [57,61,83,84,98,100,105,106,124]. For example, studies from England and Ireland [98,100,106] showed that in upper class schools, rules commonly related to girls’ clothing and expectations of “ladylike behavior” reinforced stereotypical feminine norms. Such norms were enforced by school authorities who, for example, regularly checked school uniforms so that skirts could be adjusted to “appropriate lengths” [98] even though the uniforms “restricted the movement” of girls [100].

There was also some qualitative evidence that *schools appear to disproportionately favor boys’ activities and performance over girls’* [61,100,105,124]. Studies from US and England described how physical education was dominated by sports such as football, which promoted toughness, aggression and competitiveness [61,84,90,92,93,100,105]. Such gender norms were communicated by teachers and classmates both through the active exclusion of girls from such sports, and through the stigmatization of boys who did not conform to masculinity stereotypes. The qualitative themes further provided strong evidence that *teachers reinforce stereotypical gender norms* [60,79,80,83,90,94,98,104,105], for example by directing attention to athletic and competitive boys [100,105] and by condoning boys’ teasing of girls as evidence of heterosexual attraction [94].

#### Media

Three quantitative studies examined the influence of media on gender attitudes, and of these two studies from the US found that viewing sexually explicit media or pornography was linked to more stereotypical gender attitudes for girls but not for boys [56,58].

However, in the qualitative studies no particular form of media emerged over others as more influential. Rather, findings provided weak evidence for how a range of *different media appear to influence young adolescent’s gender attitudes* (including the role of music and artists such as rappers [79], TV series [48], media campaigns [124] and depictions of romantic relationships in comic books [50]) and that *gender attitudes are constructed through social media and “sexting”* (exchange of sexually explicit pictures or other media) [103,128].

## Discussion

This systematic review brings together 30 years of research across 29 countries on the socio-ecological factors that influence gender attitudes during transitions into adolescence. Our findings provide a diverse evidence base for how individual and interpersonal relationship factors, in particular, shape the gender attitudes of boys and girls.

However, the review also highlights the paucity of data on this topic from LMICs, even though 90% of the world’s adolescents live in these countries [2]. Rather, nearly all of the peer-reviewed studies included in the current review were conducted in North America or Western Europe, leaving a large information gap from the global South.

Nevertheless, when we look across the review findings globally, there is some uniformity in study findings. First, the qualitative studies suggest that even in the early adolescence phase, boys and girls in different cultural settings commonly endorse norms that perpetuate gender inequalities. For example, studies highlighted how young adolescents’ attitudes supported masculinity predicated on toughness/competitiveness and heterosexual prowess, in contradistinction to femininity predicated on weakness, physical appearance and the control and shaming of female sexuality. Encouragingly, some studies also report ambivalence and challenging of stereotypical gender norms by many young adolescents, suggesting that gender attitudes among this age group are amenable to change.

Secondly, boys and girls appear to differ in their endorsement of stereotypical gender norms and qualitative review findings suggest that this sex-based difference may be due to the differential gender socialization processes and pressures. Gender attitudes of girls seem to be shaped by how parents, siblings, peers and teachers overtly and covertly police their appearances and sexualities, and restrict their mobility and freedom. Whether or not they try to challenge stereotypical gender norms, girls appear to experience comparatively limited freedom and recognize their own disadvantage *because they are girls*. Nevertheless, girls appear less likely than boys to accept stereotypical or inequitable gender norms. This is in line with increased recognition that adolescent girls are not passive victims of gender discriminatory practices; and that enhancing and supporting their individual agency and autonomy can help promote equitable gender norms and attitudes [36].

In contrast, we found few examples of boys challenging norms that perpetuate gender inequalities. The review findings indicate that gender attitudes of boys are strongly shaped by peer sanctions reinforcing stereotypically masculine attributes and behaviors (discussed in more detail below). As noted by Lahelma [94], whose observations of Finish middle-school students was included in the present review, “boys experience restrictions not from being boys, but because they are the *wrong sort of boys”*. Challenging stereotypical norms or engaging in stereotypically feminine activities (e.g. household chores) may be associated with perceived loss of male status and power [36,131]. Additionally, boys’ reluctance to challenge norms may be explained by the lack of role models who demonstrate non-stereotypical and alternative masculinity norms and practices [36]. For example, Barker’s [132] research with older adolescent boys and young men in Brazil, India and Nigeria, found that boys whose viewpoints were “supported or reinforced by someone else in their social context” were more likely to endorse equitable norms. As explained by Bicchieri [133], personal attitudes are strongly correlated with perceived peer social norms; i.e., my perceptions of what my peers endorse and do, and what my peers expects of me, will in turn influence my own attitudes and behavior.

Thirdly, gender attitudes appear to vary by ethnicity, race, immigration history and social class. For example, several studies highlighted that masculinity norms of minority groups may differ from those of the dominant culture. This reflects the intersectionality of social, cultural, and economic factors as they influence and shape multiple types of social norms and inequalities including gender norms [134,135].

Finally, this review reinforces the centrality of parents and caregivers in shaping gender attitudes of young adolescents. This is consistent with studies focused on early childhood, which suggest that gender socialization begins at birth and operates throughout childhood at multiple levels through play and interactions with parents and other family members [136]. However, while there were several themes around the central role of parents, few studies focused on the role of family structure or how parental involvement as well as work in- or outside of the household might influence gender attitudes. More in-depth research around how family context shapes gender socialization processes in early adolescence is thus needed. For example, more evidence is needed around the effectiveness of interventions that focus on enhancing parental skills and improve parent-child relationships, which can be promising approaches for improving early adolescent health and development [137].

Socialization processes during early adolescence differ from early childhood, as the entrance into puberty brings new expectations and roles. In the current review, peers emerged as one of the strongest influences on personal gender attitudes among 10–14 year olds. This is consistent with research suggesting that the influence of peers is greater during early adolescence than either before or after [138,139].

While there is some research highlighting that schools and teachers in particular are important institutions for gender socialization during childhood and adolescence [140], the specific influence of these factors on young adolescents’ gender attitudes remains poorly studied outside of high-income settings. Studies show that when girls remain in schools, gender equality is enhanced by delaying marriage and reducing early childbearing [30]. However, drawing on limited qualitative studies from high-income countries, our review suggests that schools can also reinforce stereotypical gender norms by promoting male dominated sports in physical education that reinforce toughness and competition, and excluding girls and boys who do not conform to such values. This finding is consistent with other literature highlighting that prevailing school cultures may reinforce traditional and conservative values about gender norms [141].

Another societal level institution that is hypothesized to play a role in shaping gender attitudes is both receptive (e.g., TV, radio) and social media. Given the small number of studies that have explored this issue and the rapid changes in media consumption habits of young people, additional research is needed to better understand the influence of media on gender attitudes of young adolescents.

### Strengths and limitations

While many of the findings of this review may not come as a surprise to those familiar with adolescent health and gender socialization research, to our knowledge this is the first attempt to systematically bring together the evidence on what shapes young adolescents’ gender attitudes globally. This review highlights what is currently known about gender attitudes in early adolescence as well as where research in this field is currently lacking. The strength of the review is the use of a rigorous systematic approach and its grounding in peer reviewed primary studies across different geographical and cultural settings, the use of the widely applied social-ecological framework to analyze and synthesize the findings, and the assessment of the robustness of the review findings/themes. The review also highlights the importance of reviewing both qualitative and quantitative research to understand the complexity that lies behind the formation and expression of gender attitudes.

The results from the current review should also be considered in light of its limitations. While we strived for a comprehensive search of the literature, it is possible that we missed relevant studies. For example, we were unable to review grey literature published on websites and in reports that may have captured more studies from LMICs. Because of this restriction, all results should be interpreted with caution when applied to settings other than North America and Western Europe. However, we attempted to capture as many relevant studies as possible by searching a wide range of databases and by piloting our search strategy, while also maintaining the quality of research that is supported by the peer review process. Furthermore, with few exceptions most studies reported in this review were based on cross-sectional study design, limiting the ability to draw temporal conclusions on factors that influence gender attitudes over time. Even though the number of longitudinal studies on whether and how gender attitudes change during this period of life was limited, there is some indication that early adolescence may be a turning point for the development of gender attitudes. For example, research by Crouter et al. [59] indicates that on average, prior to age 14 adolescents expressed more equitable gender attitudes as they aged, while after age 14 gender attitudes became increasingly more stereotypic.

Furthermore, the vastly different outcome measures used in quantitative studies limits their generalizability. It is also difficult to draw conclusions about how individual, interpersonal and community level factors influence the construction of gender attitudes at a global level, as their influence is context specific (both between countries and within countries) including to subpopulations.

### Implications for programs

While it is beyond the scope of the current review to determine what interventions and approaches can change inequitable gender attitudes, the review findings point to a few areas that should be considered in developing programs during transitions into adolescence.

The findings highlight that programs to promote gender equality and tackle harmful stereotypical attitudes need to be tailored to the specific needs and influences of boys and girls. Approaches to empower girls to overcome the restrictions and disadvantages they face may include improved access to education (completion of primary and secondary school) and informing girls about their rights, as well as other activities (e.g. mentoring, sports) that have been found to promote girls’ agency, autonomy, self-esteem and ability to challenge inequitable gender norms [36,142]. On the other hand, boys need approaches that both enable them to recognize their unearned privileges and power while supporting them to challenge stereotypical norms about masculinities and femininities, and rewarding rather than stigmatizing them when they are able to do so. Our findings also highlight the importance of recognizing the diversity of the early adolescent population by tailoring approaches to specific sub-cultures of boys and girls based on factors such as race and ethnicity, immigration history and social class.

The importance of puberty as a key moment for shaping gender attitudes, and the close links of such attitudes to sexuality suggest that comprehensive sexuality education and other developmentally appropriate life-skills education curricula are key entry points. There is increasing evidence that young adolescents benefit from comprehensive sexuality education that helps empower them to make informed decisions and be equal partners in relationships [137,143,144]. Given the evidence that addressing gender and power relations as part of comprehensive sexuality education programs is related to better sexual and reproductive health outcomes [145], it is essential that such programs include explicit content on gender norms, beginning in childhood and continuing throughout adolescence.

Importantly, the strong influence of interpersonal relationships on young adolescents’ gender attitudes supports previous research highlighting the need for interventions to target not only individual adolescents, but also their social networks including families and peers [137,146]. In the current review, we identified three programs with peer-reviewed evaluations that were designed to generate critical reflection and dialogue about gender norms among peers, with adult role models and within communities.

In Nepal, the *CHOICES* curriculum developed by Save the Children focused on critical reflection on inequitable gender norms (so called “gender transformative” approach) among 10–14 year old boys and girls in the Siraha district. The curriculum, targeting emotions and behaviors related to gender equality, was implemented during weekly sessions held in child clubs over a three-month period. A pilot evaluation showed that *CHOICES* appeared to have changed attitudes about gender norms among young adolescents when comparing pre- and post intervention tests to those of control groups [121]. To complement the individual focus of the *CHOICES* curriculum, Save the Children subsequently developed two other interventions (not included in the current review) called *PROMISES* (targeting community gender norms) and *VOICES* (targeting parents). The latter uses testimonials of mothers and fathers to encourage other parents in the community to be more supportive of gender equality and gender norms transformation. An evaluation of the concurrent effect of these three curriculums is currently ongoing in Nepal [147].

In Honduras, Yemen, Malawi, India, Tanzania and Egypt, the *Power to Lead Alliance* (PTLA) implemented by CARE International between 2008 and 2011 worked with both young adolescent boys and girls to promote gender equality by promoting girls’ education and leadership skills through three components: 1) extracurricular activities (e.g. music, arts, sports, life skills groups, academic clubs and awareness campaigns), 2) social networks allowing girls and boys to socialize with peers and discuss issues such as early marriage and sexual relationships in “safe spaces”; and 3) civic action initiatives involving the surrounding community. A pre-post evaluation found that girls and boys from *PLTA* intervention sites had more equitable gender attitudes than girls and boys from comparison sites [49,112].

The third program included in the current review, *Parivartan*, used an adapted version of the US-based “Coaching boys into men” program to prevent gender-based violence by working with young adolescent cricket athletes in schools in Mumbai, India and specifically engaging their coaches as positive role models to challenge stereotypical gender norms and stand up against gender-based violence through bystander interventions. A 12-month non-randomized pre-post evaluation found that gender attitudes were more equitable among athletes whose coaches participated in the intervention as compared to those whose coaches received standard coaching [120].

Although these studies found some effect on a measure of gender attitudes, the interventions were substantially different from each other in their nature and the overall quality of study design so that it was not possible to draw conclusions on what factors contributed to changing gender attitudes among the participants exposed to the interventions. Overall, young adolescents have received little programmatic attention in contrast to both younger children and older peers, and of those few programs exist that have been rigorously evaluated. More research is needed to identify promising and effective interventions that challenge norms that perpetuate gender inequalities among young adolescents [137]. Taking into account the findings of this review, such research needs to develop and evaluate interventions that address the role of and interactions with parents and other family members as well as peers in addressing harmful gender stereotypes.

This review confirms other literature highlighting that early adolescence is a critical window of opportunity for countering stereotypical gender attitudes. The promotion of equitable gender attitudes is a crucial element of achieving gender equality in ways that can have many benefits for the health of adolescents as well as their health into adulthood. As the global health and development community seeks to implement the sustainable development goals (SDGs) and the 2030 development agenda, this review highlights not only the importance of focusing on early adolescence in all relevant SDGs related to health, education and gender equality, but also of the need to promote gender equitable norms in all aspects of programming and policies aimed at adolescents. Early investments in promoting gender equality in this age-group has the potential for yielding important social, economic and health benefits in the long-term as fewer harm-reduction investments will be required later in adolescence and adulthood [27].

## Conclusions

This systematic review provides a robust evidence-base of the key individual and interpersonal relationship factors that shape gender attitudes during early adolescence. Most studies were conducted in North America and Western Europe, leaving a large gap in the global peer-reviewed literature. Nevertheless, the stereotypical gender attitudes expressed by young adolescents both in LMICs and high-income countries indicate that there is still a long way to go in addressing norms that perpetuate unequal gender and power relations. Our findings highlight that programs to promote equitable gender attitudes need to target not just individuals, but their interpersonal relationships. Such programs further need to be tailored according to the unique needs of sub-populations of boys and girls. The review also shows several critical gaps in research that need to be addressed. First, the measurement of personal gender attitudes across settings is variable. Hence, there is a need to better define and standardize ways of measuring and tracking individual gender attitudes as well as social norms while maintaining their relevance across cultures. Second, we need longitudinal studies to better understand the evolving nature of gender attitudes in early adolescence and their impact on health trajectories over time, particularly as young adolescents experience onset of puberty and sexual maturation. And third, we need a better understanding of the role of communities and societal institutions such as media, schools, religious and sports among others.

## Supporting Information

S1 Table Search strategies. Detailed overview of the search terms applied to each database.
(DOCX)

S1 Text Systematic Review Protocol. Unpublished protocol outlining the background, rationale and methods for the present review.
(DOCX)

S2 Text Data extraction template. Standardized templates used to extract data on all studies according to their design (quantitative, qualitative or mixed-methods).
(XLSX)

S3 Text Qualitative codebook. Overview of the final codebook that was used to code the qualitative studies, organized by descriptive and analytical themes.
(XLSX)

S4 Text Study Summaries. Document summarizing all includes studies in the current review (author, year, study settings, study objective, theory, sampling and sample, data collection and analysis, key findings).
(DOCX)
