# Supplementary material for: Understanding Factors that Shape Gender Attitudes in Early Adolescence Globally: A Mixed-Methods Systematic Review
Source: PLoS One. 2016 Jun 24;11(6):e0157805. doi: 10.1371/journal.pone.0157805 (PMC4920358; doi:10.1371/journal.pone.0157805)
Supplement: S1 Table — Detailed overview of the search terms applied to each database. (DOCX) [file pone.0157805.s001.docx]

**S1 Table. Search Strategies.**

**Pubmed**

| 1. **Population: Young Adolescents (10-14 years)** | |
| --- | --- |
| Key words: | adolesc*[tw] OR teen*[tw] OR tween*[tw] OR youth*[tw] OR young people*[tw] OR young person*[tw] OR middle school*[tw] OR puberty[tw] OR "middle childhood"[tw] OR preadolescent[tw] |
| Controlled vocabulary: | "Child" [Mesh] OR "Adolescent" [Mesh] OR "Puberty" [Mesh] |
| **AND** | |
| 1. **Gender attitudes** | |
| Key words: | sex role*[tw] OR gender role*[tw] OR gender ident*[tw] OR gender attitud*[tw] OR gender belief*[tw] OR gender norm*[tw] OR gender stereotyp*[tw] OR gender bias*[tw] OR gendered[tw] OR gender perception*[tw] OR machismo*[tw] OR marianismo*[tw] OR macho*[tw] OR feminin*[tw] OR masculin* |
| Controlled vocabulary: | "Gender identity" [Mesh] |
| **AND** | |
| **3) Factors that influence gender attitudes/’gender socialization’** | |
| Key words: | social norm*[tw] OR social influence*[tw] OR interpersonal influence*[tw] OR expectation*[tw] OR interpersonal relation*[tw] OR socialization*[tw] OR acculturation*[tw] OR social value*[tw] OR stereotyped behavior*[tw] OR stereotyped behaviour* OR psychosexual development[tw] |
| Controlled vocabulary: | "Social perception" [Mesh] OR "Social environment" [Mesh] OR "Stereotyped Behavior" [Mesh] OR "Psychosexual development" [Mesh] OR Socialization [Mesh] |

**Date limiter: 1980-2014**

**Date of search: August 1, 2014**

**Total number of hits: 6127**

**Psychinfo**

| 1. **Population: Young Adolescents (10-14 years)** | |
| --- | --- |
| Key words: | adolesc* OR teen* OR tween OR youth* OR young people* OR young person* OR middle school* OR puberty OR middle childhood* OR pre adolescent |
| Controlled vocabulary: | AG (school age OR adolescence) OR TI (adolesc* OR teen* OR tween OR youth* OR young people* OR young person* OR middle school* OR puberty OR middle childhood* OR pre adolescent) OR AB (adolesc* OR teen* OR tween OR youth* OR young people* OR young person* OR middle school* OR puberty OR middle childhood* OR pre adolescent) |
| **AND** | |
| 1. **Gender attitudes** | |
| Key words: | sex role*[tw] OR gender role*[tw] OR gender ident*[tw] OR gender attitud*[tw] OR gender belief*[tw] OR gender norm*[tw] OR gender stereotyp*[tw] OR gender bias*[tw] OR gendered[tw] OR gender perception*[tw] OR machismo*[tw] OR marianismo*[tw] OR macho*[tw] OR feminin*[tw] OR masculin* |
| Controlled vocabulary: | (((DE "Femininity" OR DE "Feminism") OR (DE "Gender Identity")) OR (DE "Sex Roles")) OR (DE "Sex Role Attitudes") |
| **AND** | |
| 1. **Factors that influence gender attitudes/’gender socialization’** | |
| Key words: | social norm*[tw] OR social influence*[tw] OR interpersonal influence*[tw] OR expectation*[tw] OR interpersonal relation*[tw] OR socialization*[tw] OR acculturation*[tw] OR social value*[tw] OR stereotyped behavior*[tw] OR stereotyped behaviour* OR psychosexual development[tw] |
| Controlled vocabulary: | ((((((((DE "Social Norms") OR (DE "Social Perception")) OR (DE "Social Influences")) OR (DE "Social Values")) OR (DE "Interpersonal Influences")) OR (DE "Interpersonal Relationships")) OR (DE "Psychosexual Development")) OR (DE "Socialization")) OR (DE "Stereotyped Behavior") |

**Date limiter: 1980-2014**

**Date of search: August 1, 2014**

**Total number of hits: 1291**

**EMBASE/MEDLINE**

| 1. **Population: Young Adolescents (10-14 years)** | |
| --- | --- |
| Key words: | adolescent'/exp OR 'teenager'/exp OR 'tween'/exp OR 'youth'/exp OR 'puberty'/exp |
| Controlled vocabulary: | (adolesc* OR teen* OR tween* OR youth* OR 'young people' OR 'young person' OR 'middle school' OR 'middle childhood' OR preadolescent):ti,ab |
| **AND** | |
| 1. **Gender attitudes** | |
| Key words: | ('sex role' OR 'sex roles' OR 'gender role' OR 'gender roles' OR 'gender identity' OR 'gender attitude' OR 'gender attitudes' OR 'gender belief' OR 'gender beliefs' OR 'gender norm' OR 'gender norms' OR 'gender stereotype' OR 'gender stereotypes' OR 'gender bias' OR gendered OR 'gender perception' OR 'gendered perceptions' OR machismo* OR marianismo* OR macho* OR feminin* OR masculin*):ti,ab |
| Controlled vocabulary: | femininity'/exp OR 'masculinity'/exp OR 'machismo'/exp OR 'macho'/exp OR 'masculinity'/exp OR 'gender bias'/exp OR 'gender identity'/exp OR 'sex role'/exp |
| **AND** | |
| 1. **Factors that influence gender attitudes/’gender socialization’** | |
| Key words: | ('social norm' OR 'social norms' OR 'social influence' OR 'social influences' OR 'interpersonal influence' OR 'interpersonal influences' OR expectation OR expectations OR 'interpersonal relationships' OR 'interpersonal relationship' OR 'interpersonal relation' OR acculturation OR 'social value' OR 'social values' OR 'stereotyped behavior' OR 'stereotyped behaviours' OR 'stereotyped behaviour' OR 'stereotyped behaviours' OR 'psychosexual development' OR socialization):ti,ab |
| Controlled vocabulary: | psychosexual development'/exp OR 'socialization'/exp |

**Date limiter: 1980-2014**

**Date of search: August 1, 2014**

**Total number of hits: 1070**

**LILACS**

| 1. **Population: Young Adolescents (10-14 years)** | |
| --- | --- |
| Key words: | TW adolesc$ OR TW teen$ OR TW tween$ OR TW youth$ OR TW young people$ OR TW young person$ OR TW middle school$ OR TW puberty OR TW middle childhood OR TW preadolescent |
| Controlled vocabulary: | MH Child OR MH Adolescent OR MH Puberty |
| **AND** | |
| 1. **Gender attitudes** | |
| Key words: | TW sex role$ OR TW gender role$ OR TW gender ident$ OR TW gender attitud$ OR TW gender belief$ OR TW gender norm$ OR TW gender stereotyp$ OR TW gender bias$ OR TW gendered OR TW gender perception$ OR TW machismo$ OR TW marianismo$ OR TW macho$ OR TW feminin$ OR TW masculin$ |
| Controlled vocabulary: | MH Gender identity OR MH Masculinity OR MH Femininity OR MH Sexism |
| **AND** | |
| 1. **Factors that influence gender attitudes/’gender socialization’** | |
| Key words: | TW social norm$ OR TW social influence$ OR TW interpersonal influence$ OR TW expectation$ OR TW interpersonal relation$ OR TW modeling$ OR TW acculturation$ OR TW social value$ OR TW stereotyped behavior$ |
| Controlled vocabulary: | MH Interpersonal Relations OR MH Social Perception OR MH Social Environment OR MH Stereotyped Behavior OR MH Psychosexual Development OR MH Socialization OR MH Acculturation OR MH Social Values |

**Date limiter: 1980-2014**

**Date of search: August 1, 2014**

**Total number of hits: 331**

**ERIC**

| 1. **Population: Young Adolescents (10-14 years)** | | |
| --- | --- | --- |
| Key words: | | ti(adolesc* OR teen* OR tween* OR youth* OR "young people" OR "young person" OR "middle school" OR "middle childhood" OR preadolescent) OR ab(adolesc* OR teen* OR tween* OR youth* OR "young people" OR "young person" OR "middle school" OR "middle childhood" OR preadolescent) |
| Controlled vocabulary: | | SU("Preadolescent") OR SU("Adolescents") OR SU("Early Adolescents") OR SU("Middle schools") OR SU("Puberty") OR SU("youth") |
| **AND** | | |
| 1. **Gender attitudes** | | |
| Key words: | | sex role* OR 'gender role' OR 'gender identity' OR 'gender attitude' OR 'gender attitudes' OR 'gender belief' OR 'gender beliefs' OR 'gender norm' OR 'gender norms' OR 'gender stereotype' OR 'gender stereotypes' OR 'gender bias' OR gendered OR 'gender perception' OR 'gendered perceptions' OR machismo* OR marianismo* OR macho* OR feminin* OR masculin* |
| Controlled vocabulary: | | SU("Femininity") OR SU("Gender bias") OR SU("Gender role") OR SU("Gender stereotypes") OR SU("Masculinity") OR SU("Sex role") OR SU("Sex stereotypes") |
| **AND** | | |
| 1. **Factors that influence gender attitudes/’gender socialization’** | | |
| Key words: | ti(social norm' OR 'social norms' OR 'social influence' OR 'social influences' OR 'interpersonal influence' OR 'interpersonal influences' OR expectation OR expectations OR 'interpersonal relationships' OR 'interpersonal relationship' OR 'interpersonal relation' OR acculturation OR 'social value' OR 'social values' OR 'stereotyped behavior' OR 'stereotyped behaviours' OR 'stereotyped behaviour' OR 'stereotyped behaviours' OR 'psychosexual development' OR socialization) OR ab(social norm' OR 'social norms' OR 'social influence' OR 'social influences' OR 'interpersonal influence' OR 'interpersonal influences' OR expectation OR expectations OR 'interpersonal relationships' OR 'interpersonal relationship' OR 'interpersonal relation' OR acculturation OR 'social value' OR 'social values' OR 'stereotyped behavior' OR 'stereotyped behaviours' OR 'stereotyped behaviour' OR 'stereotyped behaviours' OR 'psychosexual development' OR socialization) | |
| Controlled vocabulary: | SU("Expectations") OR SU("Interpersonal relationship") OR SU("Social attitudes") OR SU("Social influences") OR SU("Social values") OR SU("Socialization") | |

**Date limiter: 1980-2014**

**Date of search: August 1, 2014**

**Total number of hits: 1436**

**GLOBAL HEALTH**

| 1. **Population: Young Adolescents (10-14 years)** | |
| --- | --- |
| Key words: | (adolesc* OR teen* OR tween* OR youth* OR young people* OR young person* OR middle school* OR puberty OR "middle childhood" OR preadolescent).ti,ab. |
| Controlled vocabulary: | adolescents/ OR youth/ OR children/ |
| **AND** | |
| 1. **Gender attitudes** | |
| Key words: | (sex role* OR gender role* OR gender ident* OR gender attitud* OR gender belief* OR gender norm* OR gender stereotyp* OR gender bias* OR gendered OR gender perception* OR machismo* OR marianismo* OR macho* OR feminin* OR masculin*).ti, ab. |
| Controlled vocabulary: | gender relations/ |
| **AND** | |
| 1. **Factors that influence gender attitudes/’gender socialization’** | |
| Key words: | (social norm* OR social influence* OR interpersonal influence* OR expectation* OR interpersonal relation* OR acculturation OR social value* OR stereotyped behavior* OR stereotyped behaviour* OR "psychosexual development" OR socialization).ti,ab. |
| Controlled vocabulary: | socialization/ OR interpersonal relation/ OR acculturation/ |

**Date limiter: 1980-2014**

**Date of search: August 1, 2014**

**Total number of hits: 82**

**SCOPUS**

| 1. **Population: Young Adolescents (10-14 years)** | |
| --- | --- |
| Key words: | (TITLE-ABS-KEY(adolesc*) OR TITLE-ABS-KEY(teen*) OR TITLE-ABS-KEY(tween*) OR TITLE-ABS-KEY(youth*) OR TITLE-ABS-KEY(young people*) OR TITLE-ABS-KEY(middle school*) OR TITLE-ABS-KEY(puberty) OR TITLE-ABS-KEY(middle childhood*) OR TITLE-ABS-KEY(preadolescent)) |
| **AND** | |
| 1. **Gender attitudes** | |
| Key words: | (TITLE-ABS-KEY(sex role*) OR TITLE-ABS-KEY(gender role*) OR TITLE-ABS-KEY(gender ident*) OR TITLE-ABS-KEY(gender attitud*) OR TITLE-ABS-KEY(gender belief*) OR TITLE-ABS-KEY(gender norm*) OR TITLE-ABS-KEY(gender stereotyp*) OR TITLE-ABS-KEY(gender bias*) OR TITLE-ABS-KEY(gendered) OR TITLE-ABS-KEY(gender perception*) OR TITLE-ABS-KEY(machismo*) OR TITLE-ABS-KEY(macho*) OR TITLE-ABS-KEY(feminin*) OR TITLE-ABS-KEY(masculin*)) |
| **AND** | |
| 1. **Factors that influence gender attitudes/’gender socialization’** | |
| Key words: | (TITLE-ABS-KEY(social norm*) OR TITLE-ABS-KEY(social influence*) OR TITLE-ABS-KEY(interpersonal influence*) OR TITLE-ABS-KEY(expectation) OR TITLE-ABS-KEY(interpersonal relation) OR TITLE-ABS-KEY(modeling) OR TITLE-ABS-KEY(acculturation) OR TITLE-ABS-KEY(social value*) OR TITLE-ABS-KEY(stereotyped behavior*) OR TITLE-ABS-KEY(stereotyped behaviour) OR TITLE-ABS-KEY(psychosexual development) OR TITLE-ABS-KEY(socialization)) |

**Date limiter: 1980-2014**

**Date of search: August 1, 2014**

**Total number of hits: 2470**

**SOCIOLOGICAL ABSTRACTS**

| 1. **Population: Young Adolescents (10-14 years)** | | |
| --- | --- | --- |
| Key words: | | (TI,AB(adolesc*) OR TI,AB(teen*) OR TI,AB(tween*) OR TI,AB(youth*) OR TI,AB(young people*) OR TI,AB(middle school*) OR TI,AB(puberty) OR TI,AB(middle childhood*) OR TI,AB(preadolescent)) |
| Controlled vocabulary: | | SU.EXACT(Adolescents) OR SU.EXACT(Youth) OR SU.EXACT(Junior High School Students) OR SU.EXACT(Puberty) OR SU.EXACT(Children) |
| **AND** | | |
| 1. **Gender attitudes** | | |
| Key words: | | TI,AB(sex role*) OR TI,AB(gender role*) OR TI,AB(gender ident*) OR TI,AB(gender attitud*) OR TI,AB(gender belief*) OR TI,AB(gender norm*) OR TI,AB(gender stereotyp*) OR TI,AB(gender bias*) OR TI,AB(gendered) OR TI,AB(gender perception*) OR TI,AB(machismo*) OR TI,AB(macho*) OR TI,AB(feminin*) OR TI,AB(masculin*) |
| Controlled vocabulary: | | SU.EXACT(Sex Role Attitudes) OR SU.EXACT(Sex Role Identity) OR SU.EXACT(Sex Role Orientations) OR SU.EXACT.EXPLODE("Sex Roles" OR "Womens Roles") OR SU.EXACT(Sex Stereotypes) OR SU.EXACT(Feminine) OR SU.EXACT(Femininity) OR SU.EXACT(Masculinity) |
| **AND** | | |
| 1. **Factors that influence gender attitudes/’gender socialization’** | | |
| Key words: | (TI,AB(social norm*) OR TI,AB(social influence*) OR TI,AB(interpersonal influence*) OR TI,AB(expectation) OR TI,AB(interpersonal relation) OR TI,AB(modeling) OR TI,AB(acculturation) OR TI,AB(social value*) OR TI,AB(stereotyped behavior*) OR TI,AB(stereotyped behaviour) OR TI,AB(psychosexual development) OR TI,AB(socialization)) | |
| Controlled vocabulary: | SU.EXACT("Norms") OR SU.EXACT.EXPLODE("Parental Influence" OR "Peer Influence" OR "Social Influence") OR SU.EXACT(Expectations) OR SU.EXACT(Acculturation) OR SU.EXACT(Social Values) OR SU.EXACT(Socialization) OR SU.EXACT(Internalization) OR SU.EXACT("Peer Relations") OR SU.EXACT("Interpersonal Relations") OR SU.EXACT("Family Relations") OR SU.EXACT("Friendship") OR SU.EXACT("Intergenerational Relations") | |

**Date limiter: 1980-2014**

**Date of search: August 2, 2014**

**Total number of hits: 2517**

**IMSEAR**

| 1. **Population: Young Adolescents (10-14 years)** | |
| --- | --- |
| Key words: | (title:adolesc* OR title:teen* OR title:tween* OR title:youth* OR title:(young people*) OR title:(young person*) OR title:(middle school*) OR title:puberty OR title:(middle childhood) OR title:preadolescent OR abstract:adolesc* OR abstract:teen* OR abstract:tween* OR abstract:youth* OR abstract:(young people*) OR abstract:(young person*) OR abstract:(middle school*) OR abstract:puberty OR abstract:(middle childhood) OR abstract:preadolescent OR keyword:adolesc* OR keyword:teen* OR keyword:tween* OR keyword:youth* OR keyword:(young people*) OR keyword:(young person*) OR keyword:(middle school*) OR keyword:puberty OR keyword:(middle childhood) OR keyword:preadolescent) |
| **AND** | |
| 1. **Gender attitudes** | |
| Key words: | (title:(sex role*) OR title:(gender role*) OR title:(gender ident*) OR title:(gender attitud*) OR title:(gender belief*) OR title:(gender norm*) OR title:(gender stereotyp*) OR title:(gender bias*) OR title:gendered OR title:(gender perception*) OR title:machismo* OR title:marianismo* OR title:macho* OR title:feminin* OR title:masculin* OR abstract:(sex role*) OR abstract:(gender role*) OR abstract:(gender ident*) OR abstract:(gender attitud*) OR abstract:(gender belief*) OR abstract:(gender norm*) OR abstract:(gender stereotyp*) OR abstract:(gender bias) OR abstract:gendered OR abstract:(gender perception*) OR abstract:machismo* OR abstract:marianismo* OR abstract:macho* OR abstract:feminin* OR abstract:masculin* OR keyword:(sex role*) OR keyword:(gender role*) OR keyword:(gender ident*) OR keyword:(gender attitud*) OR keyword:(gender belief*) OR keyword:(gender norm*) OR keyword:(gender stereotyp*) OR keyword:(gender bias) OR keyword:gendered OR keyword:(gender perception*) OR keyword:machismo* OR keyword:marianismo* OR keyword:macho* OR keyword:feminin* OR keyword:masculin*) |
| **AND** | |
| 1. **Factors that influence gender attitudes/’gender socialization’** | |
| Key words: | (title:(social norm*) OR title:(social influence*) OR title:(interpersonal influence*) OR title:(expectation*) OR title:(interpersonal relation*) OR title:(modeling) OR title:(acculturation) OR title:(social value*) OR title:(stereotyped behavior*) OR title:(stereotyped behaviour*) OR title:(psychosexual development) OR title:socialization OR abstract:(social norm*) OR abstract:(social influence*) OR abstract:(interpersonal influence*) OR abstract:(expectation*) OR abstract:(interpersonal relation*) OR abstract:(modeling) OR abstract:(acculturation) OR abstract:(social value*) OR abstract:(stereotyped behavior*) OR abstract:(stereotyped behaviour*) OR abstract:(psychosexual development) OR abstract:socialization OR keyword:(social norm*) OR keyword:(social influence*) OR keyword:(interpersonal influence*) OR keyword:(expectation*) OR keyword:(interpersonal relation*) OR keyword:(modeling) OR keyword:(acculturation) OR keyword:(social value*) OR keyword:(stereotyped behavior*) OR keyword:(stereotyped behaviour*) OR keyword:(psychosexual development) OR keyword:socialization) |

**Date limiter: 1980-2014**

**Date of search: August 2, 2014**

**Total number of hits: 64**

**AIM**

| 1. **Population: Young Adolescents (10-14 years)** | | |
| --- | --- | --- |
| Key words: | | adolescent OR adolescents OR adolescence OR teen OR teens OR tween OR tweens OR youth OR youths OR “young people” OR “young person” OR “middle school” OR “middle schools” OR “middle schooler” OR “middle schoolers” OR puberty OR "middle childhood" OR preadolescent |
| **AND** | | |
| 1. **Gender attitudes** | | |
| Key words: | | “sex role” OR “sex roles” OR “gender role” OR “gender roles” OR “gender identity” OR “gender identities” OR “gender identification” OR “gender attitude” OR “gender attitudes” OR “gender belief” OR “gender beliefs” OR “gender norm” OR “gender norms” OR “gender stereotype” OR “gender stereotypes” OR “gender bias” OR “gender biases” OR gendered OR gender perception” OR “gender perceptions” OR machismo OR marianismo OR macho OR feminine OR femininity OR masculine or masculinity |
| **AND** | | |
| 1. **Factors that influence gender attitudes/’gender socialization’** | | |
| Key words: | “social norm” OR “social norms” OR “social influence” OR “social influences” OR “interpersonal influence” OR “interpersonal influences” OR expectation OR expectations OR “interpersonal relation” OR “interpersonal relations” OR “interpersonal relationship” OR “interpersonal relationships” OR modeling OR acculturation OR “social value” OR “social values” OR “stereotyped behavior” OR “stereotyped behaviors” OR “stereotyped behaviour” OR “stereotyped behaviours” OR “psychosexual development” OR socialization | |

**Date limiter: 1980-2014**

**Date of search: August 1, 2014**

**Total number of hits: 1**

**IMEMR**

| 1. **Population: Young Adolescents (10-14 years)** | |
| --- | --- |
| Key words: | adolescent OR adolescents OR adolescence OR teen OR teens OR tween OR tweens OR youth OR youths OR “young people” OR “young person” OR “middle school” OR “middle schools” OR “middle schooler” OR “middle schoolers” OR puberty OR "middle childhood" OR preadolescent |
| **AND** | |
| 1. **Gender attitudes** | |
| Key words: | “sex role” OR “sex roles” OR “gender role” OR “gender roles” OR “gender identity” OR “gender identities” OR “gender identification” OR “gender attitude” OR “gender attitudes” OR “gender belief” OR “gender beliefs” OR “gender norm” OR “gender norms” OR “gender stereotype” OR “gender stereotypes” OR “gender bias” OR “gender biases” OR gendered OR gender perception” OR “gender perceptions” OR machismo OR marianismo OR macho OR feminine OR femininity OR masculine or masculinity |
| **AND** | |
| 1. **Factors that influence gender attitudes/’gender socialization’** | |
| Key words: | “social norm” OR “social norms” OR “social influence” OR “social influences” OR “interpersonal influence” OR “interpersonal influences” OR expectation OR expectations OR “interpersonal relation” OR “interpersonal relations” OR “interpersonal relationship” OR “interpersonal relationships” OR modeling OR acculturation OR “social value” OR “social values” OR “stereotyped behavior” OR “stereotyped behaviors” OR “stereotyped behaviour” OR “stereotyped behaviours” OR “psychosexual development” OR socialization |

**Date limiter: 1980-2014**

**Date of search: August 1, 2014**

**Total number of hits: 13**

**WPRIM**

| 1. **Population: Young Adolescents (10-14 years)** | |
| --- | --- |
| Key words: | adolescent OR adolescents OR adolescence OR teen OR teens OR tween OR tweens OR youth OR youths OR “young people” OR “young person” OR “middle school” OR “middle schools” OR “middle schooler” OR “middle schoolers” OR puberty OR "middle childhood" OR preadolescent |
| **AND** | |
| 1. **Gender attitudes** | |
| Key words: | “sex role” OR “sex roles” OR “gender role” OR “gender roles” OR “gender identity” OR “gender identities” OR “gender identification” OR “gender attitude” OR “gender attitudes” OR “gender belief” OR “gender beliefs” OR “gender norm” OR “gender norms” OR “gender stereotype” OR “gender stereotypes” OR “gender bias” OR “gender biases” OR gendered OR gender perception” OR “gender perceptions” OR machismo OR marianismo OR macho OR feminine OR femininity OR masculine or masculinity |
| **AND** | |
| 1. **Factors that influence gender attitudes/’gender socialization’** | |
| Key words: | “social norm” OR “social norms” OR “social influence” OR “social influences” OR “interpersonal influence” OR “interpersonal influences” OR expectation OR expectations OR “interpersonal relation” OR “interpersonal relations” OR “interpersonal relationship” OR “interpersonal relationships” OR modeling OR acculturation OR “social value” OR “social values” OR “stereotyped behavior” OR “stereotyped behaviors” OR “stereotyped behaviour” OR “stereotyped behaviours” OR “psychosexual development” OR socialization |

**Date limiter: 1980-2014**

**Date of search: August 1, 2014**

**Total number of hits: 11**

**Example: relevant paper’s combinations of terms**

**Comparisons of female and male early adolescent sex role attitude and behavior development**

- Adolescent
- Female
- Gender Identity*
- Humans
- Identification (Psychology)*
- Male
- Parent-Child Relations
- Personality Tests
- Psychometrics
- Psychosexual Development*
- Puberty/psychology
- Sexual Behavior*
- Social Environment

**Development of gender attitude traditionality across middle childhood and adolescence**

- Adolescent
- Attitude*
- Child
- Culture*
- Family/psychology
- Female
- Gender Identity*
- Humans
- Male
- Sexual Behavior*

Social Perception*

**Sibling influences on gender development in middle childhood and early adolescence: a longitudinal study**

- Adolescent
- Adolescent Psychology
- Adult
- Birth Order
- Child
- Child Development*
- Child Psychology
- Female
- Follow-Up Studies
- Gender Identity*
- Humans
- Longitudinal Studies
- Male
- Models, Psychological
- Nuclear Family/psychology*
- Parent-Child Relations

Socialization*

**Family context and gender role socialization in middle childhood: comparing girls to boys and sisters to brothers**

- Child
- Child Behavior/psychology
- Child Psychology
- Family/psychology*
- Female
- Follow-Up Studies
- Gender Identity*
- Humans
- Male
- Parent-Child Relations
- Parents/psychology
- Personality Assessment
- Sibling Relations*
- Socialization*

**Parental influence on children's socialization to gender roles. (review)¨**

- Adolescent
- Child
- Female
- Gender Identity*
- Humans
- Male
- Parent-Child Relations*
- Social Environment
- Socialization*
- Stereotyping
