# Supplementary material for: Understanding Factors that Shape Gender Attitudes in Early Adolescence Globally: A Mixed-Methods Systematic Review
Source: PLoS One. 2016 Jun 24;11(6):e0157805. doi: 10.1371/journal.pone.0157805 (PMC4920358; doi:10.1371/journal.pone.0157805)
Supplement: S1 Text — Unpublished protocol outlining the background, rationale and methods for the present review. (DOCX) [file pone.0157805.s002.docx]

**An ecological framework for understanding factors that shape gender attitudes in early adolescence: A systematic review**

**DRAFT PROTOCOL**

**JULY 2014**

**(Late updated: August 2015)**

Department of Population, Family and Reproductive Health

Johns Hopkins Bloomberg School of Public Health

Department of Reproductive Health and Research

World Health Organization, Geneva


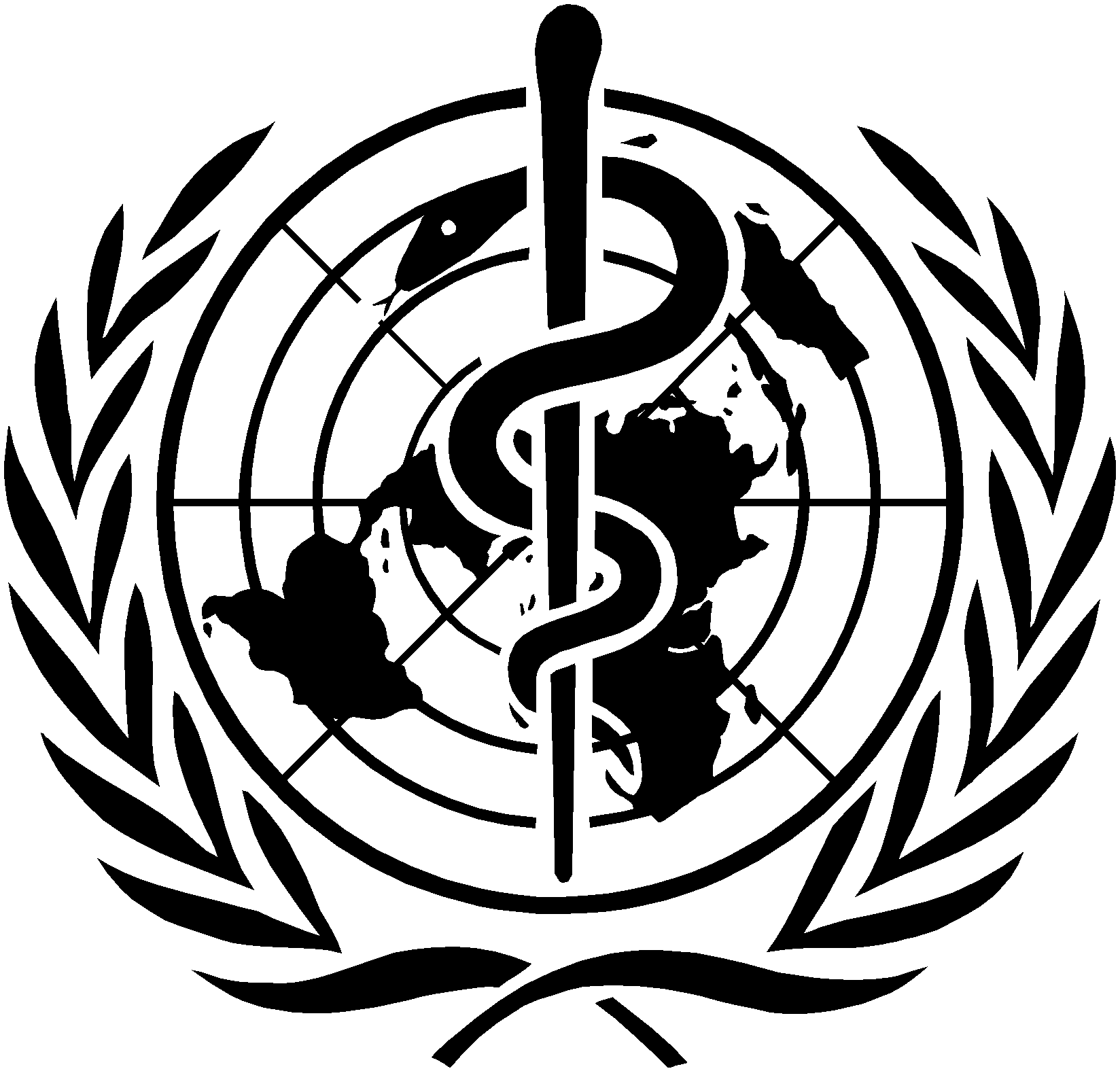


Table of Contents

1. Introduction 2

1.1. Project Background 2

1.2 Systematic review project team 2

1.3 Rationale 2

1.4 Measuring ‘gender’ 3

2. Objectives of the systematic review 4

3. Criteria for considering studies for this review 4

3.1 General inclusion/exclusion criteria 4

3.2 Type of study designs 4

3.3. Type of participants 5

3.4 Type of outcomes 5

3.5 Type of exposures 5

4. Search strategy for identification of studies 5

4.1. Electronic databases searching 5

4.2. Other electronic searching 6

5. Methods of the Review 6

4.1 Screening and data extraction form 6

4.2 Management of studies and data extraction 6

4.3 Pilot review 6

4.4 Critical appraisal of studies 7

4.5 Synthesizing of data 8

# Introduction

The purpose of this protocol is to guide the conduct of the systematic review outlined below and will serve as a blueprint for the review methodology.

## Project Background

The World Health Organization (HRP/RHR) is collaborating with the Johns Hopkins Bloomberg School of Public Health (JHSPH) to carry out the Global Early Adolescent Study (GEAS). The goal of the study is to understand the development of gender norms and attitudes in early adolescence that predispose young people to subsequent sexual health risks and conversely that contribute to healthy sexuality so as to provide the knowledge base for adult caregivers and young people themselves to improve sexual and reproductive health outcomes.

The aims of the GEAS are:

1. To develop new instruments for understanding gender norms as the basis for program development and parent support for early adolescents.
2. To understand the transitions into adolescence across cultures and the ways gender norms are expressed in adolescent relationships;
3. To understand how gender norms about relationships evolve during the first 5 years of adolescence (10-14 years of age)
4. To explore associations between gender norms and healthy sexuality in early adolescence; and with sexual behavior, relationship formation, and health outcomes during the later adolescent years.

The study will be carried out in two phases. Phase 1 is focused on instrument development and piloting to set the stage for longitudinal studies in the participating countries in Phase 2.

The specific objectives of Phase 1 of the study are:

- To conduct a systematic review of the literature on factors that influence gender attitudes globally as it pertains to young adolescents;
- To develop and test four new cross-cultural instruments to assess gender norms about relationships, healthy sexuality and related health behaviors;
- To carry out a qualitative and quantitative analysis of gender norms and healthy sexuality among early adolescents across diverse cultural settings.

While the proposed systematic review is an objective on its own, it will also contribute to the development of gender norms tools referred to above.

## 1.2 Systematic review project team

The current review will be lead by Ms. Anna Kågesten, MPH and carried out in collaboration with Ph.D. students at JHSPH. An advisory group consisting of Prof. Robert Blum and Dr Caroline Moreau of JHSPH and Dr Avni Amin and Dr Venkatraman Chandra-Mouli at HRP will oversee the conceptualization and implementation of the review. In order to ensure the quality of the review, and that it is relevant to its end users, the advisory board will also include representatives from the GEAS study sites.

## 1.3 Rationale

The ages 10-14 years are among the most critical for human development, yet one of the most poorly understood periods of the life course. During this period of *early adolescence*, young people experience intense physical, psychological and social changes that initiate the transition from childhood to adulthood. Along with pubertal changes that establish adult appearance and reproductive capacity, this age is also marked by equally dramatic changes in social, brain and cognitive maturation, which set the stage for lifelong capacities and aspirations. While these biological processes are universal, the social contexts within which they occur vary considerably. However, in most social contexts, puberty generally signals the time young people are increasingly expected to assume socially defined adult gender roles.

Societies across the globe and history have employed a wide array of strategies and mechanisms for regulating roles of males and females. Sometimes referred to as ‘gender socialization’ this can be though of as the process of learning and processing societal norms about gender that shape girls’ and boys’ understanding of themselves, their beliefs about gender, and their behaviors including their relationships (both social and sexual). From the moment they are born, children receive guidance on how to embrace or reject dimensions of the human experience as feminine and others as masculine; Such ‘rules’ for how to “be” girls and how to be “be” boys are typically expressed by multiple different agents (e.g. parents, siblings, peers, community, media), who overtly and covertly suggest, guide, model and reinforce appropriate (or discourage inappropriate) gender roles and interactions.

While children internalize messages about gender from very early ages, scholars suggest a substantial peak during transitions into adolescence. The gender intensification hypothesis, introduced by Hill & Lynch in 1983, proposes that girls and boys face increased pressure to conform to culturally defined and sanctioned gender roles during early adolescence. In brief, the hypothesis states that increased pressures from parents, peers, teachers and media leads to an intensification in gender beliefs, attitudes, roles and behaviors. These changes, in turn, are hypothesized to explain differential trajectories in health that become apparent for boys and girls during adolescence. Specifically, during adolescence diverging mortality and morbidity patterns emerge by gender, with young girls facing an increased risk of sexually coercive interactions, contracting STIs (including HIV), as well as suffering the gender-specific consequences of unintended pregnancies and psychological trauma. So too, girls face double the risk of developing depression during adolescence compared to boys while boys face increasing risk of physical injuries.

Over the recent decades, scholars have debated the Hill & Lynch’s gender intensification hypothesis and attempted to explain if and *why* this might occur. In doing so, most studies have focused on the role of parents and peers as gender socializing ‘agents’. It is however widely recognized that a number of interacting factors at individual, family, peer, school, community and societal levels influence gender beliefs, roles and behaviors during both childhood and adolescence (see for example Galambos, 2004). Yet, to our knowledge no study has attempted to summarize what is known about the key factors that contribute to gender norms during the earliest years of adolescence (ages 10-14). Furthermore, most studies have been conducted in the US or other western settings; little is known about mechanisms that influence gender norms and gendered behavior from a global perspective, and whether there are a set of contributing factors that cut across different settings. Increased understanding of the key domains that influence gender norms and gendered behavior can help guide gender transformative interventions aimed at improving the health trajectories of young boys and girls including their sexual and reproductive health outcomes.

## 1.4 Measuring ‘gender’

Despite being one of most well known indicators in public health studies, gender is a multi-faceted, diverse and complex measure. Different schools of thought have structured the way gender has been conceptualized as it relates to behavioral and health outcomes. An *essentialist* approach considers gender as a personality trait hard-wired within individuals, much like being born a male or female. Today, few people subscribe to this notion. From a *social cognitive learning perspective*, gender is considered a social process; and research concentrates on the extent to which individuals embrace socially determined gender norms and how these beliefs inform gendered behaviors. An important contribution of this approach is the recognition that gender is a multidimensional construct, with masculinity and femininity representing distinct and complementary dimensions that independently inform behaviors of both sexes. This approach however, fails to recognize the dialectical process of gender as reinterpreted and reconstructed by individuals who in turn influence dominant norms of femininity and masculinity. Such a dialectical conceptualization of gender is delineated in a *social constructionist* approach that positions young people not only as enacting but also as actively engaged with shaping and even changing gender norms, which they endorse, resist or alter as they enact gender in their interpersonal relationships.

Importantly, any attempt to synthesize information from studies of gender-related issues need to reconcile the different perspectives on gender that are reflected in the scientific disciplines (sociology, psychology, medicine, public health) in studies on the manifestations of gender in social interactions or the relationship between gender and health. Studies use a range of different outcomes measures such as gender norms, gender beliefs, gender ideologies, gender identity, gender roles, etc. For the purpose of this protocol, and for the sake of simplicity, we will use the term “gender attitudes” while referring to beliefs, perceptions or values about gender held by individuals. We feel that restricting the review to one outcome (such as norms *or* attitudes) would not allow us to identify the broad array of measures used in studies that are relevant to our study objectives.

# Objectives of the systematic review

This review seeks to explore what is known about the correlated factors that shape or potentially influence gender attitudes in early adolescence – that is, about the key family, peer, school, community and media factors that shape or potentially influence young adolescent’s attitudes about gender.

**The initial steps of review will explore the following questions:**

- What ecological-level factors are associated with gender attitudes in early adolescence (individual, family, peer, media, school)?
- How do young adolescents learn about and construct gender attitudes in relation to their social environments?
- To what extent are these mechanisms or factors similar across different cultural settings (in different parts of the world)?

# Criteria for considering studies for this review

This protocol was developed in line with the *PRISMA* guidelines for reporting on systematic reviews (Note: the final systematic review manuscript is formatted according to ENTREQ guidelines given the mixed-method synthesis)

## 3.1 General inclusion/exclusion criteria

The review process will consider the following general inclusion criteria during the first screening:

- Studies published in all parts of the world.
- Abstracts in English, French, Spanish.

Studies will be excluded if there is no data or if no date or source of data collection is provided. We will also exclude studies that focus solely on gender theories and theoretical frameworks, i.e. do not have any participants.

## 3.2 Type of study designs

We choose not to limit the search by study design in order to capture as many relevant studies as possible. As noted by Armstrong et al (2007), non-randomized studies may better represent the best available evidence (than randomized controlled trials) for some questions. In the case of young adolescents, many of the social and contextual factors that influence adolescent development are not possible to randomize. In addition, qualitative studies are important as they can help answer questions that are not possible to explore with quantitative survey research (e.g. how gender beliefs are shaped in different settings).

## 3.3. Type of participants

Studies should include young adolescent participants (aged 10-14). If studies include adolescents of different ages, disaggregation by age group should be possible. Studies that focus solely on younger children (<10) or older adolescents (>14) are beyond the scope of this review. If studies meet all criteria but focus on older adolescents (15 and above), these studies will be saved in a separate library for potential inclusion as references and for purposes of comparison in the final paper.

## 3.4 Type of outcomes

The key outcome or phenomenon of interest, *gender attitudes*, is defined as ideas and attitudes that an individual holds about the roles, behaviors, values and power of men and women, which guide his or her behavior. Social norms about gender are central in the construction of individual beliefs.

As mentioned above, measures of gender attitudes can however conceptualized in many different ways depending both on the type of study (i.e. qualitative or quantitative), as well as the theoretical paradigm and scientific discipline guiding the study. During the search and screening, we therefore apply a broad conceptualization of gender attitudes. Examples of terminology that *can* be applicable to the review include: gender norms, gender ideologies, gender identity, gender roles, gender perceptions, masculinity norms, femininity norms, femininities, masculinities.

These outcomes can be measured by a wide array of tools, such as scales (i.e. the GEM - gender equitable norms scale) and survey questions. Qualitative phenomenon of interest will likely be in narrative forms.

For the purpose of this review, we will not include studies that focus on biological sex differences; i.e. that merely compare outcomes between males and females. Rather, we want to explore how young adolescents, irrespective of biological sex, learn to act and think in certain ways when it comes to gender.

## 3.5 Type of exposures

Studies should explore one or more factors that contribute to gender attitudes in early adolescence. While the acquisition of gender attitudes can occur through a variety of channels, we are primarily interested in the role of factors at different levels of the ecological framework, including individual family, peers, media and school factors.

# Search strategy for identification of studies

Reviewers will be able to read English, French, German, Spanish, Swedish, Norwegian and Danish. Reports with abstracts in any of these languages that have full-texts in other languages (e.g. Mandarin, Russian) will be translated into English.

A core search strategy of key words will be developed for one database (PubMed). This strategy will be adapted for others to include specific Mesh terms.

## Electronic databases searching

The following electronic databases will be searched: PsycInfo, PubMEd, Global Health, ERIC, Scopus, Sociological Abstracts, MEDLINE, EMBASE, Latin American and Caribbean Health Sciences Information (LILACS).

A number of WHO on-line databases will also be searched: Index Medicus for the WHO, South East Asian region (IMSEAR), African Index Medicus (AIM), the West Pacific Index Medicus (WPIM).

## Other electronic searching

While we initially planned to search the grey literature, a decision was made following the database screening to restrict the review to peer-reviewed literature. This was a logistical decision spurred by the large number of database records obtained.

# Methods of the Review

## 4.1 Screening and data extraction form

A set of screening questions will be available for the title and abstract evaluation (See Annex 1). This form includes 8 questions, collecting information on inclusion decision and potential reasons for exclusion. Using WHO systematic review protocol templates, a data extraction form will be developed and modified according to the specific review aims. This form includes questions designed to collect information on study design, sampling, methods, data analysis and key results. Both the screening questions and data extraction form will be pilot tested.

## 4.2 Management of studies and data extraction

The review will use Endnote bibliographic software to store and keep track of citations. A shared library will be created between the reviewers. Electronic searches will be downloaded directly into Endnote while studies retrieved from other sources will be entered in Endnote manually. Duplicates will be deleted and each study will be assigned a unique identification number for the review.

All citations identified by the electronic search strategies will be evaluated initially according to the screening form on the basis of the titles and abstracts. In the absence of abstracts, table of contents and summaries in reports will be screened. Irrelevant records will be discarded. Full text of relevant studies will then be obtained and screened in duplicate (by two reviewers), as will studies whose available citations did not provide sufficient information to decide. Studies excluded at this stage will be listed separately with the reason for exclusion stated. A list of the irrelevant records and its search databases will be available upon request.

Data for relevant full-text studies will be extracted by three independent reviewers using the data-extraction form. When inconsistencies are identified or disagreements occur they will be resolved through discussion or consulting a fourth reviewer. Attempts will be made to contact authors to obtain missing information or clarification whenever necessary.

## 4.3 Pilot review

Before initiating the formal review, all search strategies, screening questions, data-extraction forms and review methods will be piloted during July 2014. The search strategy will encompass the entire time period and two reviewers will scan the identified studies according to the screening form. The two independent reviewers will screen a random sample of 50 references in duplicate, in order to test inter-observer variability. When inconsistencies are identified or disagreements occur they will be resolved through discussion or consulting a third reviewer.

The same two reviewers will then extract data from all identified studies. When inconsistencies are identified or disagreements occur they will be resolved through discussion or consulting a third reviewer. This will enable identification of flaws or gaps in the data-extraction form. A final version of the form, including any modifications will be prepared after piloting.

**Pilot Search strategy:**

The following search strategy, adopted according to database standards, will be used in the pilot review:

Pilot search strategy for Pubmed

| 1. **Population: Young Adolescents (10-14 years)** | |
| --- | --- |
| Key words: | adolesc*[tw] OR teen*[tw] OR tween*[tw] OR youth*[tw] OR young people*[tw] OR young person*[tw] OR middle school*[tw] OR puberty[tw] OR "middle childhood"[tw] OR preadolescent[tw] |
| Controlled vocabulary: | "Child" [Mesh] OR "Adolescent" [Mesh] OR "Puberty" [Mesh] |
| **AND** | |
| 1. **Gender norms (attitudes, beliefs, ideologies, identity)** | |
| Key words: | sex role*[tw] OR gender role*[tw] OR gender ident*[tw] OR gender attitud*[tw] OR gender belief*[tw] OR gender norm*[tw] OR gender stereotyp*[tw] OR gender bias*[tw] OR gendered[tw] OR gender perception*[tw] OR machismo*[tw] OR marianismo*[tw] OR macho*[tw] OR feminin*[tw] OR masculin* |
| Controlled vocabulary: | "Gender identity" [Mesh] |
| **AND** | |
| 1. **Factors that influence gender norms/behaviors/’socialization’** | |
| Key words: | Socialization [Mesh] OR Psychosexual development [Mesh] OR social norm* [tw] OR social environment(tw] OR social influence[tw] OR interpersonal influence* [tw] OR Stereotyped Behavior[Mesh] OR expectations [tw] OR interpersonal relationship*[tw] OR socialization OR parent influence* OR peer influence [tw] OR social influence* [tw] OR stereotyping[tw] |
| Controlled vocabulary: | "Social perception" [Mesh] OR "Social environment" [Mesh] OR "Stereotyped Behavior" [Mesh] OR "Psychosexual development" [Mesh] OR Socialization [Mesh] |

## 4.4 Critical appraisal of studies

Following the initial screening, studies that pass the full-text review will be appraised in order to discern differences in the study characteristics with regard to the study design, population, setting, methods, etc. We will use an adapted quality assessment version of the *Effective Public Health Practice Project (EPHPP) Quality Assessment Tool of Quantitative Studies* (ANNEX 2). This quality assessment will depend heavily on the type of study design used. Risk of bias will be higher for non-randomized studies; however such studies may better corresponding to real-life conditions.

For qualitative studies, we will use the *Critical Appraisal Skill Programme* (CASP) guide, including: aims, methodology, link to theory, study design, fieldwork procedures, data analysis, credibility of findings, reflexivity and ethical considerations.

## 4.5 Synthesizing of data

Because of the wide range of studies and outcomes that can quality for this review, a meta-analysis combining effect estimates such as Odds Ratios (OR) will not be possible. We will conduct a configurative mixed-methods synthesis where quantitative and qualitative data is first analysed separately and brought together in the end for a final synthesis. We will conduct a Thematic Summary of the quantitative. Factors associated with gender attitudes will be clustered into categories and common associations across studies will be summarized these as themes organized by the ecological framework. Qualitative studies will be analyzed using Thematic Synthesis. First, each relevant text-unit of the primary studies will be coded using Atlas.ti. An initial codebook will then be created and applied throughout the coding process. Using an iterative process, codes will be reviewed and refined to identify descriptive and analytical themes.

Studies will be described in line with the characteristics outlined in the data extraction form: study design, population, setting, methods, and key results. Additionally, definitions and conceptualization of gender measures will be sought and described from each study.

**ANNEX 1: Title/abstract screening Questions**

| **TITLE/ABSTRACT SCREENING QUESTIONS FOR DATABASES** | **NO** | **YES** |
| --- | --- | --- |
| 1. Does the study introduce primary data? 2. Are the study participants aged 10-14 or is data disaggregated for this age group? 3. Does the study include a gender attitude *outcome*? 4. Does the study include at least one potential antecedent of gender attitude?     ***NOTE: If any answer falls into a “No” for Question 1-3, then the study will be excluded.*** |  |  |
|  |  |  |

| **ASSESSMENT** | **NO** | **YES** |
| --- | --- | --- |
| 1. Excluded following initial screening (Titles/Abstracts) 2. Excluded following full text screening 3. If the answer to Question “5” or “6” is “Yes”, please indicate the reason for exclusion: 4. If the study is relevant (on topic), but has the wrong age group (e.g. older adolescents), please make a note and specify: ___________________________________________________________________________________________________________________________________________________________________________________________________ 5. If the study assess gender norms and behavior as an exposure for a health outcome, please make a note and specify: |  |  |

**[Data extraction templates are available in excel format]**
