# Supplementary material for: Understanding Factors that Shape Gender Attitudes in Early Adolescence Globally: A Mixed-Methods Systematic Review
Source: PLoS One. 2016 Jun 24;11(6):e0157805. doi: 10.1371/journal.pone.0157805 (PMC4920358; doi:10.1371/journal.pone.0157805)
Supplement: S4 Text — Document summarizing all includes studies in the current review (author, year, study settings, study objective, theory, sampling and sample, data collection and analysis, key findings). (DOCX) [file pone.0157805.s005.docx]

**S4 Text. Summary of included studies, sorted by study design.**

**Quantitative studies**

| **Study Nr** | **Author & Year** | **Study Setting** | **Study Objective** | **Study Design** | **Theory** | **Sampling & Sample** | **Data Collection & Analysis** | **Key Findings** | **Quality** |
| --- | --- | --- | --- | --- | --- | --- | --- | --- | --- |
| 1 | Baruch & Barnett (1986) (1) | United States  Boston (suburban) | Assess association of father’s participation in childcare and chores with children’s sex-role attitudes | Quantitative  Cross-sectional | Parsonian theory | N=80*; age 10; males and females; middle class; mostly white | School-based Interviewer- and self-administered surveys  Multiple linear regression | Overall, participants’ sex role attitudes were not associated with fathers’ participation in in the home. Children whose mothers held less traditional attitudes had less stereotypical attitudes themselves. Fathers’ participation in the home had a stronger effect on reducing children’s stereotypical attitudes among those whose mothers were employed. Males expressed more stereotypical attitudes than females. | Moderate |
| 2 | Blakemore (2003) (2) | United States  Midsized Midwestern city (urban) | Examine children’s beliefs about gender norm violations and assess variations by age and sex. | Quantitative  Cross-sectional | Gender schema theory | N=32*; ages 10-11; males and females; SES not described; mostly white | School-based survey  ANOVA and logistic regression | Older participants expressed more stereotypical beliefs about gender norms. Females were more accepting of gender norm violations compared to males. Males’ gender norm violations were less accepted than females’ gender norm violations. | Low |
| 3 | Bohannon & Blanton (1999) (3) | United States  Eastern Tennessee (*urbanicity not described*) | Assess similarity and changes over time of gender role attitudes of mothers and daughters. | Quantitative  Longitudinal cohort with 15 year follow-up | Symbolic interaction theory | N=40; ages 10-14 at baseline; females only; mixed SES; white | Postal survey  MANOVA | Participants expressed similar gender role attitudes as their mothers over time. | Low |
| 4 | Bos, Picavet & Sandfort (2012) (4) | Netherlands  Amsterdam (urban) | Examine variation in attitudes about gay men/lesbian women and gender non-conforming behaviour by ethnic background. | Quantitative  Cross-sectional | Social learning theory | N=292; ages 10-13; males and females; low SES; half had an immigrant parent from a non-Western country | School-based self-administered survey  MANOVA and ANOVA | Participants with immigrant parents expressed more negative attitudes toward gay men and lesbians and toward gender non-conforming behaviors. Those with non-Western backgrounds also indicated greater pressure from parents for gender conformity. Males held more negative attitudes toward gay men and lesbians and toward gender non-conforming behaviors compared to females. Males also reported greater peer pressure to conform to gender expectations and greater pressure from parents. | Moderate |
| 5 | Boxley, Lawrance, & Gruchow (1995) (5) | United States  Central North Carolina (rural) | Assess the association between stereotyped attitudes toward women and belief in rape myths. | Quantitative  Cross-sectional | Feminist theory | N=211; ages 11-18 (all 8^th^ grade students); males and females; SES not described; mostly white. | School-based survey  Chi-squared | Males were more likely than females to agree with traditional gender attitudes and to accept rape myths. There were no differences in overall gender attitudes and acceptance of rape myths by race or age. Participants with traditional gender attitudes were more likely to accept rape myths. | Low |
| 6 | Brown & L’Engle (2009) (6) | United States  Southeast (rural, suburban, and urban) | Examine the association between use of sexually explicit media and progressive gender role attitudes. | Quantitative  Longitudinal cohort with 2 year follow-up | Sexual socialization theory and the media practice model | N=967; mean age = 13.6 at baseline; males and females; one-third low SES; white and African American. | School- and household-based self-administered and ACASI surveys  Chi squared, linear and logistic regression | Females who viewed any sexually explicit media at baseline expressed less progressive gender role attitudes at follow-up. Males’ consumption of sexually explicit media was not associated with gender role attitudes. | High |
| 7 | Cowan & Campbell (1995) (7) | United States  Riverside County, California (*urbanicity not described*) | Explore attitudes about rape and identify factors associated with those attitudes. | Quantitative  Cross-sectional | Feminist theory and social learning theory | N=453; mean age = 14.6; males and females; SES not described; mostly white and Hispanic. | Survey at rape education programs  MANOVA, ANCOVA, paired t-tests | Male participants were more likely to endorse female precipitation as a cause of rape while female participants were more likely to endorse male dominance and pathology. Among females but not males, endorsement of precipitation as a cause of rape was lower among older participants. So too, among females overall communication about rape was associated with less endorsement of female precipitation and male sexuality, and greater endorsement of male dominance. Exposure to pornography was associated with greater endorsement of female precipitation and male sexuality as causes of rape for females only. Hispanic participants were more likely than White to endorse male sexuality as a cause of rape. | Moderate |
| 8 | Crouter et al. (2007) (8) ^a^ | United States  Northeast  (semi-urban and rural) | Assess changes in gender attitudes during childhood and adolescence and identify associations with individual and family characteristics. | Quantitative  Longitudinal cohort with 9 year follow-up and two children per family | Ecological systems theory, social learning theory of gender development | N=402; age at analytic baseline = 10; males and females; working and middle class; mostly white. | Household-based interviewer-administered survey  Hierarchical linear modelling | Males had more traditional gender attitudes, as did participants who had parents with traditional attitudes. Second born children had more traditional gender attitudes than firstborn siblings. Overall, participants’ attitudes became less traditional between ages 7 to 13 and remained stable from ages 13 to 15. Participants with certain individual and family characteristics diverged from this trajectory. For example, males with traditional parents maintained traditional attitudes throughout early adolescence and firstborn males with traditional parents and a male sibling developed increasingly traditional attitudes over the course of early adolescence. | High |
| 9 | Das et al. (2014) (9) ^b^ | India  Mumbai (urban) | Explore the association between gender attitudes, attitudes about violence against women, and violence perpetration. | Quantitative  Cross-sectional | *Not described* | N=1,040; ages 10-16, mean age = 13; males only; mixed SES. | Sports team-based survey  Chi-squared and logistic regression | Participants with more equitable gender attitudes were less likely to find violence against women acceptable. Witnessing parents’ intimate partner violence and experiencing violence at home or in the community were all associated with greater acceptance of violence against women. | Moderate |
| 10 | De Lemus, Moya & Glick (2010) (10) | Spain  Grenada  (urban) | Examine the association between romantic relationship experiences and endorsement of sexism. | Quantitative  Cross-sectional | Ambivalent sexism theory | N=1,447; ages 12-19 mean age = 14; males and females; SES not described. | School-based self-administered survey  MANOVA | Romantic/sexual relationship experience was associated with hostile sexism among early adolescent males and females. Relationship experience was associated with benevolent sexism only among early adolescent males. | Moderate |
| 11 | Emihovich, Gaier & Cronin (1984) (11) | United States  (*urbanicity not described*) | Assess association between fathers’ desired sex role attitudes for their sons and sons’ actual sex role attitudes. | Quantitative  Cross-sectional | Feminist theory and gender schema theory | N=119; mean age = 11.7; males only; middle class; white. | Household-based survey  Multiple regression analysis | Fathers’ expectations for their sons’ sex role beliefs were associated with sons’ own sex role beliefs. Fathers’ own sex role beliefs were also associated with sons’ sex role beliefs. | Low |
| 12 | Evertsson (2006) (12) | Sweden  (urban and rural) | Assess the association between parents’ division of work and gender attitudes with children’s attitudes about gender equality in household work. | Quantitative  Cross-sectional | Doing gender | N=1,304; ages 10-18 mean age = 13.5; males and females; mixed SES. | Household-based ACASI survey  Chi squared and logistic regression | Females were more likely than males to agree than gender equality in the household is important. While mothers’ time doing housework was not related to participants’ gender attitudes, high maternal education was associated with finding gender equality in the household important. For females, equitable attitudes were associated with mothers working fulltime outside the house, but not for males. Younger age and having young siblings were associated with less equal gender attitudes for males but not for females. | High |
| 13 | Foshee & Bauman (1992) (13) | United States  10 cities in the Southeast  (urban) | Understand the relationship between gender stereotyped attitudes and sexual behaviour. | Quantitative  Longitudinal cohort with 2 year follow-up | Cognitive behavioural theory | N=2,102; ages 12-14; males and females SES not described; African American and white. | Household-based self-administered survey  Logistic and linear regression | Males and females had similar gender stereotyping scores. Sexual behaviour was associated with later increases in stereotypical gender attitudes for black females while attitudes became less stereotypical for white females after initiating sexual activity. Sexual behaviour was not associated with gender attitudes for males. Lower maternal education and maternal employment were both associated with more stereotypical attitudes for males but not for females. | High |
| 14 | Funk & Buchman (1996) (14) | United States  Midwest (suburban) | Describe gender differences in gender stereotypes about playing video games. | Quantitative  Cross-sectional | *Not described* | N=364; 4^th^ and 5^th^ grade students; males and females; 10% on welfare; mostly white. | School-based survey  Chi squared | Among 5^th^ grade students, females were more likely than males to agree that either boys or girls can play video games and less likely to find it acceptable for boys to play video games a lot. Fifth grade males were also more likely than females to agree that popular boys usually play video games. Male students were also more likely to think that girls who play video games are less popular and that fighting games are mainly for boys. | Low |
| 15 | Galambos, Almeida & Petersen (1990) (15) | United States  Midwest (suburban) | Examine gender role intensification in early adolescence and effect of pubertal development. | Quantitative  Longitudinal cohort with 3 year follow-up | Gender intensification theory | N=200; ages 11-13 mean age at baseline = 11; males and females; middle and upper-middle class; mostly white. | School-based survey  ANOVA with linear and quadratic polynomial contrasts | Females were more likely to approve of egalitarian sex role attitudes. For males but not females, greater identification with femininity traits was associated with egalitarian attitudes. Egalitarian attitudes increased over time for females but not for males. Pubertal timing was not associated with egalitarian sex role attitudes. | High |
| 16 | Grose, Grabe & Kohfeldt (2014) (16) | United States  Central coastal region of California (rural) | Evaluate effect of school-based sex education intervention on attitudes and beliefs about gender and sexuality. | Quantitative  Quasi-experimental | Empowerment theory | N=95; ages 12-15 mean age = 14; males and females; mixed SES; mostly Hispanic. | School-based self-administered survey  MANOVA and SEM | Females reported marginally less traditional attitudes toward women, and marginally less agreement with masculine ideology. Participants expressed less traditional attitudes toward women and marginally less agreement with masculine ideology after exposure to the sex education curriculum. | Low |
| 17 | Hertzog & Rowley (2014) (17) | United States  Midwest (*urbanicity not described*) | Describe relationship norms and examine differences by gender. | Quantitative  Cross-sectional | Social norms theory | N=187; ages 11-19 mean age = 14.8; males and females; SES not described; mostly white and African American. | Youth conference-based self-administered survey  Paired t-tests | Attitudes about violence against women in dating relationships were similar for males and females. Males were more likely than females to find girls’ sexual harassment of boys acceptable. | Moderate |
| 18 | Hess, Ittel & Sisler (2014) (18) | Germany  Berlin  (urban) | Examine family influences in socialization of gender role orientations. | Quantitative  Longitudinal cohort with 5 year follow-up | Developmental systems theory, gender intensification theory, and social cognitive theory of gender development | N=244; mean age = 14; males and females; highly educated parents. | School-based interviewer-administered and postal surveys  Correlations matrix and SEM | For males, traditional gender role orientation was associated with fathers’ traditional gender role orientation and fathers’ gender-specific parenting. For females, traditional gender role orientation was associated with mothers’ traditional gender role orientation and fathers’ gender-specific parenting. Siblings’ gender role orientations were associated with each other. Parents’ workplace autonomy was not associated with participants’ gender role orientations. Participants from higher SES families expressed more egalitarian gender role orientations. | Moderate |
| 19 | Hoover & Fishbein (1999) (19) | United States  (Suburban) | Compare levels of prejudice by gender | Quantitative  Cross-sectional | Theory of the development of individual and social identity | N=433; ages 12-15* mean age = 13.7; males and females; upper-middles class; white. | School-based self-administered survey  MANOVA | Males expressed higher levels of sex role stereotyping than females. | Moderate |
| 20 | Jones, Fries & Danish (2007) (20) | United States  Virginia (rural) | Assess variation by gender and ethnicity in opposite and same sex body size preferences in rural adolescents. | Quantitative  Cross-sectional | *Not described* | N=384; mean age = 13.4; males and females; SES not described; African American and white. | School-based interviewer-administered survey  ANOVA and t-tests | Overall, males selected a larger ideal female figure than females' ideal female figure, although specifically among African American participants, females chose a larger female ideal figure than males. Among all participants, males and females selected a similarly sized ideal male figure. Among both males and females, African Americans chose larger ideal male and female figures than white males and females. | High |
| 21 | Katz & Ksansnak (1994) (21) | United States  Colorado (*urbanicity not described*) | Evaluate gender role flexibility across childhood and adolescence and assess influences of cognitive, socialization, and biological factors. | Quantitative  Cross-sectional | Reinforcement and modelling theories, gender intensification theory, and cognitive-developmental theory | N=176; ages 11-14*; mean age = 13.6; males and females; well-educated parents; mostly white. | School-based  Regression | Girls expressed higher tolerance for gender role flexibility than boys. Tolerance for gender role flexibility was associated with social environment, which was a composite measure of cross-gender flexibility in friends and parents. | Moderate |
| 22 | Kurtz-Costes et al. (2008) (22) | United States  Southeast (rural) | Examine associations between perceptions of adult gender stereotypes, children’s own stereotypes, and perceived math and science ability. | Quantitative  Cross-sectional | Social status theory and cultural-experiential theory | N=209*; mean age = 12.7; males and females; SES not described; mostly white and African American. | School-based survey  ANOVA and regression | Among males, beliefs that adults held traditional gender stereotypes about math and science abilities were associated with their own favourable beliefs about boys’ math and science abilities. Beliefs about adults’ stereotypes were not associated with males’ own beliefs about girls’ math and science abilities. Among females, beliefs about adult stereotypes were not associated with their own beliefs about boys' or girls' math and science abilities. | Low |
| 23 | Levant et al. (2008) (23) | United States  Midwest  (suburban)  Scotland  Glasgow (urban) | Assessment of psychometric properties of the Male Role Norms Inventory for adolescents. | Quantitative  Cross-sectional | Gender role strain paradigm | N=436; ages 11-18 mean age = 13.6; males and females; middle and upper class; mostly white. | School-based self-administered survey  ANCOVA | Males espoused more traditional norms about the male role than females. Participants in the United States expressed more traditional norms about the male role than participants in Scotland. | High |
| 24a | Liben & Bigler (2002) (24)  Monograph including 3 studies | United States  Central Pennsylvania (*urbanicity not described*) | Develop scales to measure sex-typed attitudes toward others and self relating to occupations, activities and traits. | Quantitative  Cross-sectional | Attitudinal and personal pathway models grounded in constructivist theories | N=154; 6^th^ grade students; males and females; middle class; mostly white. | School-based interviewer-administered survey  Correlations | Participants gave more egalitarian responses for masculine items than feminine items, with a greater effect size for item gender among female participants than male participants. Participants gave more egalitarian responses for attitudes about traits than for occupations and activities, also with a greater effect size among female participants than among male participants. | Moderate |
| 24b |  | United States  Sample 1: Midwest (urban)  Sample 2: Southwest (urban) | Cross-validate scales using a more diverse sample. | Quantitative  Cross-sectional | Attitudinal and personal pathway models grounded in constructivist theories | Sample 1: N=165; 6^th^ grade students; males and females; SES not described; mostly African American, white, and Native American.  Sample 2: N=33; similar to sample 1 except all white. | School-based interviewer-administered survey  Correlations | Native American and Hispanic participants expressed more egalitarian attitudes than white and African American participants. Among all participants, females were more likely to have different attitudes toward masculine versus feminine items than male participants. Males expressed more egalitarian attitudes about feminine activities than to masculine activities while females were more egalitarian about masculine activities than feminine activities. Personal traits were not associated with attitudes toward others. | Moderate |
| 24c |  | Central Pennsylvania (*urbanicity not described*) | Assess longitudinal associations between children’s sex typing of the self with sex typing of others. | Longitudinal cohort with 2 year follow-up | Attitudinal and personal pathway models grounded in constructivist theories | N=78; 6^th^ grade students; males and females; middle class; mostly white. | School-based interviewer-administered survey  ANOVA and regression | Male participants gave more egalitarian responses for feminine items than female participants and all attitudes became more egalitarian over time. Overall, there was no association of personal traits at baseline with attitudes toward others at follow-up. However, males with feminine traits at baseline had more egalitarian attitudes at follow-up. Additionally, females with interest in a masculine occupation at baseline were more likely to express an egalitarian attitude about that specific occupation at follow-up. | Low |
| 25 | Lurye, Zosuls & Ruble (2008) (25) | United States  *(urbanicity not described*) | Examine the association between rigid sex-typed beliefs and adjustment, taking into account the role of gender in children’s self-concept. | Longitudinal cohort with ~5 year follow-up | Gender schema theory and regulatory focus theory | N=59; ages 7-13 mean age = 10.3 at follow-up; males and females; SES not described; mostly white. | Survey  Analysis not described | Participants who felt pressure from parents to conform to gender stereotypes were more likely to have higher levels of sex role rigidity. Centrality and evaluation of one’s own gender role was not associated with sex role rigidity. Associations were similar for male and female participants. | Low |
| 26 | McHale, Crouter & Tucker (1999) (26) ^a^ | United States  Northeast  (semi-urban and rural) | Describe sex typing in middle childhood and assess effect of sibling gender and parent gender role attitudes. | Quantitative  Cross-sectional | *Not described* | N=200; mean age = 10.4; males and females; working or middle class; mostly white. | Household-based interviewer-administered and phone surveys  ANOVA | Females had less traditional gender attitudes than males. Less traditional parental gender attitudes were associated with less traditional participant gender attitudes. Male participants with a younger sister and a traditional father had more traditional attitudes. | Moderate |
| 27 | McHale et al. (2001) (27) ^a^ | United States  Northeast  (semi-urban and rural) | Evaluate the association between gender role attitudes of first- and second-born siblings in middle childhood and early adolescence. | Longitudinal cohort with 2 year follow-up | Social learning, sibling de-identification | N=396; firstborn mean age = 10.9 and second-born mean age = 8.2 at baseline; males and females; working or middle class; mostly white. | Household-based interviewer-administered and phone surveys  ANOVA and hierarchical regression | Firstborn participants’ attitudes became less traditional from age 10 to 11 then levelled out to age 12. For firstborn female participants, those with younger brothers had marginally less traditional attitudes than those with younger sisters and sibling attitudes were a significant negative predictor of attitudes. For firstborn male participants, those with less traditional mothers and more traditional siblings became less traditional over time. Second-born female participants’ attitudes at age 10 were associated with their older siblings' gender attitudes at baseline. For second-born male participants, gender attitudes at age 10 were associated with mothers’ and older siblings’ attitudes at baseline. | Moderate |
| 28 | McHale et al. (2004) (28) ^a^ | United States  Northeast  (semi-urban and rural) | Assess how sex-typing and social context of leisure activities influences development of gender role attitudes. | Longitudinal cohort with 2 year follow-up | Cognitive models of gender development and the ecological model | N=200; mean age = 10.8 at baseline; males and females; working or middle class; mostly white. | Household-based interviewer-administered and phone surveys  Correlations | For females, neither overall time spent in sex-typed activities nor the social context of activities (i.e. with male/female parent, sibling, or peer) were associated with gender role attitudes. Also for females, time spent playing sports and time spent with brothers was marginally associated with less traditional gender role attitudes while time spent with sisters was marginally associated with more traditional gender attitudes. For males, there was no overall association of sex-typed activities or time spent with siblings and parents with gender role attitudes. Males who spent more time alone developed less traditional attitudes over time and time spent reading was marginally associated with less traditional attitudes. | Moderate |
| 29 | Meaney, Dornier & Owens (2002) (29) | United States  (urban) | Assess sex role stereotyping of sports and physical activities. | Quantitative  Cross-sectional | *Not described* | N=250*; mean age = 13.1; males and females; diverse SES; diverse race. | School-based self-administered survey  Chi squared | Among 5^th^ grade students there were no differences between male and female participants’ gender stereotyping of sports. Among 8^th^ grade students, females were more likely than males to rate football, pool and weight lifting as activities for both boys and girls. Males were more likely than females to rate cycling as an activity for girls. | Moderate |
| 30 | Miller et al. (2014) (30) ^b^ | India  Mumbai (urban) | Evaluate the effect of a bystander intervention on gender norms and attitudes about violence against women among middle-school aged male cricket players. | Quantitative  Quasi-experimental | Social cognitive theory | N=663; ages 10-16; males; higher SES; >75% Hindu. | School-based survey  T-tests and linear mixed effects regression models | Gender-equitable attitudes increased among participants receiving the intervention compared to participants from control schools. There were no significant changes in attitudes about violence against women. | Low |
| 31 | Ndobo (2013) (31) | France  West  (urban) | Describe gender role beliefs relating to career aspirations. | Quantitative  Randomized controlled trial | Social role theory and theory of enunciation | N=168; ages 13-17 mean age = 14.8; males and females; SES not described. | School-based survey  Chi squared | There were no differences between males and females in the assignment of gender categories to occupations. Females were more likely than boys to indicate than men and women can hold the same jobs and pursue the same careers. | Moderate |
| 32 | Nelson & Keith (1990) (32) | United States  Michigan (urban and rural) | Assessed gender differences in the development of sex role attitudes and influence of environmental variables. | Quantitative  Cross-sectional | Ecological systems theory | N=285; 5^th^ to 8^th^ grade students; males and females; SES somewhat higher than general population. | Household-based interviewer-administered survey  t-tests and hierarchical multiple regression | For females, maternal employment and father's approval of maternal employment less likely to express traditional sex role attitudes. Mothers’ traditional attitudes were associated with less traditional female participant attitudes. Parental variables were not associated with male participants’ sex role attitudes, except for fathers’ perception of child’s pubertal age, which was associated with less traditional sex role attitudes. | Moderate |
| 33 | Park et al. (2012) (33) | South Korea  Soeul  (suburban) | Assess gender differences in gender expectations and attitudes about parent restrictions on gender-inconsistent behaviors. | Quantitative  Cross-sectional | Social domain theory and social cognitive development | N=128; mean age = 11.3; males and females; middle income. | School-based interviewer-administered survey  MANOVA | Female participants were more likely than male participants to support gender-inconsistent activities. Female participants were also more likely to agree that parents should not restrict gender-inconsistent activities. | Moderate |
| 34 | Payne (1986) (34) | Barbados | Assess gender differences in attitudes toward adolescent behaviour problems and compare to data from the United States | Quantitative  Cross-sectional | *Not described* | N=297; ages 14-15; males and females; SES not described. | School-based self-administered survey  Correlations | Compared to male participants, female participants rated boys' problem behaviors as more severe. Female and male participants assigned similar seriousness to girls' problem behaviors. Comparison to secondary data from American 8th graders indicated that responses were correlated. | Moderate |
| 35 | Reis, Callahan & Goldsmith (1994) (35) | United States  Virginia  (urban and rural) | Examine gendered attitudes among gifted students about future education, career, and family. | Quantitative  Cross-sectional | *Not described* | N=284; 6^th^ to 8^th^ grade students; males and females; one quarter received financial aid; mostly white. | Summer program-based survey  Chi squared | Males were more likely than females to think that women with children should not work. | Low |
| 36 | Richmond-Abbott (1984) (36) | United States  Washtenaw County, Michigan *(urbanicity not described)* | Assess whether single mothers and their children have more liberal sex role attitudes. | Quantitative  Cross-sectional | Freudian theory, social learning theory, and cognitive-developmental theory | N=163; Mostly ages 8-14; males and females; divorced parents with relatively high education but lower income; | Mail-based Self-administered survey and household-based in-depth interviews  Correlations | Males had more traditional sex role attitudes compared to females. Participants with highly educated and non-absent parents had more liberal sex role attitudes. Females who spend time with an absent father had less traditional sex role attitudes while this had no association with males’ sex role attitudes. Mothers’ sex role attitudes are associated with their children’s sex role attitudes. | Low |
| 37 | Riemer & Visio (2003) (37) | United States  Midwest (urban) | Assess sex-typing of sports. | Quantitative  Cross-sectional | Ego developmental theory | N=?* (total sample N=365 for grades K-12); 4^th^ to 6^th^ grade students; males and females; upper-middle class; mostly white. | School-based self-administered survey  Loglinear analysis | Males and females gave similar sex-typed ratings of sports. | Moderate |
| 38 | Schuette & Killen (2009) (38) | United States  (suburban) | Examine children’s interpretations of parents’ gendered chore assignments. | Quantitative  Cross-sectional | Social cognitive domain model | N=40*; mean age = 10.5; males and females; middle class; mostly white and Hawaiian | School-based interviewer-administered survey  ANOVA | Female participants were overall more likely than male participants to choose a non-gender stereotypic parental assignment of household chores (e.g. justifying “female” tasks to sons). Fifth-grade female participants were also more likely than males to use moral reasoning (supporting gender equality) and less likely to use social conventional reasoning (supporting gender stereotypes) to justify their choice. | Moderate |
| 39 | Scott (1984) (39) | United States  Florida *(urbanicity not described)* | Evaluate the effect of a 20 lesson early adolescent school-based intervention on sex role flexibility, decision-making, and academic achievement. | Quantitative  Quasi-experimental | Cognitive developmental theory of sex role learning | N=864; 6^th^ to 8^th^ grade students; males and females; mostly low and middle income; mostly white and African American. | School-based survey  ANCOVA | Intervention participants were more likely to have egalitarian attitudes about gender roles. | Low |
| 40 | Serbin (1993) (40) | Canada  East  (urban) | Understand sex typing in middle childhood including influence of environmental and cognitive factors. | Quantitative  Cross-sectional and longitudinal cohort with 1 year follow-up | Integrative theory of sex-role development drawing on cognitive-developmental, schematic-processing, and social learning theories | N=~180*; ages 9-13; males and females; mixed SES; mostly white. | School-based survey  ANOVA and regression | Males and females had similar knowledge of gender trait stereotypes. Fathers’ education was not associated with gender trait flexibility but participants whose mothers had higher status occupations were more flexible about gender trait stereotypes. | Moderate |
| 41 | Shamai (1994) (41) | Israel  Golan region (urban and rural) | Evaluate effect of teacher training intervention on students’ occupational gender biases. | Quantitative  Quasi-experimental | *Not described* | N=271; age 12; males and females; middle class; mostly Sephardic and Ashkenazy Jews. | School-based self-administered survey  t-tests | Females tended to be less stereotypical than males in the gender ratings of occupations. | Low |
| 42 | Signorielli & Lears (1992) (42) | United States  Mid-Atlantic  (*urbanicity not descried*) | Assess association between viewing television and children’s sex role attitudes and behaviors. | Quantitative  Cross-sectional | Cognitive developmental theory and social learning theory | N=530; 4^th^ and 5^th^ grade students; males and females; SES not described; mostly white and African American. | School-based self-administered survey  Correlations and regression | Overall, males and participants who spent more time watching television had more gender stereotyped responses about who should do certain household chores. Specifically, males had more stereotypical responses if they watched more television and did masculine chores and less stereotypical responses if they did feminine chores and had parents with higher occupational status. Females had less stereotypical responses if they did masculine chores, had a higher reading level, and had more highly educated parents. | Moderate |
| 43 | Smith & Russell (1984) (43) | Australia  Sydney  (suburban and semi-urban) | Compare males’ and females’ beliefs about sex differences. | Quantitative  Cross-sectional | Cognitive developmental theory and gender schema theory | N=115*; age 10; males and females; SES not described. | School-based self-administered survey  Chi squared | Males and females did not differ in their explanations of differences between boys and girls. | Low |
| 44 | Tay & Gibbons (1998) (44) | Singapore  (urban) | Assess differences in gender role ideologies by school type. | Quantitative  Cross-sectional | *Not described* | N=246; ages 12-15; males and females; mixed SES; mostly Singaporean and Chinese. | School-based self-administered survey  ANOVA, MANOVA, and chi squared | Participants attending the most elite school had the least traditional gender attitudes while participants at the least selective school had the most traditional attitudes, with possible differences by language spoken at home. | Moderate |
| 45 | Trice (2000) (45) | United States  Bulgaria  Italy  (urban) | Assess national differences in gender stereotyping of occupations among children. | Quantitative  Cross-sectional | *Not described* | N=495; ages 10-12; males and females; representative of the community. | School-based interviewer-administered survey  Statistical comparisons | Participants in Italy had the most gender-stereotyped attitudes about careers while participants in Bulgaria had the least gender-stereotyped attitudes, suggesting a gender equalizing effect of socialism. In the United States Males had more gender-stereotyped views about occupations than females. | Low |
| 46 | Valenzuela (1993) (46) | United States  Houston, Texas  (urban) | Assess relationship between gender role attitudes, educational expectations, and family background among Mexican-origin adolescents. | Quantitative  Cross-sectional | Theory of power and practice | N=97; 7^th^ and 8^th^ grade students; males and females; low SES; Hispanic. | School-based survey  Regression | Female participants had more liberal gender role attitudes than males. Greater maternal education was associated with more liberal gender role attitudes. | Low |

*Sample size specific to the current study population of interest (10-14 years)

a) The studies by Crouter et al. (2007) and McHale et al (1999, 2001, 2004) used data from the same longitudinal cohort, but at different time points and with sub-samples for different research questions.

b) The studies by Das et al. (2014) and Miller et al. (2014) use data from the same evaluation; however, the former was explorative using cross-sectional baseline data, and the latter quasi-experimental.

**Qualitative studies**

| **Study Nr** | | **Author & Year** | | **Study Setting** | | **Study Objective** | | **Study Design** | | **Theory** | | **Sampling & Sample** | | **Data Collection & Analysis** | | **Key Findings** | | **Quality** | |
| --- | --- | --- | --- | --- | --- | --- | --- | --- | --- | --- | --- | --- | --- | --- | --- | --- | --- | --- | --- |
| 47 | Allan (2009) (47) | | United Kingdom  Southern England  *(urbanicity not specified)* | | To explore how girls in one single-sex, private primary school negotiate discourses about being a "lady" with teachers, parents and peers. | | Qualitative  Ethnography | | Post-structural feminist theory | | N=25; 10-11 years at baseline; females only; primarily White, upper/middle class  Sampling from one school (single class) | | Participant observation, FDGs, photographic diaries, participative analysis sessions in a single school.  *No analytical details* | | Teachers and parents socialized girls into being “proper ladies” at the school, for examples through reprimands if they did not adhere to rules. These expectations clashed with those from peers to be “girly girls" (dress sexy, getting boyfriends). Social class interacted with gender attitudes in that girls policed each other’s "tart" and "slutty" behaviors, typically viewed as working-class. | | Low | |  |
| 48 | Ampofo (2001) (48) | | Ghana  Two communities in Eastern Ghana: Akropong (matrilineal) and Mampong (patrilineal)  (urban/semi-urban) | | To understand how adolescents recognise sex-role disparities, and from whom they learn about gender roles. Focus on differences across matrilineal and patrilineal settings. | | Qualitative  *Qualitative study design not specified* | | *Not described* | | N=30; 11-14 years; males and females; Ghanaian.  School-based sampling | | FDGs in schools (4 groups by sex and region)  Interviews were also conducted with adults in the community  *No analytical details* | | Gender norms were transmitted through reinforcement and sanctions of roles, and through the status of tasks (degradation of female chores). Boys could come and go, while girls were expected to go directly from school to assist household work. Mothers were particularly important for reinforcing norms, e.g. by keeping boys from doing female chores. Overall, girls from the matrilineal context expressed strong opinions against conventional gender norms; the same was not true for patrilineal setting. | | Low | |  |
| 49 | Archer (2001) (49) | | United Kingdom  North-west England  (semi-urban) | | Explore young Muslim adolescent constructions of “racialized” masculine identities | | Qualitative  Phenomenology | | Post-structural feminist theory | | N= 24; 14-15 years; males, mainly Pakistani or Bengali; working-class; Muslim  Purposive sampling | | FGDs in three schools (2 groups in each).  Discourse analysis | | Young Muslim adolescents constructed masculinities by locating themselves against Muslim girls, who were considered as "not real" Muslims because of their westernization. Gender norms for being “real” Muslim men, including the display of power over women, were considered as inevitable and transmitted through history and traditions. | | Strong | |  |
| 50 | Bowen et al. (50) | | Belgium  Germany  Great Britain  Sweden  *(Region and urbanicity not specified)* | | To explore universal themes and differences in attitudes and beliefs towards dating violence among adolescents across four European countries. | | Qualitative  *Qualitative study design not specified* | | Develop own theory in the form of a thematic map | | N=unclear; 11-17 years; upper/middle class; White and Asian (UK), ethnicity in the other settings not described.  School-based sampling except UK (snowball sampling). | | FGDs (4 groups, one in each country)  Thematic analysis | | Across countries, young adolescents viewed masculine norms as linked to violence perpetration, and feminine norms to victimization. Male perpetration of violence on females was seen as less acceptable than the reverse. Boys and girls reported learning about gender norms attitudes through television, for example by witnessing male perpetration of violence against girls, but not the reverse. | | Low | |  |
| 51 | Brown  (1997) (51) | | United States  Maine (rural) | | To explore how White working-class girls in rural Maine understand, express, and react to dominant cultural definitions of femininity. | | Qualitative  *Qualitative study design not specified* | | No theory specified, but the study has a sociological grounding | | N=13; 11-14 years; girls; White, lower SES.  Purposive sampling | | Individual in-depth interviews  FGDs, videotaped  Inductive analysis using the “listening guide” | | Girls held strong perceptions about the ideal girl; however, most girls had never met a girl like that and did not think that she exists. Results highlighted the impact of the peers as dictating girls construction of, and ability to resist, conventional femininity norms. For example, girls faced strong peer pressures to accept the dominant construction of femininity. | | Strong | |  |
| 52 | Denner & Dunbar (2004) (52) | | United States  Central California (semi-urban) | | To understand how Mexican-American girls perceive and negotiate femininity | | Qualitative  *Qualitative study design not specified* | | Feminist theory | | N=8; 12-14 years; all born in the US but of Mexican American decent; lower SES.  Purposive sampling | | In-depth interviews  Thematic analysis | | Four themes emerged about what it means to be a girl: 1) girls and boys are different, but equal; 2) boys have more advantages through freedom and mobility; 3) girls are strong; 4) adults try and limit girls' strength. Key socialization agents included mothers and other adults. Strategies to negotiate femininity norms included speaking up against norms, and changing femininities according to the context. | | Strong | |  |
| 53 | Eder & Parker (1987) (53) | | United States  Midwest (urban/rural) | | To understand how extracurricular activities in school influence students gender-related values and interactions | | Qualitative  Ethnography | | *Not described* | | N=unclear; grade 7-8; males and females; middle and working-class; mainly White  Sampling from one school | | Observations of  58 cheerleading sessions and 19 athletic games, other activities (e.g. choir, concerts, lunches, informal interactions)  *No analytical details* | | Extracurricular activities were found to influence male and female gender-related values. Through athletic activities, males were encouraged to be achievement oriented, competitive, and aggressive. Through cheerleading, females were encouraged to smile and be concerned about appearance. Young adolescents were also influenced through informal interactions with peers. Findings overall suggest that schools play an important role in reproducing gender differences. | | Moderate | |  |
| 54 | Hernandez Ordoñez (2006) (54) | | Mexico  Mexico City (urban) | | To understand the meaning and social construction of masculinity among homeless youth (who participate in activities provided by a team of street youth educators), and how gender norms impact social interactions | | Qualitative  Ethnography | | *Not described* | | N=10 aged 7-14 (also included two 16-17 year olds, and quotes are sometimes not disaggregated by age*)*; males and females; low SES (street/homeless children); participated in youth activities provided by the Institucion de Asistencia Privada | | Observation, conducted daily over a 8-hour period  Inductive coding and categorization | | The children and youth observed maintain the social representation or cultural beliefs of "traditional masculinity". In these beliefs predominates the idea that a man should appear to have control of themselves, most situations, and other people, and not appear 'female' (e.g. cry or show feelings). Peer norms about risk taking led to constant exposure to risky situations such as drug use and street fights. The ways that peers related to one another was overall regulated by canonical representations of masculinity and discrimination against women. | | Moderate | |  |
| 55 | Korobov (2005) (55) | | United States  Northeast (urban) | | To explore the extent and social contexts of how boys use peer-to-peer irony and verbal plays as tools to construct or resist masculinity norms. | | Qualitative  *Qualitative study design not specified* | | Feminist Theory | | N=54; 10-15 years; males; working-class; “mixed ethnicities”  Sampling from public elementary, middle, and high schools | | FGDs (videotaped)  Discourse analysis | | The boys in this study used irony to construct heteronormative positions regarding sexuality and gender. For example, peer verbal teasing and ironic plays were common strategies to police and regulate heteronormativity. | | Moderate | |  |
| 56 | Lahelma (2002) (56) | | Finland  *(Region and urbanicity not specified)* | | To explore girls and boys informal interactions in secondary school, with focus on gender-based policing and harassment. | | Qualitative  Ethnography | | *Not described* | | N=63; 13-14 years at first interview (17-19 at follow up).  Sampling from one school | | Observations  Individual interviews, and follow-up interviews.  Teachers were also interviewed.  *No analytical details* | | Students in a school expressed and constructed gender through teasing and sexual harassment, e.g. girls calling each other "whore" and boys using homophobic epithets. Girls teasing of boys were considered a threat to masculinity. Sexist comments and harassment was overall a mode for peers to police masculinities and heteronormativity. | | Low | |  |
| 57 | McMahon & Patton (1997) (57) | | Australia  Queensland, (urban) | | To explore children and adolescents knowledge of the social influences on their career development focused on gender-stereotyped career choices. | | Qualitative  *Qualitative study design not specified* | | Systems theory framework of career development | | N=55; 3-18 years; males and females; middle class  Sampling from one private school. | | FDGs  Thematic analysis | | Boys expressed greater gender stereotypes about career choices for women than girls. Specifically, boys expressed more restrictions about what careers were appropriate for women. | | Moderate | |  |
| 58 | Metton (2004) (58) | | France  Paris (urban) | | To understand online peer socialization of gender norms and identities, and to investigate the Internet as an opportunity for young peoples’ construction of alternative identities. | | Qualitative  Phenomenology | | Horizontal socialization | | N=24; 11-15 years; males and females; “diverse social backgrounds”.  Sampling from one middle school and vacation centres | | In-depth interviews  Observations  *No analytical details* | | While peers shaped gender norms through face-to-face interactions, online interactions allowed boys to speak more freely to girls. Through the Internet, adolescents learned how to “do romance” according to their respective gender norms. However, they also used the Internet as a space to sidestep the importance of physical appearance as a gender norm enforced by peers. For example, girls used male social media identities to better understand masculine gender norms. | | Low | |  |
| 59 | Moffatt & Norton (2008) (59) | | Canada  Vancouver (urban) | | To map common discourses of gender and sexuality in a diverse group of preteen students. | | Qualitative  *Qualitative study design not specified* | | Feminist post-structural theory | | N=55, grades 5-7; males and females, middle-class; multi-cultural backgrounds  Sampling from one public elementary school | | Students were asked to read and provide written comments to an *Archie* comic. An additional 10 were selected for in-depth interviews. | | Masculinities and femininities were constructed and understood via heterosexual desire. Students indicated that the Archie comic books conveyed to them the heteronormative view of sexuality, enforcing conventional gender norms for boys and girls. | | Moderate | |  |
| 60 | Mora (2012) (60) | | United States  Northeast (urban) | | To explore how a group of 6^th^ grade, second-generation immigrant Dominican and Puerto Rican boys construct their masculine identities at a middle school. | | Qualitative  Ethnography | | Feminist post-structural theory | | N=10; grade 6, second-generation Dominican and Puerto Rican; lower SES; experiencing puberty changes. | | Observations  Individual interviews  Thematic analysis | | Boys indicated receiving messages about masculinity from Latino and African-American rappers, and also aspired to the masculine qualities of wrestlers and video game characters. Fights in local neighborhoods reinforced perceptions of normative masculinity. Boys also used verbal control and teasing (mainly around body changes) to enforce each other’s heterosexual masculine identities. | | Strong | |  |
| 61 | Mora (2013) (61) | | United States  Northeast (urban) | | To explore how a group of second-generation immigrant Dominican and Puerto Rican boys invoke homosexuality to construct masculine identities at a middle school. | | Qualitative  Ethnography, longitudinal (one class followed over 2 years) | | Feminist post-structural theory | | N=33; 11-14 years; second-generation Dominican and Puerto Rican; lower SES.  Sampling from Romero Elementary and Middle School. | | Observations  Individual interviews  Thematic analysis | | Male peer groups were found to regulate and enforce hegemonic, heteronormative masculinity, for example through use of homophobic language and jokes. Latino culture as well as US pop culture reinforced these heteronormative gender values. While teachers sometimes tried to suppress the homophobia expressed in the class, but were also at time complicit in reinforcing heteronormativity. | | Strong | |  |
| 62 | O'Brien (2003) (62) | | Ireland  *(Region and urbanicity not described)* | | To understand the gendered emotional, social and academic experiences of girls from different social class as they move from primary to secondary school, with focus on how girls develop feminine identities. | | Qualitative  *Qualitative study design not specified* | | *Not described* | | N=86; 11-13 years; females; working and middle class.  Convenient sampling of *schools* (no details on student sampling) | | Individual interviews (3 times per student: end of primary school, beginning and end of 1^st^ year at secondary school)  *No analytical details* | | As girls transitioned from primary to secondary school, the gender stereotyped policing environment became more pronounced. Girls voiced that schools policed their bodies through uniforms and various rules. Responses to these rules differed by social class. For example, participation in “feminine” activities varied by social class, which may be related to parents' expectations for performance of femininities. | | Moderate | |  |
| 63 | O'Sullivan, Meyer-Bahlburg & Watkins (2000) (63) | | United States  New York City areas Washington Heights and Upper Harlem (urban) | | To examine the social cognitions that girls in late childhood and early adolescence associate with their changing sexuality during puberty. | | Qualitative  Phenomenology | | Symbolic interactionist framework | | N=57 mother-daughter pairs; girls aged 10-13 years; low SES; African-American and Latina.  Purposive sampling | | FGDs with daughters and mothers  Thematic analysis | | Girls faced increased expectations to assume adult female roles, end friendships with boys, avoid contact with older men, and dress modestly during transitions into puberty. Girls challenged parent’s attempts to restrict their behaviour by explicitly expressing adult feminine sexual roles. Parent child communication of puberty differed between the African-American and Latina families. | | Moderate | |  |
| 64 | Paechter & Clark (2007) (64) | | United Kingdom  England, London (urban) | | To investigate perceptions about “tomboys” as identified by children, parents and teachers; and how pre-pubertal girls construct tomboy norms. | | Qualitative  Case study | | *Not described* | | N=12; 9-11 years; females identified as tomboys; high and low income.  Purposive sampling from two primary schools. | | Observations  Individual in-depth interviews  FGDs  Constant comparative method | | Tomboys were seen as more willing (than ‘feminine’ girls) to fight and participate in sports – particularly football. Clothes were an important marker of being a tomboy, but in different ways due to the school environment (uniforms required vs. not), affluence, and religion (one school largely Muslim). Greater parental expectation of being feminine at the more affluence school meant that getting dirty/resisting feminine expectations was more important in this setting. | | Moderate | |  |
| 65 | Peterson (2002) (65) | | United States  Northwest Ohio (urban/sub-urban) | | To understand the gendered writing choices available to boys and girls in their classrooms. | | Qualitative  Ethnography | | *Not described* | | N=54; 13-14 years; males and females; low and middle SES; mainly African-American and European-American.  Sampling from one school | | Writing analysis  Interviews  Observation  *No analytical details* | | Gender norms were more strongly enforced in their writing of stories. Conscious of their writing being discussed in a public setting, boys wrote stories that focused on a masculine discourse, including sports and violence. Peers generally found girls’ writing about masculine topic acceptable, whereas boy’s writing on feminine topics (e.g. romantic stories) was ridiculed. | | Moderate | |  |
| 66 | Qin (2009) (66) | | United States  Boston area (urban/sub-urban) | | To understand how Chinese immigrant girls and boys discuss and negotiate different gender and ethnicity norms faced at home and school. | | Qualitative  Grounded Theory, longitudinal (5 year follow-up) | | Identity formation theory | | N=unclear (“students at 9 schools”); 12 years at first interview; males and females; middle/low SES; Chinese immigrant origin  Sampling strategy not clear | | Individual in-depth interviews  Analysis guided by Grounded Theory | | US-immigrant children of Chinese origin experienced conflicts related to gender roles at home and at school. At home, doing well in school and not being “wild” was most important for both boys and girls. At school, girls were expected to focus on appearance and male attention, and boys to perform in sports, video games, and other non-academic activities. Girls were in general more firmly rooted in their Chinese identity; the author suggests this may be because being a good Chinese girl does not conflict with American adolescent perceptions of femininity, whereas being a good Chinese boy does. | | Moderate | |  |
| 67 | Ragnarsson et al. (2008) (67) | | South Africa  Mankweng, Limpopo province (rural) | | To explore how young adolescent boys and girls depict and interpret male gender and sexuality in their community. | | Qualitative  *Qualitative study design not specified* | | Ecological systems theory | | N=72; 12-14 years; males and females; low SES  Convenient sampling within schools that in turn were randomly selected | | FGDs (7 groups with males and females respectively, 4 mixed-sex groups)  Latent content analysis | | Males openly discussed the role of circumcision and initiation rites, with the view that it legitimizes the onset of penetrative sex for boys. All participants emphasized male sexuality as biologically predetermined, rationalizing multiple partners and sexual violence. Girls highlighted that giving in to men's sexual advances were better than being beaten for resisting, and that reporting abuse lead to blame from parents and police. Male participants further felt that they must be able to provide for a girl in order to have sex with her, and it was noted that girls often go with older men because they can give them gifts. | | Strong | |  |
| 68 | Renold (2006) (68) | | United Kingdom  Eastern England (semi-rural) | | To illustrate how children become gendered and negotiate the hegemonic heterosexual matrix within an institutional and generational space. | | Qualitative  Ethnography | | Feminist post-structural theory, Queer theory | | N=unclear; 10-11 years; males and females; low and middle SES; White.  Sampling of two classes from two primary schools | | Participant observation  Individual interviews  *No analytical details* | | Results revealed that taking up the position of being a boy/girlfriend was the only legitimate way to be friends with the opposite sex. Taking the identity of “tomboy” did however enable some girls to maintain opposite-sex friendship with no discussion of romance. Gendered bullying and harassment were the central means by which children created gender and sexual norms. | | Moderate | |  |
| 69 | Renold & Ringrose (2008) (69) | | United Kingdom  Wales (urban) | | To explore the “regulation and rupture of Butler’s heterosexual matrix” among girls, and how dominant versions of tweenage and teenage femininity are reworked in race and class specific ways. | | Qualitative  Ethnography | | Feminist post-structural theory, Queer theory | | Same as Renold (2006); focus on one younger and one older group of early adolescent girls. | | FGDs  Individual interviews  Participant observation  *No analytical details* | | Girls challenged heterosexual discourses and rejected scripts of body and fashion, but in slightly different ways. The younger group of girls, all white, largely did so by dressing in comfortable clothes at the school disco, unlike girls who are trying to impress boys by wearing miniskirts. The older girls constructed a sexually appropriate identity in highly regulatory ways through performance of competitive “girlie-girl” femininities. | | Moderate | |  |
| 70 | Ribeiro (2006) (70) | | Brazil  Bahia beach/island community (rural) | | To explore how poor, black youth construct female sexuality and gender norms, as well as how parents'/relatives' gendered discourse and actions impact these beliefs. | | Qualitative  Ethnography | | Not described | | N=unclear; 7-14 years; males and females; Black; low SES.  Sampling not described | | Interviews  Observation  *No analytical details* | | For the young adolescents interviewed, being a man or woman was associated not only with anatomical-physiological features, but with social conceptions, many learned in the family and in the system of [gender] relations in which they live. The category man and woman, in this case boy and girl, involved social and symbolic attributes, such as being able to do certain things, exercise sexuality legitimately, and engage in behaviors within a particular [gendered] order/system. | | Low | |  |
| 71 | Ringrose et al. (2013) (71) | | United Kingdom  England, London (urban) | | To gain insights into young adolescents social media experiences and practices focused on sexting (nude pictures and sexually explicit pins). | | Qualitative  Narrative research | | Feminist theory | | N=35; 12-15 years; males and females; mixed SES.  Purposive and critical case sampling | | FGDs  Individual interviews  *No analytical details* | | Boys accumulated status by possessing and exchanging nude images of girls. In contrast, girls considered sharing such images as risky, potentially inciting shame around sexual reputation (e.g. being called ‘slut’). Yet, being asked for an image of one’s body carried social value among girls, and constituted a norm of feminine desirability within the digital teen peer network. | | Moderate | |  |
| 72 | Skelton, Francis & Read (2010) (72) | | UK  Southeast and middle Britain (urban/rural) | | Explore how high achieving pupils produce academic success alongside acceptable masculinities/femininities. | | Qualitative  Ethnography | | Feminist post- structural theory, Queer theory | | N=71; 12-13 years; females; low/middle SES; mainly White.  Sampling from 9 secondary schools | | Interviews  Participant observation  Content and Foucaldian analysis of discourse | | The majority of high achieving girls positioned themselves within the boundaries of conventional femininities, which meant substantial investment in their relationships. Girls struggled with balancing academic achievement and social acceptance and approval of classmates (i.e. conventional femininity norms). | | Moderate | |  |
| 73 | Stern et al. (2003) (73) | | Mexico  Mexico city neighborhoods Mesa de Hornos and San Miguel Teotongo (urban) | | To understand how the social construction and expression of masculinity between male adolescents and young men are linked to sexual and reproductive health risks. | | Qualitative  Grounded Theory (implicit) | | Building own theory | | 10-24 years; males; mixed SES (urban ‘popular’ vs. urban ‘marginal’ areas); Mexican.  Sampling through schools and health service agencies. | | Individual interviews  FGDs  Grounded Theory (implicit) | | The males expressed ideals that sustained the "traditional" idea of what it means to "be a man". Males initiated sex to prove adult masculinity, but were however largely uninformed about reproductive health issues and are anxious/uncomfortable speaking about ways to protect [yourself sexually] with their female 'friends' and girlfriends. The results illustrate the importance of developing interventions that are tailored to the specific situation in which young men are socialized and that take into consideration the subjectivity of gender beliefs. | | Moderate | |  |
| 74 | Swain (2000) (74) | | United Kingdom  Southern England (rural/sub-urban) | | To explore how football (soccer) is used to construct and negotiate gender identities. | | Qualitative  Ethnography | | Theory of Gender and Power | | N=unclear; 10-11 years; males; middle SES; mainly White. | | FGDs (25 groups with 3 boys in each)  Participant observation (one class)  *No analytical details* | | Males constructed hegemonic masculinities in relation to other masculinities and femininities, which necessarily become subordinated and marginalized. The dominant group of boys performed different aspects of masculinity in different spaces during the school day. Football/soccer reinforced the performance of hegemonic masculinity, and stigmatized boys in the “subordinate group”. | | Moderate | |  |
| 75 | Tischler & McCaughtry (2011) (75) | | United States  Midwest (sub-urban) | | To examine the intersection of masculinity and school physical education from the perspective of boys who embody marginalized masculinities | | Qualitative  Ethnography | | Theory of Gender and Power (reconceptualised) | | N=unclear; middle school; males; about 80% White.  Purposive sampling (Boys were selected from PE classes if they displayed “guarded” behaviour) | | Interviews  Observation  Thematic analysis, constant comparison | | Boys made sense of masculinities in the context of physical education entered on content taught; pedagogical practices; relationships between teachers and students, peer social cultures. PE was dominated by masculine sports at the schools, whereby teachers privileging the boys who were athletic and competitive. Direction and type of teacher attention played an important role in constructing masculinities. Boys who were not able to live up to such norms described negative relationships with PE teachers, as well as harassments from peers. | | Strong | |  |
| 76 | Vu, Murries, Gonzalez & Jobe (2006) (76) | | United States  Baltimore, Tuscon, DC, New Orleans, San Diego, Minneapolis, Columbia  *(urbanicity not described)* | | To identify the similarities and differences in girls’ and boys’ perceptions of girls’ physical activity behaviors. | | Qualitative  Narrative research | | Social ecology theory | | N=80; 12-15 years; males and females; mixed race.  Purposive sampling | | Individual interviews (girls only)  FGDs (boys and girls)  Thematic analysis | | Both males and females talked about physically active girls as “tomboys”; females were more likely to have favourable views towards active girls, and males tended to be more uncomfortable with this. Boys and families were mentioned as barriers for girls’ physical activity in girls. Females talked about taunting from boys as being more important, while males were more likely to talk about girls posing restrictions on their own activity. | | Moderate | |  |
| 77 | Way et al. (2014) (77) | | United States  New York City (urban) | | To examine the form and patterns of resistance to masculinity and femininity norms among young adolescent boys | | Qualitative longitudinal  Narrative research | | *Not described* | | N=55; 11-13 years; males; mixed race | | In-depth interviews  The Listening Guide and open coding | | The majority of boys interviewed demonstrated moderate to high levels of resistance in at least one interview, typically 6th or 8th grade. Of those, 20% resisted in explicit forms while the rest resisted implicitly. There were also ethnic/racial differences in trajectories of implicit resistance. Latino youth were more likely to reveal resistance over time, while Black, white and Chinese-American youth were less likely to maintain resistance | | Moderate | |  |

*Sample size specific to the current study population of interest (10-14 years)

c) While the studies by Mora (2012, 2013) origin from the same data, the earliest one was a cross-sectional ethnography and the latter a longitudinal follow-up with a larger sample.

d) The studies by Renold (2006, 2008) use the same data source, but with different samples (the latter study focused on one younger and one older group of early adolescent girls).

**Mixed-methods studies**

| 78 | Baric (2013) (78)  Additional information retrieved from  Miske Witt & Associates, Inc. (2011) (79) | Egypt Honduras  India  Malawi  Tanzania  Yemen  *(urbanicity not described)* | To evaluate the *Power To Lead Alliance (PTLA)* project focused on changing gender norms and attitudes among young adolescents, early in their socialization process | Mixed-methods  Programmatic evaluation (pre-post test with control group) | No specific theory described, but the project developed the “CARE Gender Empowerment Framework” | N=1946 (Intervention: 753 females, 255 males; Control: 724 females, 214 males) 10-14 years; males and females; low SES.  Sampling varied across sites (random in some, non-random in others) | Qualitative: FGDs, individual interviews, observations of 18 activities, interviews with community leaders and staff  Quantitative: survey (questionnaire), including a Gender Equity index  Chi-square and t-tests | CARE implemented the *PTLA* intervention to strengthen vulnerable girls leadership opportunities through activities such as music, debate, sports, arts, drama, youth councils, life skills, awareness campaigns, and academic clubs. Overall, results from the non-randomized pre-post evaluation indicate that the intervention had some effect on gender equitable attitudes among young adolescents: higher % of both girls and boys from active groups across sites agreed with gender equitable statements than those in comparison sites. Qualitative findings further revealed that girls experienced greater respect from boys, and that boys indicated more interest to learn about domestic chores. | Moderate |
| --- | --- | --- | --- | --- | --- | --- | --- | --- | --- |
| 79 | Esiet et al. (2009) (80) | Nigeria  Lagos state (urban and rural) | Assess changes in adolescents’ gender equitable attitudes before and after receiving a Family Life and HIV Education curriculum in school. | Mixed-methods  Quasi-experimental | Not described | N=1,563; ages 10-19 mean age = 12.1; males and females; SES not described. | Qualitative: Focus group interviews (used to elaborate on quantitative findings)  Quantitative: School-based survey, Chi square tests | Gender equitable attitudes increased over the course of the school year for both males and females. At the beginning of the school year gender equitable attitudes were similar for males and females while at the end of the year females espoused more equitable attitudes than males. Qualitative findings suggest that participants believed that changes in attitudes were related family influences and to the curriculum. | Low |
| 80 | Granié & Papafava (2011) (81) | France  Southeast  *(urbanicity not described)* | Assess age differences in gender stereotypes about driving. | Mixed-methods  Cross-sectional, Grounded theory | Not described | N=599; ages 10-16 mean age = 12.2; males and females; mostly middle class or white-collar families. | Qualitative: Themes derived using free-listing    Quantitative: School-based self-administered survey  T-tests | Eight themes about driving qualities were derived: good, bad, careful, careless, compliant, offending drivers, and consistency between driving and masculine vs. feminine role. Females were more likely than males to attribute negative qualities (careless, getting traffic offences) to male drivers and some positive qualities (careful) to female drivers. Females were also more likely to indicate that males promote their own masculinity through driving. | Low |
| 81 | Lundgren et al. (2013) (82) | Nepal  Siraha district, Terai region (rural) | To evaluate whether participation in the *Choices* curriculum resulted in a change in early adolescent attitudes to gender issues, and whether this in turn led to behaviour changes. | Mixed-methods  Programmatic evaluation (matched pre-post evaluation with control group) | Not described | N=603 (intervention: 309, control: 294); 10-14 years; males and females; low SES.  Sampling from child clubs for both intervention and control groups (matching) | Qualitative: In-depth interviews (N=36), Photovoice (N=24), FGDs with parents  Thematic analysis  Quantitative: questionnaire with card games, pile sorts and vignettes.  T-tests | Save the Children implemented the *Choices* curriculum with the goal to transform gender norms and behaviors among young adolescents through weekly 2-hour child club activities over 3 months. Evaluation results indicate that intervention participants expressed more liberal attitudes toward women's independence and non-traditional behaviors and were more accepting of non-traditional gender roles. Attitudes in the control group did not change. Qualitative findings revealed that Choices participants were more confident to “discuss their feelings and promote gender equality”, and boys were more willing to engage in and help out with household work. | Low |
| 82 | Stiles (1990) (83) | United States  Mexico  St Louis (US) and Mexico City (Mexico) | To understand young adolescents’ attitudes about the ideal man or woman in two culturally different contexts (USA and Mexico). | Mixed-methods  Cross-sectional | Not described | N=188 (USA: 99, Mexico: 89); 9^th^ grade (mode age 14 years); males and females; middle/high SES  Sampling from single-sex, private, affluent Catholic schools | Qualitative: drawings of the ideal man vs. woman.  The study team rated the stereotypicality of the drawings.  Quantitative: survey with ranking process of opposite-sex ideals  Friedman's test and Tukey's test | Males placed higher importance on good looks and sexiness for the ideal woman, compared to females rated for the ideal man. Females rated kindness/honesty and professional success higher for the ideal man than boys did for the ideal woman. More boys than girls drew the ideal woman as physically mature and sexually attractive. More girls than boys drew the ideal man in a sex-stereotypical activity. Mexican adolescents portrayed more sex-stereotyped activities in the drawings compared to US participants. | Low |

**References**

1. Baruch GK, Barnett RC. Fathers' participation in family work and children's sex-role attitudes. Child development. 1986;57(5):1210-23.

2. Blakemore JEO. Children's beliefs about violating gender norms: Boys shouldn't look like girls, and girls shouldn't act like boys. Sex Roles. 2003;48(9-10):411-9.

3. Bohannon JR, Blanton PW. Gender role attitudes of American mothers and daughters over time. The Journal of social psychology. 1999;139(2):173-9.

4. Bos HMW, Picavet C, Sandfort TGM. Ethnicity, gender socialization, and children’s attitudes toward gay men and lesbian women. Journal of Cross-Cultural Psychology. 2012;43(7):1082-94.

5. Boxley J, Lawrance L, Gruchow H. A preliminary study of eighth grade students' attitudes toward rape myths and women's roles. The Journal of school health. 1995;65(3):96-100.

6. Brown JD, L'Engle KL. X-rated: Sexual attitudes and behaviors associated with U.S. early adolescents' exposure to sexually explicit media. Communication Research. 2009;36(1):129-51.

7. Cowan G, Campbell RR. Rape causal attitudes among adolescents. Journal of Sex Research. 1995;32(2):145-53.

8. Crouter AC, Whiteman SD, McHale SM, Osgood DW. Development of gender attitude traditionality across middle childhood and adolescence. Child development. 2007;78(3):911-26.

9. Das M, Ghosh S, Verma R, O'Connor B, Fewer S, Virata MC, et al. Gender attitudes and violence among urban adolescent boys in India. International Journal of Adolescence and Youth. 2014;19(1):99-112.

10. de Lemus S, Moya M, Glick P. When contact correlates with prejudice: Adolescents’ romantic relationship experience predicts greater benevolent sexism in boys and hostile sexism in girls. Sex Roles. 2010;63(3-4):214-25.

11. Emihovich CA, Gaier EL, Cronin NC. Sex-role expectations changes by fathers for their sons. Sex Roles. 1984;11(9-10):861-8.

12. Evertsson M. The reproduction of gender: housework and attitudes towards gender equality in the home among Swedish boys and girls. The British journal of sociology. 2006;57(3):415-36.

13. Foshee VA, Bauman KE. Gender stereotyping and adolescent sexual behavior: A test of temporal order. Journal of Applied Social Psychology. 1992;22(20):1561-79.

14. Funk JB, Buchman DD. Children's perceptions of gender differences in social approval for playing electronic games. Sex Roles. 1996;35(3-4):219-32.

15. Galambos NL, Almeida DM, Petersen AC. Masculinity, femininity, and sex role attitudes in early adolescence: exploring gender intensification. Child development. 1990;61(6):1905-14.

16. Grose RG, Grabe S, Kohfeldt D. Sexual education, gender ideology, and youth sexual empowerment. J Sex Res. 2014;51(7):742-53.

17. Hertzog JL, Rowley RL. My beliefs of my peers' beliefs: exploring the gendered nature of social norms in adolescent romantic relationships. Journal of interpersonal violence. 2014;29(2):348-68.

18. Hess M, Ittel A, Sisler A. Gender-specific macro- and micro-level processes in the transmission of gender role orientation in adolescence: The role of fathers. European Journal of Developmental Psychology. 2014;11(2):211-26.

19. Hoover R, Fishbein HD. The development of prejudice and sex role stereotyping in white adolescents and white young adults. Journal of Applied Developmental Psychology. 1999;20(3):431-48.

20. Jones LR, Fries E, Danish SJ. Gender and ethnic differences in body image and opposite sex figure preferences of rural adolescents. Body image. 2007;4(1):103-8.

21. Katz PA, Ksansnak KR. Developmental aspects of gender role flexibility and traditionality in middle childhood and adolescence. Developmental psychology. 1994;30(2):272-82.

22. Kurtz-Costes B, Rowley SJ, Harris-Britt A, Woods TA. Gender Stereotypes about Mathematics and Science and Self-Perceptions of Ability in Late Childhood and Early Adolescence. Merrill-Palmer Quarterly: Journal of Developmental Psychology. 2008;54(3):386-409.

23. Levant RF, Graef ST, Smalley KB, Williams C, McMillan N. Evaluation of the Psychometric Properties of the Male Role Norms Inventory-Adolescent (MRNI-A). Thymos: Journal of Boyhood Studies. 2008;2(1):46-59.

24. Liben LS, Bigler RS. The developmental course of gender differentiation: conceptualizing, measuring, and evaluating constructs and pathways. Monographs of the Society for Research in Child Development. 2002;67(2):i-viii, 1-147; discussion 8-83.

25. Lurye LE, Zosuls KM, Ruble DN. Gender identity and adjustment: understanding the impact of individual and normative differences in sex typing. New directions for child and adolescent development. 2008(120):31-46.

26. McHale SM, Crouter AC, Tucker CJ. Family context and gender role socialization in middle childhood: comparing girls to boys and sisters to brothers. Child development. 1999;70(4):990-1004.

27. McHale SM, Updegraff KA, Helms-Erikson H, Crouter AC. Sibling influences on gender development in middle childhood and early adolescence: a longitudinal study. Developmental psychology. 2001;37(1):115-25.

28. McHale SM, Kim JY, Whiteman S, Crouter AC. Links between sex-typed time use in middle childhood and gender development in early adolescence. Developmental psychology. 2004;40(5):868-81.

29. Meaney KS, Dornier LA, Owens MS. Sex-role stereotyping for selected sport and physical activities across age groups. Perceptual and motor skills. 2002;94(3 Pt 1):743-9.

30. Miller E, Das M, Tancredi DJ, McCauley HL, Virata MCD, Nettiksimmons J, et al. Evaluation of a gender-based violence prevention program for student athletes in Mumbai, India. Journal of interpersonal violence. 2014;29(4):758-78.

31. Ndobo A. Discourse and attitudes on occupational aspirations and the issue of gender equality: What are the effects of perceived gender asymmetry and prescribed gender role? European Review of Applied Psychology. 2013;63(4):231-41.

32. Nelson C, Keith J. Comparisons of female and male early adolescent sex role attitude and behavior development. Adolescence. 1990;25(97):183-204.

33. Park Y, Lee‐Kim J, Killen M, Park K, Kim J. Korean children's evaluation of parental restrictions regarding gender‐stereotypic peer activities. Social Development. 2012;21(3):577-91.

34. Payne MA. West Indian teachers', parents', and students' attitudes toward adolescent behavior problems. Adolescence. 1986;21(81):235-45.

35. Reis SM, Callahan CM. Attitudes of Adolescent Gifted Girls and Boys toward Education, Achievement, and the Future. Gifted Education International. 1994;9(3):144-51.

36. Richmond-Abbott M. Sex-role attitudes of mothers and children in divorced, single-parent families. Journal of Divorce. 1984;8(1):61-81.

37. Riemer BA, Visio ME. Gender typing of sports: an investigation of Metheny's classification. Research quarterly for exercise and sport. 2003;74(2):193-204.

38. Schuette C, Killen M. Children's evaluations of gender-stereotypic household activities in the family context. Early Education and Development. 2009;20(4):693-712.

39. Scott KP. Effects of an intervention on middle school pupils' decision making, achievement, and sex role flexibility. The Journal of Educational Research. 1984;77(6):369-75.

40. Serbin LA, Powlishta KK, Gulko J. The development of sex typing in middle childhood. Monographs of the Society for Research in Child Development. 1993;58(2):1-99.

41. Shamai S. Possibilities and limitations of a gender stereotypes intervention program. Adolescence. 1994;29(115):665-80.

42. Signorielli N, Lears M. Children, Television, and Conceptions about Chores: Attitudes and Behaviors. Sex Roles. 1992;27(3-4):157-70.

43. Smith J, Russell G. Why do males and females differ? Children's beliefs about sex differences. Sex Roles. 1984;11(11-12):1111-20.

44. Tay LS, Gibbons JL. Attitudes toward Gender Roles among Adolescents in Singapore. Cross-Cultural Research. 1998;32(3):257-78.

45. Trice AD. Italian, Bulgarian, and U.S. children's perceptions of gender-appropriateness of occupations. The Journal of social psychology. 2000;140(5):661-3.

46. Valenzuela A. Liberal gender role attitudes and academic achievement among Mexican-origin adolescents in two Houston inner-city Catholic schools. Hispanic Journal of Behavioral Sciences. 1993;15(3):310-23.

47. Allan AJ. The importance of being a 'lady': hyper-femininity and heterosexuality in the private, single-sex primary school. Gender and Education. 2009;21(2):145-58.

48. Ampofo AA. "When men speak women listen": gender socialisation and young adolescents' attitudes to sexual and reproductive issues. African journal of reproductive health. 2001;5(3):196-212.

49. Archer L. 'Muslim brothers, black lads, traditional Asians': British Muslim young men's constructions of race, religion and masculinity. Feminism & Psychology. 2001;11(1):79-105.

50. Bowen E, Holdsworth E, Leen E, Sorbring E, Helsing B, Jaans S, et al. Northern European adolescent attitudes toward dating violence. Violence and victims. 2013;28(4):619-34.

51. Brown LM. Performing femininities: Listening to white working-class girls in rural Maine. Journal of Social Issues. 1997;53(4):683-701.

52. Denner J, Dunbar N. Negotiating femininity: Power and strategies of Mexican American girls. Sex Roles. 2004;50(5-6):301-14.

53. Eder D, Parker S. The Cultural Production and Reproduction of Gender: The Effect of Extracurricular Activities on Peer-Group Culture. Sociology of Education. 1987;60(3):200-13.

54. Herńandez Ordoñez A. Social representation of male gender in a group of children and youth living in the streets in Mexico City. Part two. Salud Ment. 2006;29(1):56-63.

55. Korobov NB. 'Hetero-normative masculinity' as double-edged discourse: A discursive psychological investigation of how adolescent males negotiate their social identities in conversational interaction. US: ProQuest Information & Learning; 2004.

56. Lahelma E. Female paths to adulthood in a country of 'genderless gender'. Gender and Education. 2012;24(1):1-13.

57. McMahon M, Patton W. Gender Differences in Children and Adolescents' Perceptions of Influences on Their Career Development. School Counselor. 1997;44(5):368-76.

58. Metton C. Pre/early adolescents' use of internet exploring social worlds from home. Reseaux. 2004;123(1):59-84.

59. Moffatt L, Norton B. Reading Gender Relations and Sexuality: Preteens Speak Out. Canadian Journal of Education. 2008;31(1):102-23.

60. Mora R. “Do it for all your pubic hairs!”: Latino boys, masculinity, and puberty. Gender & Society. 2012;26(3):433-60.

61. Mora R. "Dicks are for Chicks": Latino Boys, Masculinity, and the Abjection of Homosexuality. Gender and Education. 2013;25(3):340-56.

62. O'Brien M. Girls and transition to second-level schooling in Ireland: 'Moving on' and 'moving out'. Gender and Education. 2003;15(3):249-67.

63. O'Sullivan LF, Meyer-Bahlburg HFL, Watkins BX. Social Cognitions Associated with Pubertal Development in a Sample of Urban, Low-Income, African-American and Latina Girls and Mothers. Journal of Adolescent Health. 2000;27(4):227-35.

64. Paechter C, Clark S. Who are tomboys and how do we recognise them? Women's Studies International Forum. 2007;30(4):342-54.

65. Peterson S. Gender Meanings in Grade Eight Students' Talk about Classroom Writing. Gender and Education. 2002;14(4):351-66.

66. Qin DB. Being 'good' or being 'popular': Gender and ethnic identity negotiations of Chinese immigrant adolescents. Journal of Adolescent Research. 2009;24(1):37-66.

67. Ragnarsson A, Onya HE, Thorson A, Ekstrom AM, Aaro LE. Young males' gendered sexuality in the era of HIV and AIDS in Limpopo Province, South Africa. Qualitative health research. 2008;18(6):739-46.

68. Renold E. "They Won't Let Us Play ... Unless You're Going out with One of Them": Girls, Boys and Butler's "Heterosexual Matrix" in the Primary Years. British Journal of Sociology of Education. 2006;27(4):489-509.

69. Renold E, Ringrose J. Regulation and rupture: Mapping tween and teenage girls' resistance to the heterosexual matrix. Feminist Theory. 2008;9(3):313-38.

70. Ribeiro JSB. Girl's and Boy's Games: Socialization, Sexuality and Gender among Children and the Social Construction of the Differences. Cadernos pagu. 2006(26):145-68.

71. Ringrose J, Harvey L, Gill R, Livingstone S. Teen girls, sexual double standards and 'sexting': Gendered value in digital image exchange. Feminist Theory. 2013;14(3):305-23.

72. Skelton C, Francis B, Read B. "Brains before "Beauty"?" High Achieving Girls, School and Gender Identities. Educational Studies. 2010;36(2):185-94.

73. Stern C, Fuentes-Zurita C, Lozano-Trevino LR, Reysoo F. Masculinity and sexual and reproductive health: a case study among adolescents of Mexico City. La salud de adolescentes. 2003;45(El Colegio de Mexico, A.C. Centro de Estudios Sociologicos, Camino al Ajusco No. 20, Colonia Pedregal de Santa Teresa, 10740, Mexico, D.F., Mexico.):S34-S43.

74. Swain J. 'The Money's Good, The Fame's Good, The Girls Are Good': The Role of Playground Football in the Construction of Young Boys' Masculinity in a Junior School. British Journal of Sociology of Education. 2000;21(1):95-109.

75. Tischler A, McCaughtry N. PE is not for me: when boys' masculinities are threatened. Research quarterly for exercise and sport. 2011;82(1):37-48.

76. Vu MB, Murrie D, Gonzalez V, Jobe JB. Listening to Girls and Boys Talk about Girls' Physical Activity Behaviors. Health Education & Behavior. 2006;33(1):81-96.

77. Way N, Cressen J, Bodian S, Preston J, Nelson J, Hughes D. “It might be nice to be a girl... Then you wouldn’t have to be emotionless”: Boys' resistance to norms of masculinity during adolescence. Psychology of Men & Masculinity. 2014;15(3):241-52.

78. Baric S. Where the boys are: Engaging young adolescent boys in support of girls' education and leadership. Gender Dev. 2013;21(1):147-60.

79. Miske Witt and Associates. The Power to Lead Alliance (PTLA): Empowering Girls to Learn and Lead Final Evaluation Report for CARE USA. Saint Paul, Minnesota, USA: CARE USA

USAID, 2011 Contract No.: M/OAA/GRO/EGAS-08-108.

80. Esiet AO, Esiet U, Philliber S, Philliber WW. Changes in knowledge and attitudes among junior secondary students exposed to the family life and HIV education curriculum in Lagos State, Nigeria. African journal of reproductive health. 2009;13(3):37-46.

81. Granié M-A, Papafava E. Gender stereotypes associated with vehicle driving among French preadolescents and adolescents. Transportation Research Part F: Traffic Psychology and Behaviour. 2011;14(5):341-53.

82. Lundgren R, Beckman M, Chaurasiya SP, Subhedi B, Kerner B. Whose turn to do the dishes? Transforming gender attitudes and behaviours among very young adolescents in Nepal. Gender Dev. 2013;21(1):127-45.

83. Stiles DA. Opposite-Sex Ideal in the U.S.A. and Mexico as Perceived by Young Adolescents. Journal of Cross-Cultural Psychology. 1990;21(2):180-99.
